# Repurposed organoselenium tethered amidic acids as apoptosis inducers in melanoma cancer *via* P53, BAX, caspases-3, 6, 8, 9, BCL-2, MMP2, and MMP9 modulations†

**DOI:** 10.1039/d4ra02944e

**Published:** 2024-06-10

**Authors:** Saad Shaaban, Hanan A. Althikrallah, Amr Negm, Ayman Abo Elmaaty, Ahmed A. Al-Karmalawy

**Affiliations:** a Department of Chemistry, College of Science, King Faisal University Al-Ahsa 31982 Saudi Arabia sibrahim@kfu.edu.sa; b Department of Chemistry, Faculty of Science, Mansoura University 35516 Mansoura Egypt; c Medicinal Chemistry Department, Faculty of Pharmacy, Port Said University Port Said 42511 Egypt; d Department of Pharmaceutical Chemistry, Faculty of Pharmacy, Horus University–Egypt New Damietta 34518 Egypt akarmalawy@horus.edu.eg; e Pharmaceutical Chemistry Department, Faculty of Pharmacy, Ahram Canadian University 6th of October City Giza 12566 Egypt

## Abstract

Organoselenium (OSe) agents hold promise for preventing cancer due to their potential ability to fight cancer development and protect cells from oxidative damage. Herein, OSe-based maleanilic and succinanilic acids were tested to estimate their antitumor activities against fifteen cancer cell lines. Besides, their potential safety and selectivity were further investigated against two normal cell lines, namely, human skin fibroblasts (HSF) and olfactory ensheathing cell line (OEC) using the growth inhibition percentage (GI%) assay. Moreover, the apoptotic potential of the superior anticancer candidates (8, 9, 10, and 11) was evaluated against P53, BAX, Caspase-3, Caspase-6, Caspase-8, Caspase-9, BCL-2, MMP2, and MMP9 apoptotic markers. Additionally, to enhance our understanding and predict the inhibitory potential of the examined compounds as potential anticancer agents, a thorough structure–activity relationship (SAR) analysis was conducted. On the other hand, molecular docking and ADMET studies were performed for the examined candidates as well. Overall, our findings point to significant anticancer activities of the organoselenium tethered amidic acids, suggesting their promising cytotoxic potential as effective anticancer drugs.

## Abstract 2

Repurposed organoselenium tethered amidic acids as apoptosis inducers in melanoma.

## Graphical abstract

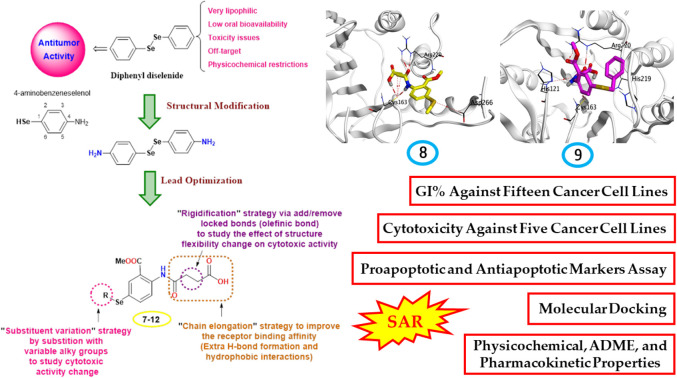

## 1. Introduction

While advancements in cancer treatment have been significant, conquering this disease remains a major challenge for healthcare systems around the world.^1^ The burden of cancer is particularly felt in countries with developing or middle-income economies, highlighting the ongoing link between socioeconomic status and cancer's impact.^2,3^ Cancer ranks as the 2nd leading cause of death worldwide, following heart disease.^4,5^ A worrying trend suggests cancer cases could surge by over 50% in the years ahead.^6,7^ Furthermore, since anticancer drugs target rapidly dividing cells, they can unfortunately damage healthy tissues as well as cancerous ones. This can lead to a range of side effects, including a weakened immune system, hair loss, anemia, and nausea.^8,9^ In response to these challenges, researchers worldwide are dedicating significant effort to developing groundbreaking therapies for a wide range of cancers.

Apoptosis, a form of controlled cell death, is overseen by two protein families: the BCL-2 family and the Caspase family. The release of cytochrome c triggers the caspase cascade, preparing it for the activation of cell death.^10^ Cells with built-in self-destruct mechanisms, known as programmed cell death, ensure our bodies eliminate old and malfunctioning cells, keeping everything in working order. Cancer cells, however, unlike normal cells, can disable this self-destruct program, allowing them to divide uncontrollably.^11^ Inside the cell's powerhouses, the mitochondria, a process called mitochondrial outer membrane permeabilization (MOMP) triggers cell suicide, also known as intrinsic apoptosis. MOMP leads to the formation of structures called apoptosomes, which activate Caspase-9. This, in turn, sets off a chain reaction involving other caspases, ultimately leading to cell death.^10^

Moreover, BAX is a key player in the BCL-2 family, a group of proteins that regulate cell death. When activated, BAX triggers MOMP, which leads to the release of pro-apoptotic proteins like cytochrome c. This release sets the stage for programmed cell death.^10^ On the other hand, P53 acts as a brake on cell growth. Located in the cell nucleus, it controls genes and promotes cell death when necessary. This makes P53 a classic tumor suppressor protein.^12^ When cells experience stress, like DNA damage, P53 accumulates in the nucleus. There, P53 acts like a tumor suppressor by triggering two important responses: cell cycle arrest (this gives the cell time to repair the damaged DNA) and apoptosis (P53 promotes cell death to prevent the spread of damaged cells, if the damage is too severe).^12^

Cyclic anhydrides are important heterocyclic scaffolds and versatile building blocks in organic synthesis, possessing a broad spectrum of uses in medicinal chemistry and materials science.^13^ They can readily react with various nucleophiles to afford diverse bifunctional intermediates used for the construction of complex molecular structures in the domains of materials science and pharmaceutical chemistry.^14^ For instance, maleic and succinic anhydrides are frequently utilized for the synthesis of dicarboxamides and cyclic imides *via* reaction with amines.^15,16^ The reaction typically involves a nucleophilic attack of the amine on the anhydride carbonyl (C=O) carbon, followed by ring opening and the formation of the corresponding amidic acid intermediates *i.e.*, *N*-maleanilic and *N*-succinanilic carboxylic acid derivatives.^17,18^ The latter is either subjected to further reaction with amines to furnish dicarboxamides or subsequent dehydration and intramolecular cyclization to give the corresponding cyclic imides (*e.g.*, maleimides and succinimides).^19^

Within this context, dicarboxamides are important synthetic intermediates and have shown prevalent applications in coordination, agrochemicals, and polymer chemistry.^16,20^ Furthermore, they exhibited anti-atherosclerotic, hemostatic, anti-inflammatory, and anticoagulant activities.^21^ Moreover, they were extensively used in coordination chemistry as efficient ligands for sensing and separation of toxic heavy metals.^22^ For example, 1,3-dicarboxamide I exhibited good antioxidant properties by increasing the expression of the cytochrome P-450 enzymes in the liver.^16,23,24^ Additionally, maleimides and succinimides are found in many natural products and pharmaceutical active drug molecules such as the farinomalein (II) natural pesticide and Zarontin (III), and oxaleimide A (IV) which are used as anticonvulsant drug candidates.^17,25,26^

**Fig. 1:**
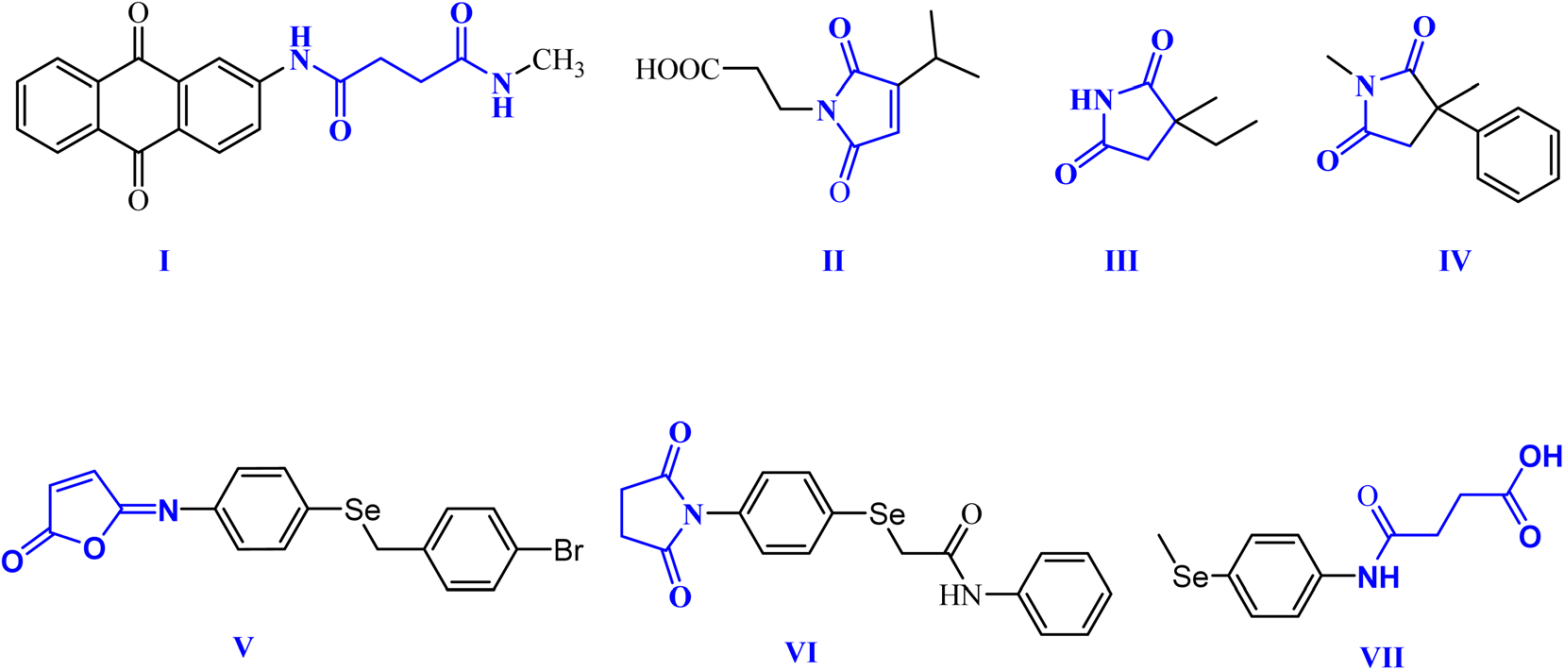
Biologically relevant amidic acid, dicarboxamide, and cyclic imides.

Furthermore, scientists are increasingly excited about developing new compounds containing selenium (Se) (organoselenium (OSe) compounds). These compounds hold promise for preventing cancer due to their potential ability to fight cancer development (chemoprevention) and protect cells from damage (antioxidant activity).^27–29^ Hence, OSe hybrids are considered privileged scaffolds in drug discovery for cancer treatment.^18,30–34^ Recently, we reported the OSe-containing isomaleimide V with good antioxidant, cytoprotective, and antiapoptotic activity against oligodendrocytes.^17,19,35^ Additionally, the OSe-bearing *N*-succinimide VI, synthesized in our laboratory, exhibited good anticancer activity against HEPG2 cells.^36^ Additionally, we disclosed OSe-containing *N*-mealanilic acid (VII) and its zinc(ii) chelate with promising antioxidant and antimicrobial activity against *Staphylococcus aureus*, *Serratia marcescens*, and *Escherichia coli* bacterial strains, and anticancer activities against different tumor cell lines (*e.g.*, MCF-7, HCT_116_, HEPG2),^37–39^Fig. 1.

Although OSe compounds manifested lower toxicity than inorganic-Se compounds; such toxicity is not enough to address the required specifications for drug development which is attributed to C–Se bond instability.^30^ Therefore, developing more stable OSe agents is of great interest. Furthermore, OSe agents induce cell death *via* multiple pathways including oxidative stress manipulation, apoptotic and antiapoptotic gene modulations, and death receptor activations, however, their specific target is still unclear.^40^ Accordingly, these challenges should be considered to better understand the underlying therapeutic potential of OSe compounds in cancer chemotherapy. The latter includes the estimation of their specific mode of action(s) and precise target(s) as well as exploring their possible use as chemosensitizers or in combination with other drugs in radiotherapy. Lately, we described OSe-bearing maleanilic and succinanilic acids as promising SARS-CoV-2 M^Pro^ inhibitors.^41^ Specifically, this preliminary study was restricted to computational calculations, and no pharmacological evaluations of the compounds were carried out to estimate their bioactivities.

Herein and as an extension of our earlier work, we aim to assess the antitumor activity of these OSe-based maleanilic and succinanilic acids against fifteen cancer cell lines. Also, their growth inhibition percentage (GI%) will be evaluated against two normal cell lines, namely, human skin fibroblasts (HSF) and olfactory ensheathing cell line (OEC), to estimate the potential safety and selectivity. Furthermore, the cytotoxic inhibitory concentration 50 (IC_50_) will be assessed against the cancer cell lines for compounds with the most outstanding GI% using the SRB assay. Moreover, the apoptotic potential of the superior anticancer candidates (8, 9, 10, and 11) will be evaluated against P53, BAX, Caspase-3, Caspase-6, Caspase-8, Caspase-9, BCL-2, MMP2, and MMP9 apoptotic markers. Furthermore, a molecular docking study will be performed to recommend the potential activity of the examined candidates to induce apoptosis as a recommended mechanism for their antitumor activity. Finally, ADMET studies will be applied to the examined candidates to investigate their physicochemical and pharmacokinetic properties.

### 1.1. Rational of design

**Fig. 2:**
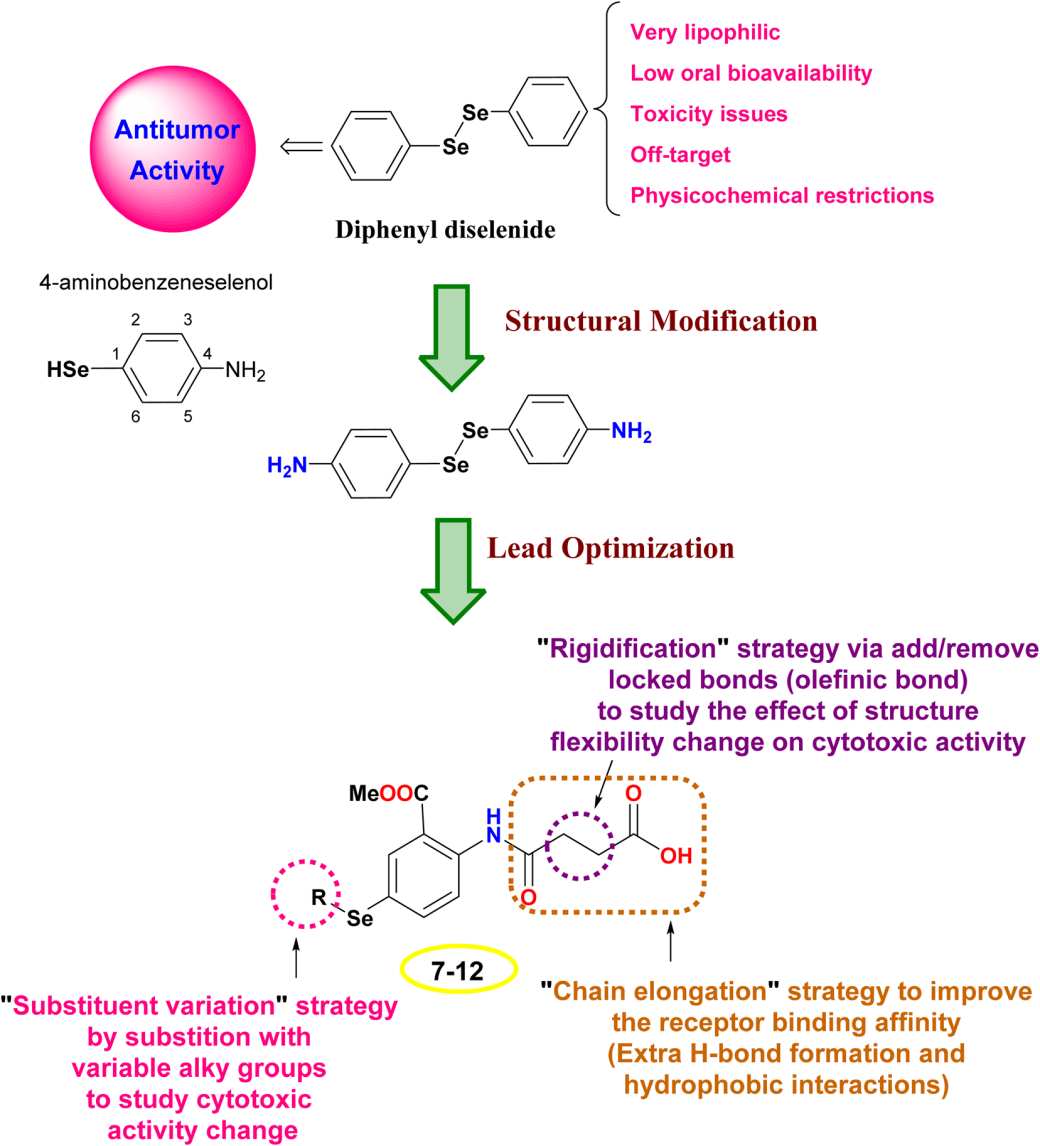
The design rationale of the investigated organoselenium compounds (7–12).

The design rationale was based on combining different lead optimization approaches to improve the cytotoxic activity. Herein, it was displayed that the start compound; diphenyl diselenide (Ph_2_Se_2_) is very lipophilic with low oral bioavailability, toxicity issues due to off-target activities, and restricted physicochemical properties.^42,43^ Therefore, Ph_2_Se_2_ was simplified to 4-aminobenzeneselenol. Accordingly, a lead optimization tool was employed using substituent variation, chain elongation, and rigidification approaches. Thus, the hydrogen atom of the –SeH functionality was substituted with different alkyl groups to pursue the cytotoxic activity change (substituent variation approach). Besides, the 4-amino group was replaced with an amido-butanoic acid motif to improve the receptor binding affinity *via* affording extra hydrophobic and H-bond interactions (chain elongation approach). Furthermore, aiming to study the effect of compound flexibility change on cytotoxic activity, block bonds (olefenic bonds) were included between α and β carbons of butanoic acid elongated chain (rigidification approach), as shown in Fig. 2.

## 2. Results and discussion

### 2.1. Chemistry

**Scheme 1:**
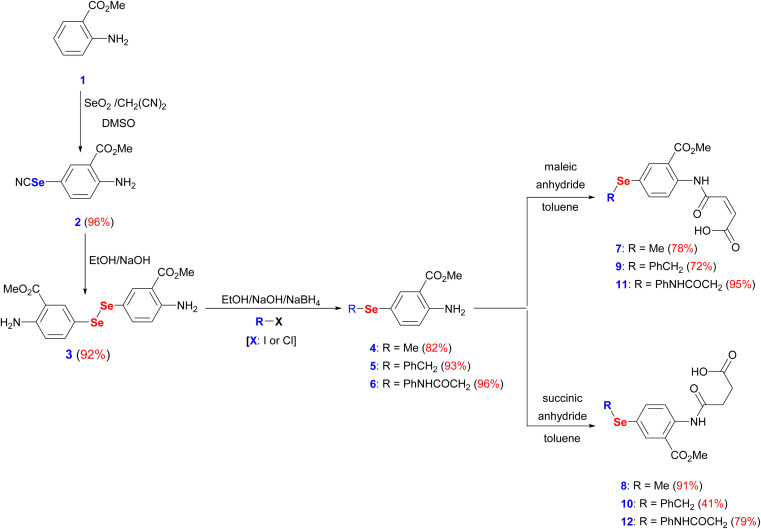
Synthesis of target amidic acids (7–12).

The interest in developing novel OSe candidates is growing stimulated by their privileged antitumor and antioxidant activities.^44–46^ Therefore, efficient, and innovative procedures are highly desired to prepare novel functionalized OSe agents and investigate their potential bioactivities. Although significant progress made in the synthesis of OSe compounds, their development has been frequently hindered by different synthetic complications.^44,47^ These challenges include the use of complicated reaction conditions (*e.g.* absence of O_2_/air or elevated temperature) as well as the hazardous, costly, sensitive, and limited functional group tolerance OSe reagents such as SeOCl_2_, KSeCN, SeCl_4_, and SeF_6_.^48–50^ Accordingly, the use of mild, and simple procedures employing stable OSe reagents compatible with a wide functional group is highly required. Diaryl diselenides are key precursors for diverse multifunctional OSe candidates such as aryl selenide halide (ArSeX) which in turn promotes selenocyclization of olefins and acetylenes to give structurally diverse selenaheterocycles.^51^ Their notable stability, safety, and ease of handling made them ideal for the optimization of new reactions.^51^ Indeed, diaryl diselenides can undergo a wide range of transformations and give access to various Se-containing reactive species such as RSe˙, RSe^−^, and RSe^+^ as well as Se-based functionalities (*e.g.* selenenic, seleninic, and selenonic acids).^52,53^ Likewise, amidic acid motifs play a fundamental role in the biological effectiveness of different biomolecules such as peptides, pseudopeptides, enzymes, and several pharmacologically active agents.^36,54^ Their exceptional activities stem from their unique electronic properties and their tendency to form hydrogen bonding. Therefore, it is hypothesized that the development of amidic acids-containing OSe scaffolds would result in enhanced antitumor activities compared to their respective amidic acids or OSe precursors alone. To this point, dimethyl 5,5′-diselanediylbis(2-aminobenzoate) (3) was proposed as the diselenide starting building block due to its diverse and reactive functional groups *i.e.* the amino and the ester groups. Furthermore, OSe 3 is prepared on a gram scale, and its high stability together with its good solubility in most organic solvents renders it a versatile synthon for various organic transformations.^36,38,39^ OSe 3 is obtained in excellent yield (92%) by the hydrolysis of selenocyanate derivative 2 using NaOH in C_2_H_5_OH at ambient temperature (Scheme 1). The reduction of the diselenide functionality in OSe 3 by NaBH_4_ led to the generation of the corresponding sodium arylselenolate. The latter is a strong and reactive nucleophile, however O_2_-sensitive, and therefore it was instantaneously trapped by the reaction with electrophiles such as iodomethane, α-chlorotoluene, and 2-chloroacetanilide to give the respective *para*-substituted primary aromatic OSe amines 4, 5, and 6 in very good yields (up to 96%) (Scheme 1). The nucleophilic attack of the amine functionality of the OSe compounds 4, 5, and 6 on the maleic and succinic anhydride carbonyl carbon resulted in ring opening and the subsequent formation of the *N*-amidic acids 7–12 in good yields (up to 95%) as shown in Scheme 1.

### 2.2. Biological evaluation

#### 2.2.1. Growth inhibition% of the investigated organoselenium compounds (7–12) against a series of cancer and normal cell lines

Eligible growth inhibition% (GI%) was experienced by the most investigated OSe compounds as shown in Table 1 using doxorubicin (Dox), as a reference anticancer drug. Dox is generally used as a positive control and widespread chemotherapeutic drug owing to its multiple modes of action (*e.g.*, topoisomerase II inhibition and intercalation into DNA).^55,56^

**Table 1:** Organoselenium compounds (7–12) GI% utilizing fifteen cancer cell lines and two normal cell lines

Comp no./cell line name	7	8	9	10	11	12	Dox
HNO97	55.60	53.62	53.37	55.83	49.98	56.83	70.50
HN9	46.02	34.13	48.93	53.72	37.82	45.89	82.03
MCF7	34.78	65.02	62.98	64.25	57.86	50.52	0.80
CaCo2	48.44	45.22	41.18	45.20	41.69	33.70	2.81
HEP2	60.04	67.71	66.25	68.87	62.91	60.78	62.10
HEPG2	67.98	72.60	72.15	62.55	57.31	54.95	73.32
A431	62.13	62.55	67.06	71.31	74.31	66.20	68.30
A375	73.95	75.79	65.54	70.60	67.89	66.58	69.38
H1299	57.88	58.32	63.14	60.39	64.79	48.88	94.96
A549	42.72	60.07	68.23	73.05	73.49	62.56	86.43
HCT_116_	79.20	77.64	79.73	68.18	53.95	72.04	71.01
PC3	30.52	70.26	37.11	19.82	32.61	50.59	89.95
FaDu	58.11	79.39	71.29	52.65	64.81	60.51	92.94
MDA-MB-468	92.05	91.94	90.95	89.39	86.74	73.61	96.02
HeLa	57.96	54.77	69.01	73.96	80.20	86.37	92.71
OEC	21.64	69.57	34.23	35.74	31.84	51.42	57.90
HSF	27.45	24.15	33.87	36.66	33.44	25.63	26.79

Intriguingly, among the investigated OSe compounds, 8 manifested the best mean GI% (64.60%) in comparison to Dox which displayed a GI% of 70.22%. Moreover, low GI% values were attained by the investigated OSe compounds against the utilized normal cell lines assuring their safety and selectivity to cancer cells.

##### 2.2.1.1 Structure–activity relationship

**Fig. 3:**
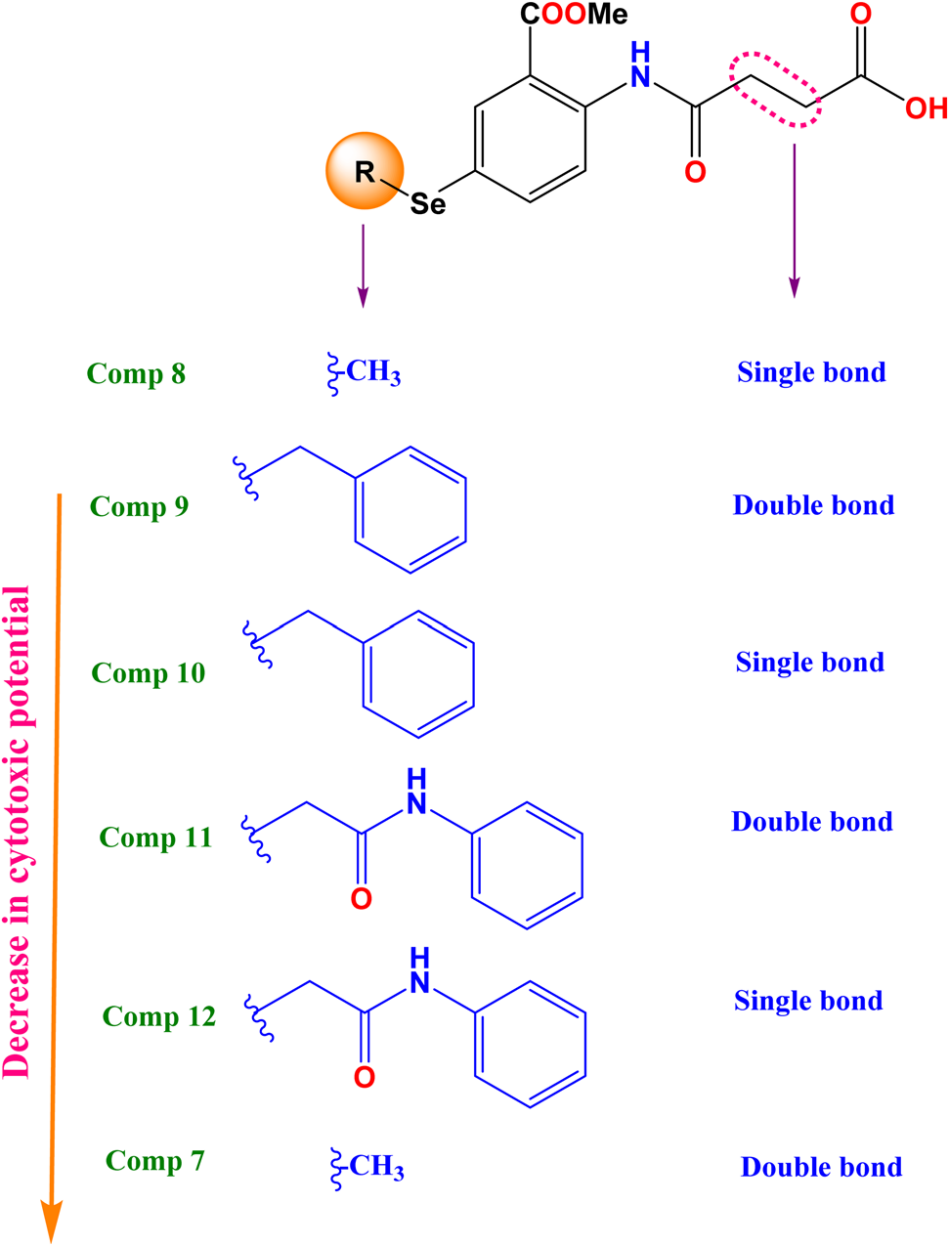
Structure cytotoxic relationship studies of the investigated compounds (7–12).

To broaden our knowledge and predict the inhibitory potential of the examined compounds as potential anticancer agents, a thorough structure–activity relationship (SAR) analysis was conducted (Fig. 3). This analysis involved deliberate modifications to the 4-((4-hydroseleno-2-(methoxycarbonyl)phenyl)amino)-4-oxobutanoic acid scaffold. Various cancer cell lines were investigated to establish the correlation between structural modifications and anticancer activity based on:

(A) The mean growth inhibition (GI)%

(a) Interestingly, it was revealed that substituting the Se with a methyl group (compound 8), displayed the highest GI% and thus the highest cytotoxic potential, as shown in Fig. 3.

(b) However, a quite decrease in GI% was noticed by substituting the Se with the benzyl group (compound 10), by substituting the Se with the benzyl group along with α, β unsaturated acid derivative (compound 9), or by substituting the Se with phenyl acetamido group along with α, β unsaturated acid derivative (compound 11).

(c) Notably, the weakest GI% was displayed by substituting the Se with methyl along with α, β unsaturated acid derivative (compound 7), or substituting the Se with phenyl acetamido group (compound 12), as depicted in Fig. 3.

#### 2.2.2. Cytotoxic inhibitory concentration 50 (IC_50_) evaluation against HCT_116_, HEPG2, A375, MDA-MB-468, and A431 cancer cell lines

**Fig. 4:**
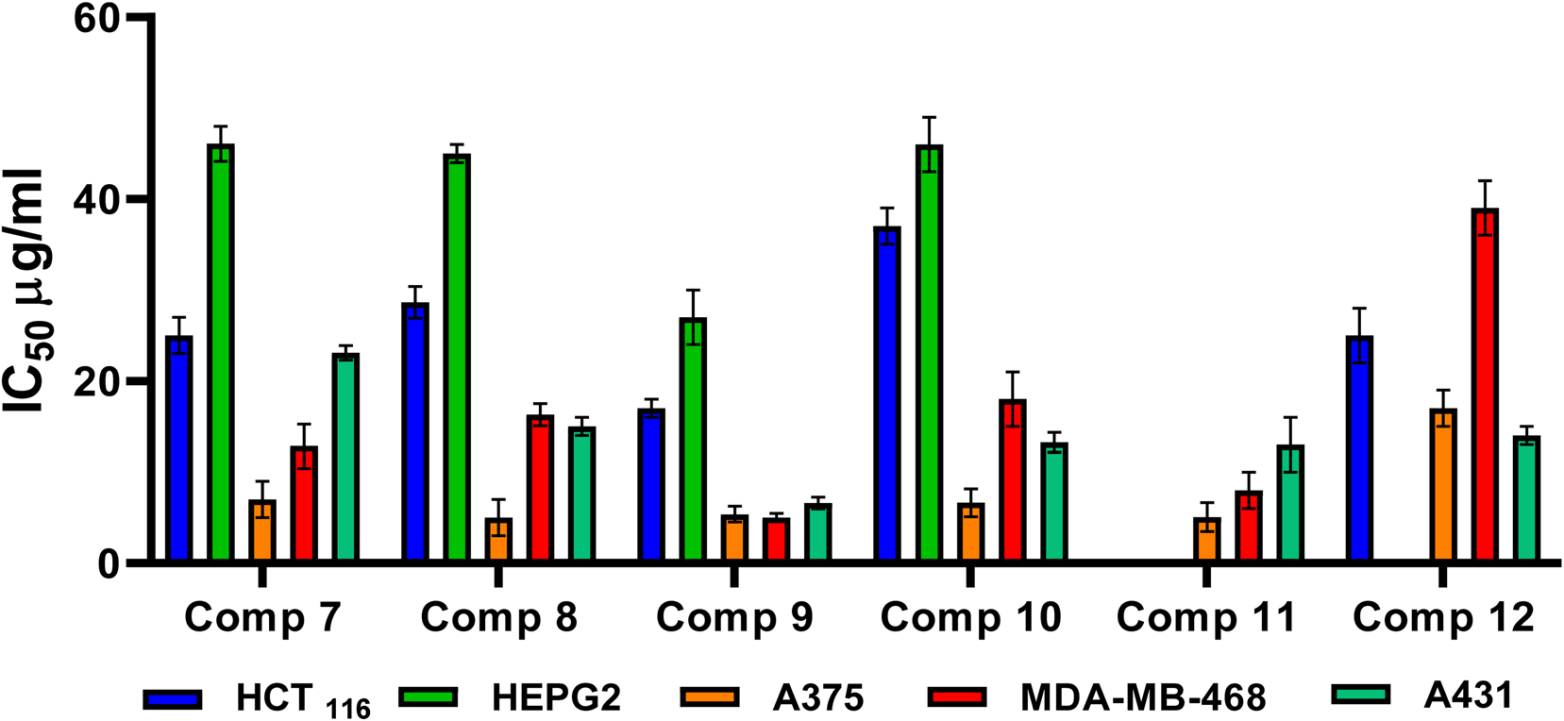
Cytotoxic inhibitory concentration 50 (IC_50_) evaluation of the assessed compounds (7–12) against HCT_116_, HEPG2, A375, MDA-MB-468, and A431 cancer cell lines.

The cytotoxic inhibitory concentration 50 (IC_50_) of the investigated compounds (7–12) was pursued against the tumor cell lines that experienced the most outstanding GI%. Hence, the cytotoxic inhibitory concentration 50 (IC_50_) on colorectal carcinoma (HCT_116_), hepatocellular carcinoma (HEPG2), human melanoma cancer (A375), human breast cancer (MDA-MB-468), and epidermoid carcinoma (A431) was investigated using the SRB assay.^57^ Among the investigated compounds, compound 9 displayed the highest cytotoxic effect against the investigated cancer cell lines, in particular, MDA-MB-468 and A431 cell lines with IC_50_ values of 5.03 and 6.6 μg mL^−1^, respectively, assuring its anticancer potential, as shown in Fig. 4.

#### 2.2.3. Protein expression of the apoptosis-related genes

Compounds 8, 9, 10, and 11 with the superior IC_50_ values especially against the A375 cancer cell line, were selected to investigate their apoptosis induction potential. The protein expression analysis for apoptosis-related genes was evaluated on the A375 cancer cell line in the presence of the target candidates (8, 9, 10, and 11). Herein, the protein expression levels of P53, BAX, caspases-3, 6, 8, and 9, BCL-2, MMP2, and MMP9 in both the treated and untreated cells were measured. This could help to obtain deeper information about the mod of action underlying the antitumor effects induced by compounds 8, 9, 10, and 11.

**Fig. 5:**
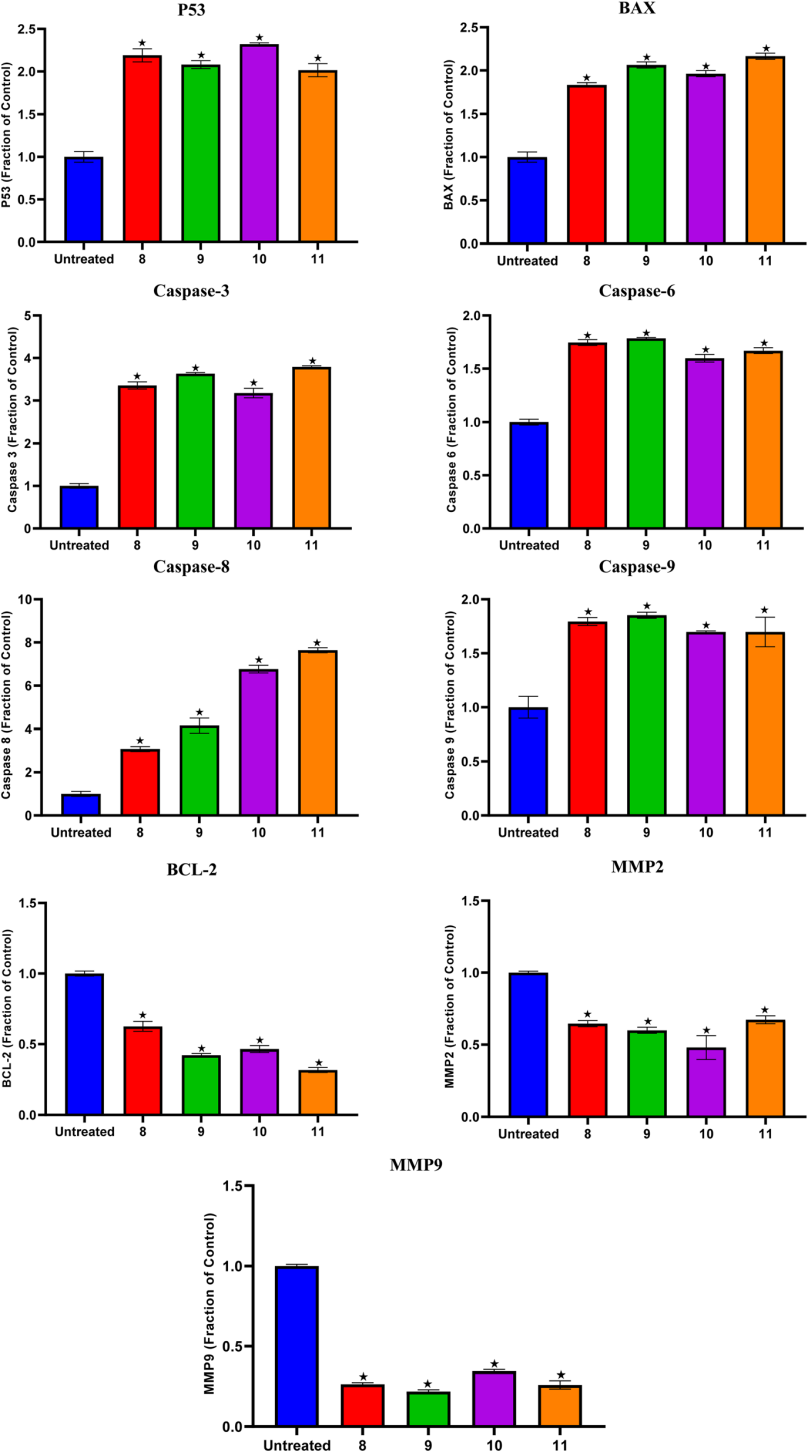
Compounds 8, 9, 10, and 11 protein expression levels for P53, BAX, Caspases-3, 6, 8, and 9, BCL-2, MMP2, and MMP9 in both the treated and untreated A375 cancer cell line.

Compounds 8, 9, 10, and 11 treatments showed the upregulation of apoptotic proteins, as presented in Fig. 5. Thus, P53, BAX, Caspases-3, 6, 8, and 9 were upregulated by (2.19, 1.83, 3.35, 1.74, 3.07, and 1.79), (2.08, 2.06, 3.63, 1.78, 4.15, and 1.85), (2.32, 1.96, 3.17, 1.59, 6.76, and 1.69), and (2.01, 2.16, 3.79, 1.66, 7.64, and 1.69)-fold changes by compounds 8, 9, 10, and 11, respectively. However, compounds 8, 9, 10, and 11 expressed the downregulation of the investigated antiapoptotic proteins. Accordingly, BCL-2, MMP2, and MMP9 were downregulated by (0.62, 0.64, and 0.26), (0.42, 0.59, and 0.21), (0.46, 0.48, and 0.34), and (0.31, 0.67, and 0.25)-fold changes by compounds 8, 9, 10, and 11, respectively, Fig. 5.

### 2.3. *In silico* studies

#### 2.3.1. Molecular docking

Molecular docking was performed to investigate the apoptotic induction potential of the target OSe-based maleanilic and succinanilic acids (7–12) through the inhibition of Caspase-6. The co-crystallized inhibitor of the target human Caspase-6 (PDB ID: 8EG6) was observed to form four hydrogen bonds with His219, Cys163 (2), and Arg220, besides, a covalent bond with Cys264. Accordingly, the amino acids are the most crucial to produce the antagonistic activity towards the Caspase-6 receptor. Moreover, the examined compounds (7–12) showed promising scores of −6.50, −6.92, −7.20, −7.02, −7.31, and −8.48 kcal mol^−1^, respectively, compared to the docked co-crystallized inhibitor of Caspase-6 (−8.20 kcal mol^−1^).

**Fig. 6:**
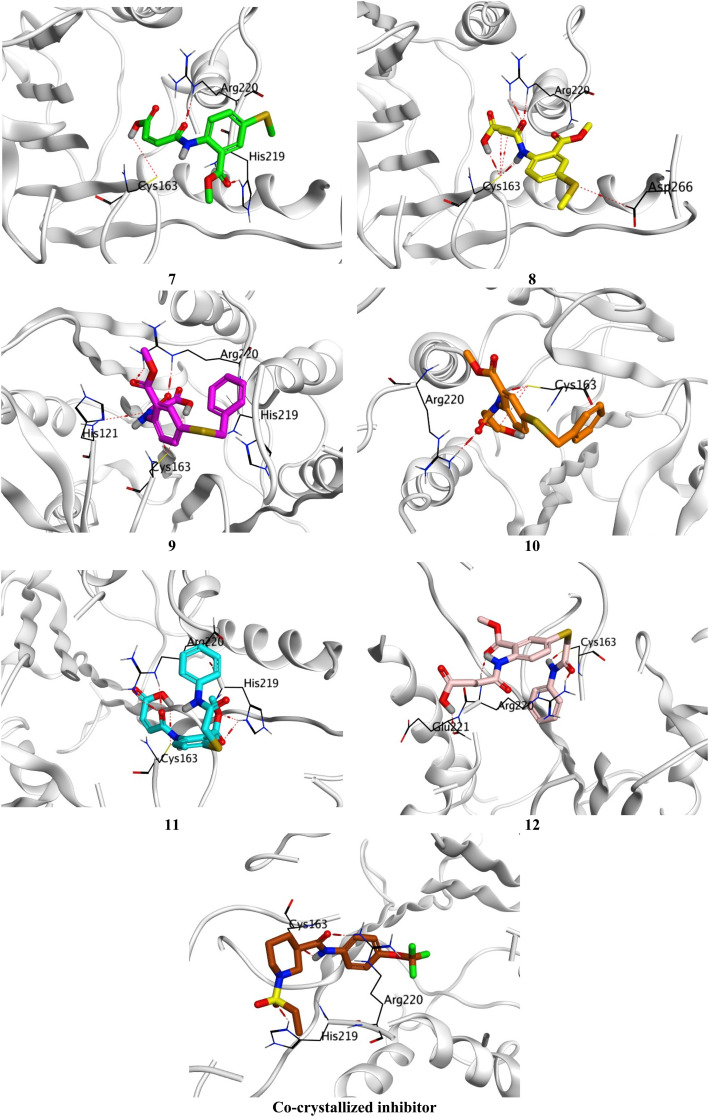
3D Binding interactions of the target candidates (7–12) within the binding site of human Caspase-6 (PDB ID: 8EG6) compared to the irreversible co-crystallized inhibitor.

Compound 7 formed three hydrogen bonds with His219, Cys163, and Arg220, however, compound 8 formed seven hydrogen bonds with Cys163 (4), Arg220 (2), and Asp266. On the other side, compound 9 showed five hydrogen bonds with Cys163 (2), Arg220 (2), and His121, and one pi-hydrogen bond with His219, respectively. Besides, compound 10 described four hydrogen bonds with Cys163 (3) and Arg220. Moreover, compound 11 represented five hydrogen bonds with His219 (2), Cys163 (2), and Arg220, and compound 12 had four hydrogen bonds with Cys163, Arg220 (2), and Glu221. Notably, the docked co-crystallized inhibitor of Caspase-6 formed four hydrogen bonds with His219, Cys163 (2), and Arg220 (Fig. 6).

Based on the previously described binding scores and interactions of the studied candidates (7–12) compared to the co-crystallized inhibitor of Caspase-6; we could confirm the superior inhibitory potential and apoptotic induction towards the Caspase-6 target.

#### 2.3.2. Physicochemical, ADME, and pharmacokinetic properties prediction

**Fig. 7:**
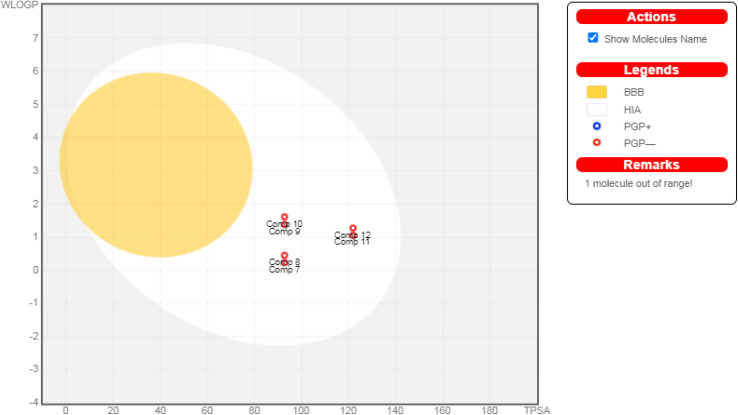
The boiled-egg diagram for all the afforded compounds (7–12) as well as doxorubicin as a reference control.

Regarding their physicochemical features, all the OSe compounds manifested feasible H_2_O solubility except for compound 9 which exhibited moderate water solubility. Since the drug's ability to dissolve at the site of absorption is crucial for its effectiveness. This characteristic can simplify the formulation process, leading to fewer absorption and thereby effectiveness issues.^58^ Additionally, all the assessed OSe compounds exhibited high GIT absorption owing to their reasonable lipophilicity. Hence, feasible bioavailabilities upon oral administration are anticipated.^59,60^ Obviously, all investigated compounds cannot pass through the blood–brain barrier, avoiding any serious CNS side effects. Fortunately, none of the assessed compounds seems to be affected by P-glycoprotein, assuring they are well absorbed from GIT, as shown in Fig. 7. Interestingly, except for compound 12, all investigated compounds do not exhibit inhibition for all common hepatic metabolizing enzymes (CYP1A2, CYP2C19, CYP2C9, CYP2D6, and CYP3A4). Moreover, based on Lipinski's rule, these compounds all have promising characteristics for good oral absorption.^61^ In addition, the bioavailability snapshot radars for the studied OSe candidates were presented in ESI Fig. S61.†

Moreover, regarding their toxicity parameters, it was shown that all assessed compounds do not exhibit Ames toxicity, assuring their eligibility is not mutagenic.^62^ Additionally, all investigated compounds are fortunately non-inhibitors of hERG I and II, so they do not exhibit a cardiotoxic effect.^63^ Notably, investigated compounds are non-hepatotoxic assuring their safety, as depicted in Table 2.

**Table 2:** The anticipated ADMET and physicochemical features of the assessed compounds (7–12) along with doxorubicin

		Comp 7	Comp 8	Comp 9	Comp 10	Comp 11	Comp 12	Dox
Molecular properties	Molar refractivity	74.20	74.67	98.68	99.16	108.03	108.51	132.66
	TPSA (Az)	92.70	92.70	92.70	92.70	121.80	121.80	206.07
	log *P*_o/w_ (WLOGP)	0.24	0.46	1.39	1.62	1.06	1.28	−0.32
	Consensus log *P*_o/w_	0.74	0.77	1.82	1.85	1.21	1.24	0.44
	Water solubility	S	S	MS	S	S	S	S
Pharmacokinetics parameters	GI absorption	High	High	High	High	High	High	Low
	BBB permeant	No	No	No	No	No	No	No
	P-gp substrate	No	No	No	No	No	No	Yes
	CYP1A2 inhibitor	No	No	No	No	No	No	No
	CYP2C19 inhibitor	No	No	No	No	No	Yes	No
	CYP2C9 inhibitor	No	No	No	No	No	No	No
	CYP2D6 inhibitor	No	No	No	No	No	No	No
	CYP3A4 inhibitor	No	No	No	No	No	No	No
Drug/Lead likeness	Drug likeness (lipinski)	Yes	Yes	Yes	Yes	Yes	Yes	No
	Lead likeness	Yes	No	No	No	No	No	No
Toxicity parameters	Ames toxicity	No	No	No	No	No	No	No
	Max. Tolerated dose (log mg kg^−1^ per day)	0.901	0.851	0.204	0.222	−0.329	−0.336	0.081
	hERG I inhibitor	No	No	No	No	No	No	No
	hERG II inhibitor	No	No	No	No	No	No	Yes
	Oral rat acute toxicity (LD_50_) (mol kg^−1^)	2.404	2.409	2.523	2.529	2.762	2.781	2.408
	Oral rat chronic toxicity (LOAEL) (log mg per kg_bw per day)	1.604	1.563	1.935	1.893	2.016	1.975	3.339
	Hepatotoxicity	No	No	Yes	Yes	Yes	No	Yes
	Minnow toxicity (log mM)	0.931	0.804	0.681	0.553	−0.029	−0.156	4.412

## 3. Conclusion

*N*-Amidic acids 7–12 were prepared in good yields (up to 95%) in a two-step synthetic procedure starting from dimethyl 5,5′-diselanediylbis(2-aminobenzoate) (3). Intriguingly, compound 8 showed the best mean GI% (64.60%) in comparison to Dox which displayed a GI% of 70.22%. On the other side, compound 9 displayed the highest cytotoxic potential against the investigated cancer cell lines, in particular, MDA-MB-468 and A431 cell lines with IC_50_ values of 5.03 and 6.6 μg mL^−1^, respectively, assuring its anticancer potential. SAR analysis described that substituting the selenium with a methyl group (compound 8) displayed the highest GI% and thus the highest cytotoxic potential. However, a quite decrease in GI% was shown by substituting the selenium with the benzyl group (compound 10), by substituting the selenium with the benzyl group along with α, β unsaturated acid derivative (compound 9), or by substituting the selenium with phenyl acetamido group along with α, β unsaturated acid derivative (compound 11). Furthermore, it was shown that compounds 8, 9, 10, and 11 could induce the upregulation of the apoptotic proteins; P53, BAX, caspases-3, 6, 8, and 9. However, they can prompt the downregulation of the anti-apoptotic proteins; BCL-2, MMP2, and MMP9. In addition, the conducted ADMET studies assured eligible physicochemical and pharmacokinetic properties of the assessed compounds. Moreover, based on the molecular docking scores and interactions towards Caspase-6; we could confirm the superior inhibitory potential and apoptotic induction.

## 4. Experimental

### 4.1. Synthesis of the amidic acid-containing OSe agents

The organic selenide-based maleanilic and succinanilic acids were prepared according to our reported literature method (see detailed experimental procedures in the ESI†).^37,64^ Details in ESI Data, SI 1.†

### 4.2. Biological evaluation

#### 4.2.1. GI% of the investigated organoselenium compounds (7–12) against a series of cancer and normal cell lines

This study used the SRB colorimetric assay to measure the GI% of the newly synthesized organoselenium compounds (7–12)^57^ in fifteen human cancer cell lines namely; human breast cancer (MCF-7 and MDA-MB-468), human melanoma cancer (A735), colorectal carcinoma (CaCo2 and HCT_116_), hepatocellular carcinoma (HEPG2), human larynx cancer (HEP2), epidermoid carcinoma (A431), human tongue carcinoma (HNO97 and HN-9), human prostate cancer (PC3), non-small cell lung cancer (H1299 and A549), and human pharynx squamous carcinoma (FaDu). Besides, to evaluate the safety of the assessed derivatives (7–12), we pursued their effects on normal oral epithelial cells (OEC) and human skin fibroblast normal cells (HSF) using established cell line assays. Details in ESI Data, SI 2.†

#### 4.2.2. Cytotoxic inhibitory concentration 50 (IC_50_) evaluation against HCT_116_, HEPG2, A375, MDA-MB-468, and A431 cancer cell lines

A range of concentrations (12.5, 25, 50, and 100 μg mL^−1^) of the investigated compounds (7–12) were tested against the cancer cell lines (ESI Fig. S62–S67†). Details in ESI Data, SI 3.†

#### 4.2.3. Protein expression of the apoptosis-related genes

Compounds 8, 9, 10, and 11 with the superior IC_50_ values especially against the A375 cancer cell line, were selected to investigate their apoptosis induction potential. Where, the protein expression analysis for apoptosis-related genes was evaluated on the A375 cancer cell line in the presence of the target candidates (8, 9, 10, and 11). The protein expression levels of P53, BAX, Caspases 3, 6, 8, and 9, BCL-2, MMP2, and MMP9 in both the treated and untreated cells were measured (details in ESI Data, SI 4†). This could help to gain insights into the molecular mechanisms responsible for the antitumor effects induced by compounds 8, 9, 10, and 11.

### 4.3. *In silico* studies

#### 4.3.1. Molecular docking

The examined organic selenide-based maleanilic and succinanilic acids (7–12) were subjected to a molecular docking study using the AutoDock Vine and PyMol software.^65,66^ This was applied to investigate the apoptotic induction potential of the target members (7–12) through the inhibition of the human Caspase-6 (PDB ID: 8EG6). The organic selenides (7–12) were sketched in ChemDraw and prepared for docking by energy minimization and partial charges optimization.^67^ Human Caspase-6 (PDB ID: 8EG6, resolution: 1.82 Å) was extracted from the Protein Data Bank and prepared for docking by correction, energy minimization, and 3D hydrogenation.^68^ At the end of the molecular docking process; the best pose for each compound (based on the score and binding interactions) was isolated and visualized to be compared to that of the co-crystallized inhibitor.^69^

#### 4.3.2. Physicochemical, ADME, and pharmacokinetic properties prediction

Predicting pharmacokinetic, physicochemical, and toxicity parameters is a critical step following the synthesis of new drug candidates (molecular entities).^70–72^ To evaluate the drug's pharmacokinetics and physicochemical features, we utilized the freely available SwissADME web application from the Swiss Institute of Bioinformatics (SIB). SwissADME allowed us to predict the compounds' pharmacokinetic properties and ADME parameters, and even estimate their physical and chemical properties. To achieve this, we submitted the SMILES notation, a string representation of each compound's structure, to the SwissADME online server for calculations.^73^ In addition to SwissADME, we employed the pkCSM descriptors algorithm protocol to predict the toxicity profiles of the assessed compounds (7–12).^74^

## Data availability

The raw/processed data generated in this work are available upon request from the corresponding author.

## Author contributions

Conceptualization and supervision: Saad Shaaban and Ahmed A. Al-Karmalawy; data curation and visualization: Saad Shaaban, Ayman Abo Elmaaty, and Ahmed A. Al-Karmalawy; methodology: Saad Shaaban, Hanan A. Althikrallah, Ayman Abo Elmaaty, Amr Negm, and Ahmed A. Al-Karmalawy; writing – review & editing: Saad Shaaban, Ayman Abo Elmaaty, and Ahmed A. Al-Karmalawy. Finally, all authors revised and approved the final submitted version of the manuscript.

## Conflicts of interest

The authors declare no conflict of interest.

## Funding

The research work was funded by the Deputyship for Research and Innovation, Ministry of Education in Saudi Arabia (Project number INST220).

## Supplementary Material

RA-014-D4RA02944E-s001
