# Supplementary material for: Repurposed organoselenium tethered amidic acids as apoptosis inducers in melanoma cancer via P53, BAX, caspases-3, 6, 8, 9, BCL-2, MMP2, and MMP9 modulations
Source: RSC Adv. 2024 Jun 10;14(26):18576–87. doi: 10.1039/d4ra02944e (PMC11164031; doi:10.1039/d4ra02944e)
Supplement: RA-014-D4RA02944E-s001 [file RA-014-D4RA02944E-s001.pdf]

Electronic Supplementary Material (ESI) for RSC Advances.
This journal is © The Royal Society of Chemistry 2024

Supporting Information

Repurposed Organoselenium Tethered Amidic Acids as Apoptosis Inducers in Melanoma Cancer *via* P53, BAX, Caspases-3, 6, 8, 9, BCL-2, MMP2, and MMP9 Modulations

Saad Shaaban[1,2,*], Hanan A. Althikrallah[1], Amr Negm[1], Ayman Abo Elmaaty[3], Ahmed A. Al-Karmalawy[4,5*]

[1] Department of Chemistry, College of Science, King Faisal University, Al-Ahsa 31982, Saudi Arabia.

[2] Department of Chemistry, Faculty of Science, Mansoura University, 35516 Mansoura, Egypt.

[3] Medicinal Chemistry Department, Faculty of Pharmacy, Port Said University, Port Said, 42511, Egypt.

[4] Department of Pharmaceutical Chemistry, Faculty of Pharmacy, Horus University–Egypt, New Damietta 34518, Egypt.

[5] Pharmaceutical Chemistry Department, Faculty of Pharmacy, Ahram Canadian University, 6th of October City, Giza 12566, Egypt.

Corresponding authors:

Ahmed A. Al-Karmalawy: akarmalawy@horus.edu.eg, ORCID: https://orcid.org/0000-0002-8173-6073

Saad Shaaban: sibrahim@kfu.edu.sa, ORCID: https://orcid.org/0000-0002-7835-6926

Supporting Information

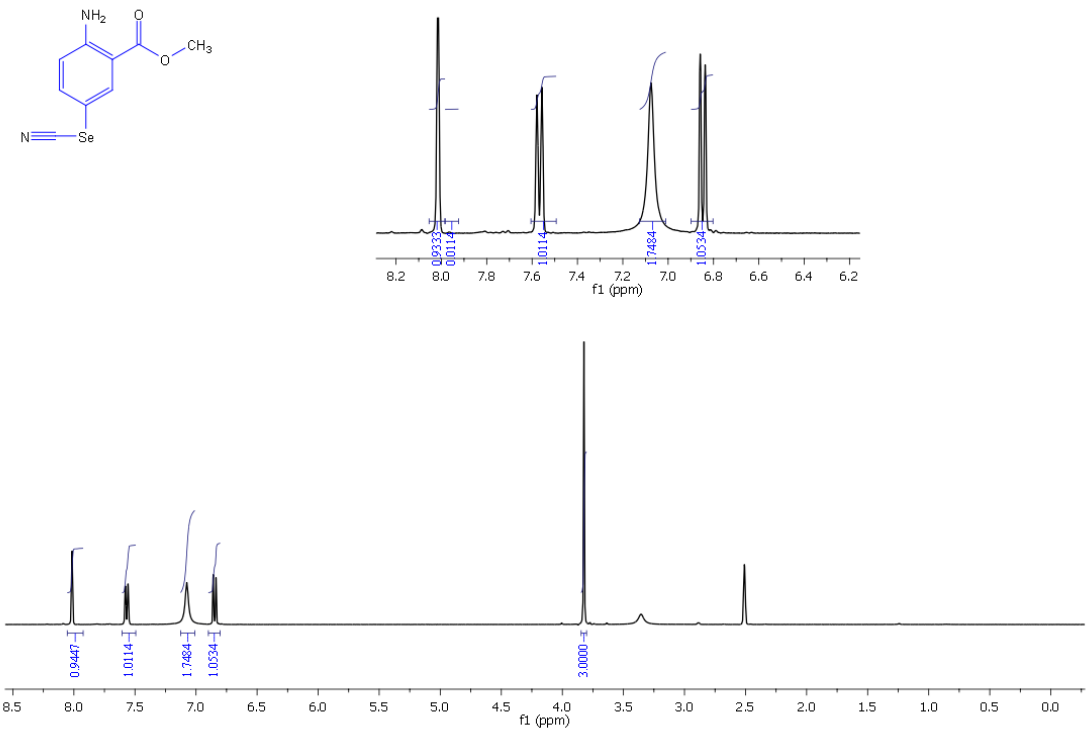

Figure S1. ^{1}H NMR chart of compound **2**.

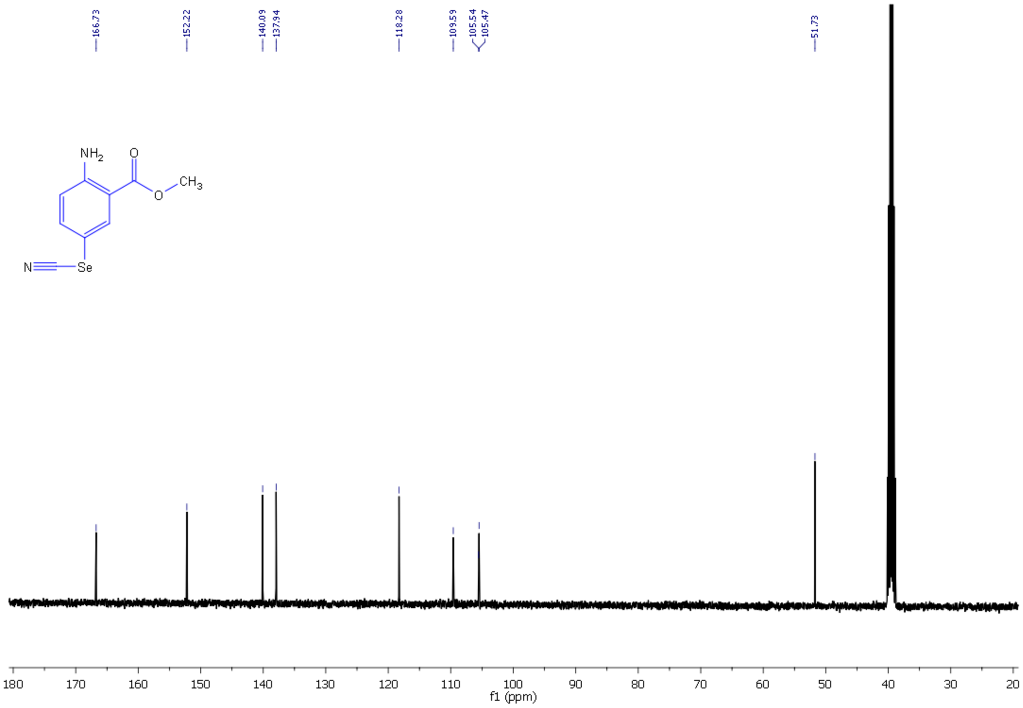

Figure S2. ^{13}C NMR chart of compound **2**.

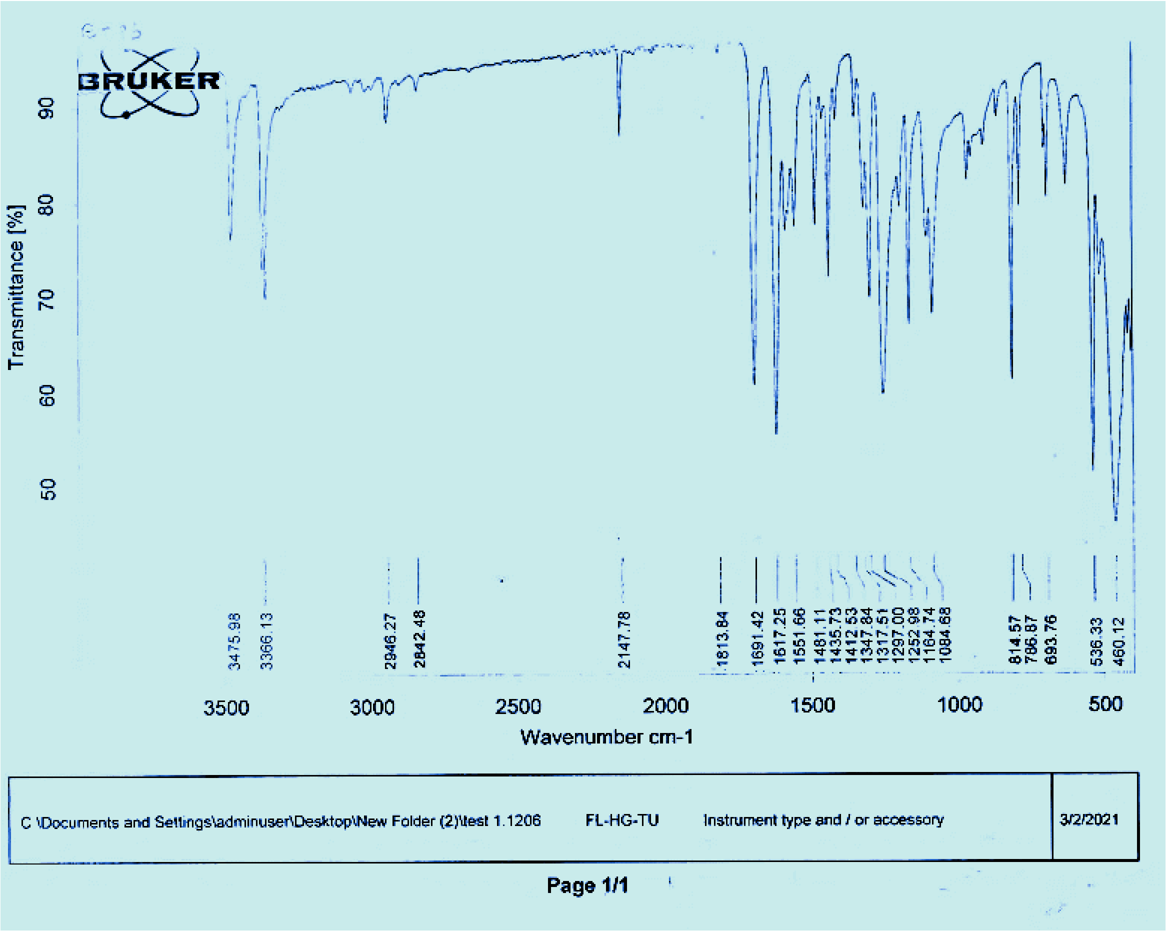

Figure S3. IR chart of compound **2**.

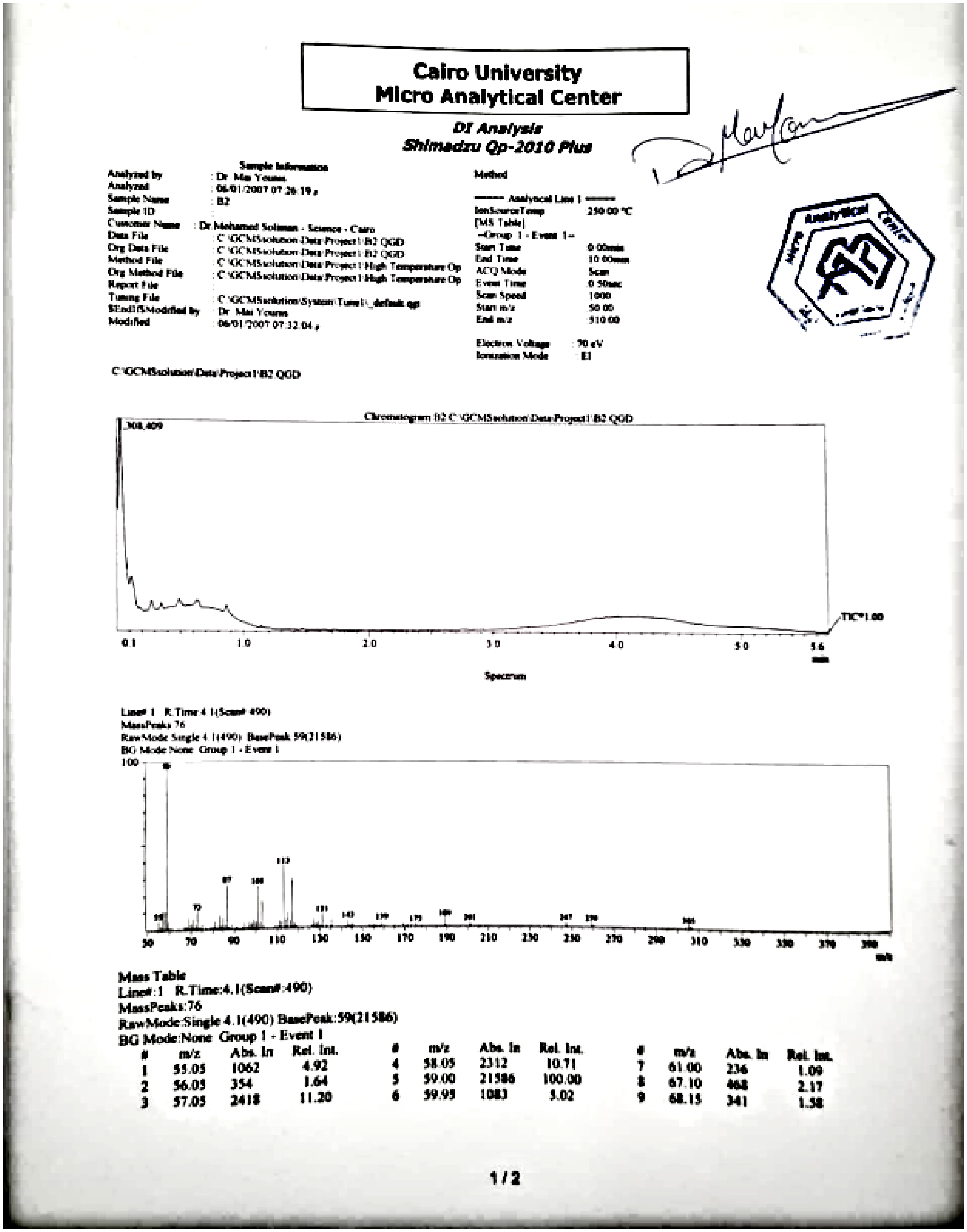

Cairo University
Micro Analytical Center

DI Analysis
Shimadzu Qp-2010 Plus

Sample Information

Analyzed by : Dr Mai Younes
Analyzed : 06/01/2007 07:26:19
Sample Name : B2
Sample ID :
Customer Name : Dr Mohamed Soliman - Science - Cairo
Data File : C:\GCMSsolution\Data\Project1\B2.QGD
Org Data File : C:\GCMSsolution\Data\Project1\B2.QGD
Method File : C:\GCMSsolution\Data\Project1\High Temperature Qp
Org Method File : C:\GCMSsolution\Data\Project1\High Temperature Qp
Report File :
Tuning File : C:\GCMSsolution\System\Tune1_default.qgt
$End1$Modified by : Dr Mai Younes
Modified : 06/01/2007 07:32:04

Method

------ Analytical Line 1 ------
IonSourceTemp 250.00 °C
[MS Table]
--Group 1 - Event 1--
Start Time 0.00min
End Time 10.00min
ACQ Mode Scan
Event Time 0.50sec
Scan Speed 1000
Start m/z 50.00
End m/z 510.00

Electron Voltage : 70 eV
Ionization Mode : EI

C:\GCMSsolution\Data\Project1\B2.QGD

Mass Table
Line#:1 R.Time:4.1(Scan#:490)
MassPeaks:76
RawMode:Single 4.1(490) BasePeak:59(21586)
BG Mode:None Group 1 - Event 1

#	m/z	Abs. In	Rel. Int.	#	m/z	Abs. In	Rel. Int.	#	m/z	Abs. In	Rel. Int.
1	55.05	1062	4.92	4	58.05	2312	10.71	7	61.00	236	1.09
2	56.05	354	1.64	5	59.00	21586	100.00	8	67.10	468	2.17
3	57.05	2418	11.20	6	59.95	1083	5.02	9	68.15	341	1.58

1/2

Figure S4. Mass chart of compound **2**.

#	m/z	Abs. In	Rel. Int.
10	69.05	1515	7.02
11	70.10	633	2.93
12	71.05	1365	6.32
13	72.05	440	2.04
14	73.00	2258	10.46
15	74.00	302	1.40
16	75.05	583	2.70
17	77.00	268	1.24
18	79.00	223	1.03
19	80.10	324	1.50
20	81.10	954	4.42
21	82.15	315	1.46
22	83.10	1628	7.54
23	84.10	481	2.23
24	85.05	1397	6.47
25	86.05	492	2.28
26	87.05	5752	26.65
27	88.00	314	1.45
28	89.05	560	2.59
29	91.10	327	1.51
30	94.10	202	0.94
31	95.10	807	3.74
32	96.10	278	1.29
33	97.10	945	4.38
34	98.15	599	2.77
35	99.10	1268	5.87
36	100.15	840	3.89
37	101.10	5503	25.49
38	102.15	699	3.24
39	103.10	3649	16.90
40	104.10	322	1.49
41	109.10	401	1.86
42	110.10	220	1.02
43	111.10	969	4.49
44	112.15	807	3.74
45	113.10	8084	37.45
46	114.10	1199	5.55
47	115.10	2001	9.27
48	116.25	936	4.34
49	117.15	6264	29.02
50	118.15	607	2.81
51	119.20	271	1.26
52	126.15	310	1.44
53	127.10	1102	5.11
54	128.15	589	2.73
55	129.10	740	3.43
56	130.15	304	1.41
57	131.10	1722	7.98
58	133.15	432	2.00
59	135.20	1132	5.24
60	143.10	908	4.21
61	144.10	210	0.97
62	145.20	492	2.28
63	153.20	234	1.08
64	155.20	281	1.30
65	159.15	662	3.07
66	169.20	334	1.55
67	171.20	204	0.95
68	173.20	202	0.94
69	175.20	346	1.60
70	189.20	1038	4.81
71	201.15	502	2.33
72	247.20	575	2.66
73	259.35	515	2.39
74	260.40	207	0.96
75	305.40	401	1.86
76	306.40	281	1.30

Figure S5. Mass chart of compound **2**.

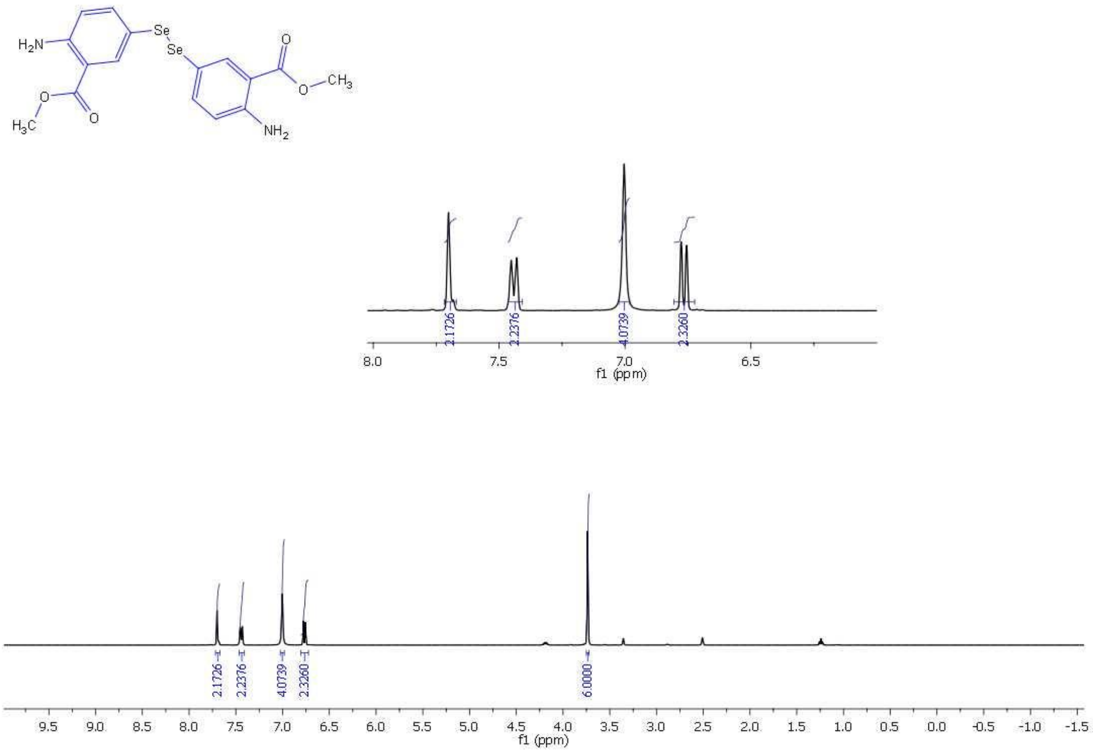

Figure S6. ^{1}H NMR chart of compound **3**.

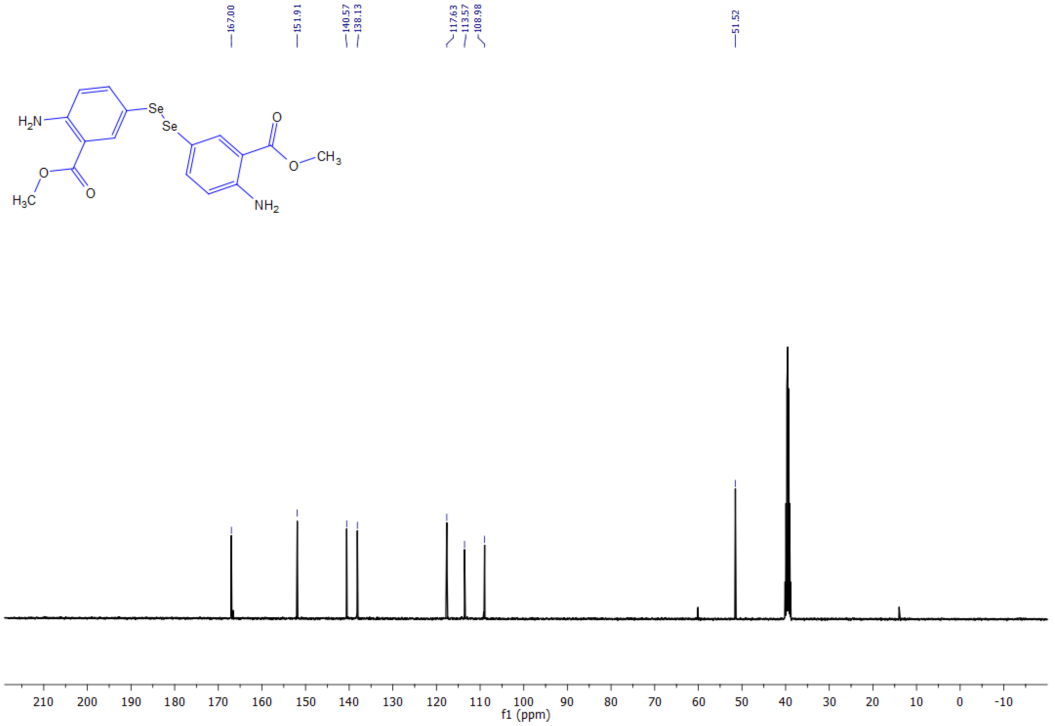

Figure S7. ^{13}C NMR chart of compound **3**.

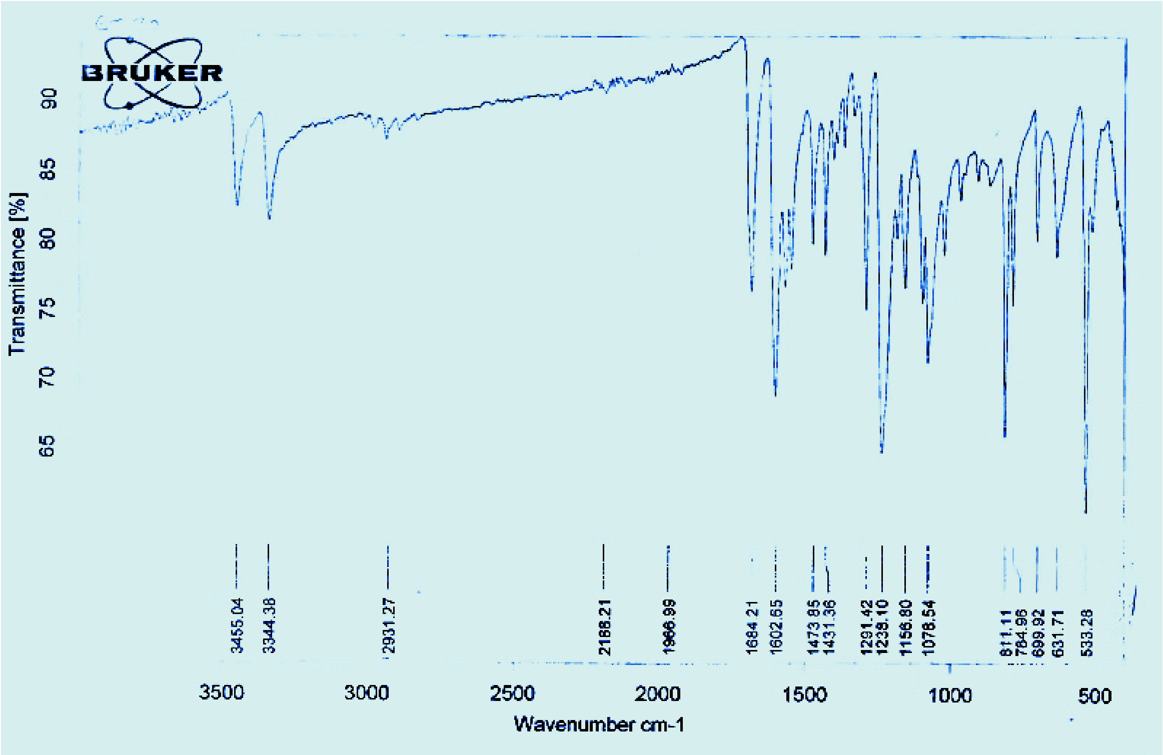

Figure S8. IR chart of compound **3**.

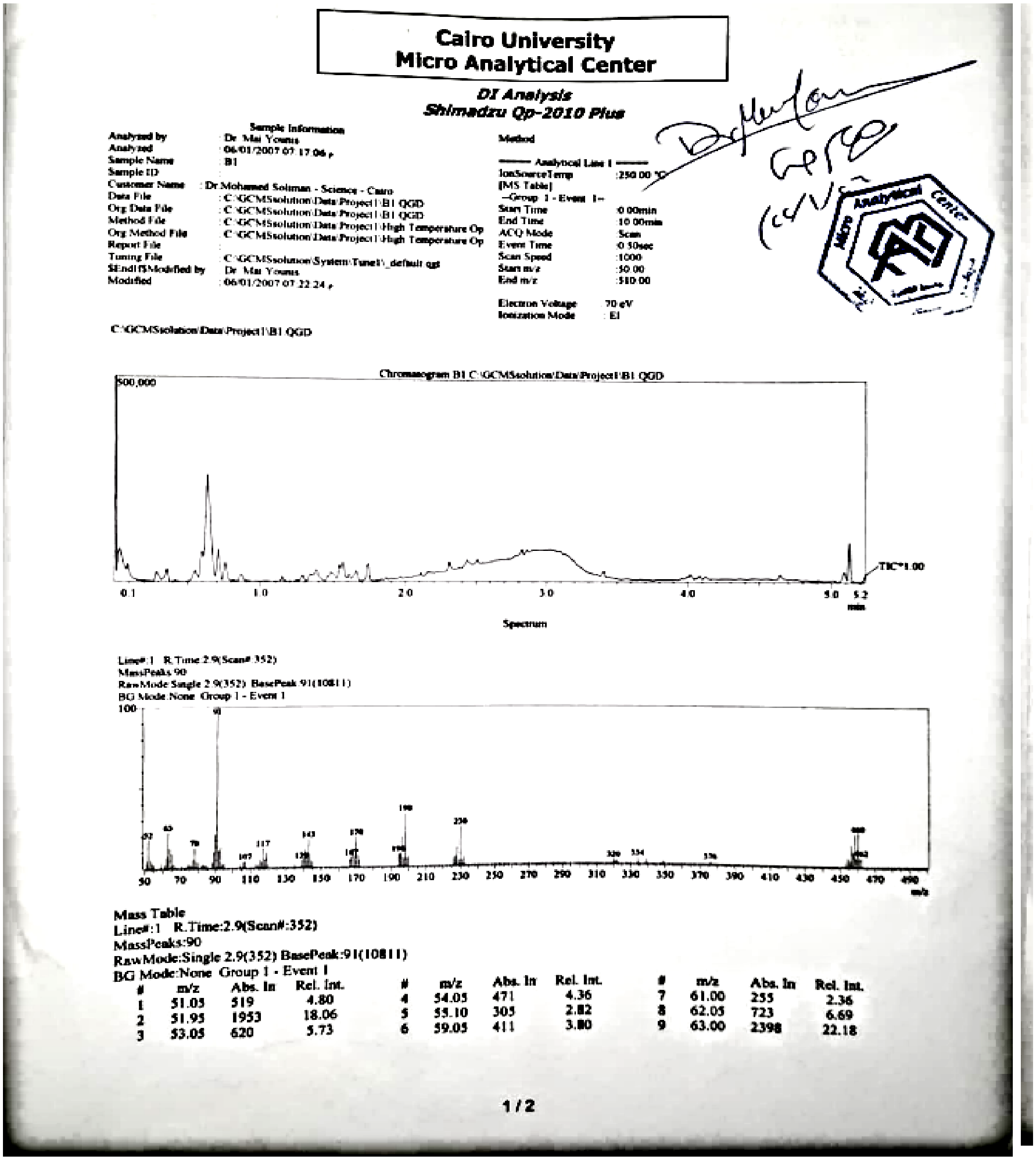

Cairo University
Micro Analytical Center

DI Analysis
Shimadzu Qp-2010 Plus

Sample Information

Analyzed by	: Dr. Mai Younis
Analyzed	: 06/01/2007 07:17:06 م
Sample Name	: B1
Sample ID	:
Customer Name	: Dr Mohamed Soliman - Science - Cairo
Data File	: C:\GCMSsolution\Data\Project1\B1.QGD
Org Data File	: C:\GCMSsolution\Data\Project1\B1.QGD
Method File	: C:\GCMSsolution\Data\Project1\High Temperature Op
Org Method File	: C:\GCMSsolution\Data\Project1\High Temperature Op
Report File	:
Tuning File	: C:\GCMSsolution\System\Tune1_default.qgt
$End1f$Modified by	: Dr. Mai Younis
Modified	: 06/01/2007 07:22:24 م

Method

—— Analytical Line 1 ——

IonSourceTemp	:250.00 °C
[MS Table]	
--Group 1 - Event 1--	
Start Time	:0.00min
End Time	:10.00min
ACQ Mode	:Scan
Event Time	:0.50sec
Scan Speed	:1000
Start m/z	:50.00
End m/z	:510.00
Electron Voltage	:70 eV
Ionization Mode	: EI

C:\GCMSsolution\Data\Project1\B1.QGD

Chromatogram B1 C:\GCMSsolution\Data\Project1\B1.QGD

Spectrum

Line#:1 R.Time:2.9(Scan#:352)
MassPeaks:90
RawMode:Single 2.9(352) BasePeak:91(10811)
BG Mode:None Group 1 - Event 1

Mass Table
Line#:1 R.Time:2.9(Scan#:352)
MassPeaks:90
RawMode:Single 2.9(352) BasePeak:91(10811)
BG Mode:None Group 1 - Event 1

#	m/z	Abs. In	Rel. Int.	#	m/z	Abs. In	Rel. Int.	#	m/z	Abs. In	Rel. Int.
1	51.05	519	4.80	4	54.05	471	4.36	7	61.00	255	2.36
2	51.95	1953	18.06	5	55.10	305	2.82	8	62.05	723	6.69
3	53.05	620	5.73	6	59.05	411	3.80	9	63.00	2398	22.18

1/2

Figure S9. Mass chart of compound **3**.

#	m/z	Abs. In	Rel. Int.	#	m/z	Abs. In	Rel. Int.	#	m/z	Abs. In	Rel. Int.
10	64.05	1311	12.13	37	113.00	308	2.85	64	198.00	3602	33.32
11	65.05	960	8.88	38	113.90	222	2.05	65	199.00	591	5.47
12	66.00	278	2.57	39	114.90	636	5.88	66	200.05	690	6.38
13	67.00	247	2.28	40	116.05	385	3.56	67	226.05	505	4.67
14	71.00	202	1.87	41	117.00	1319	12.20	68	227.05	655	6.06
15	74.00	313	2.90	42	118.05	599	5.54	69	228.00	1222	11.30
16	75.00	255	2.36	43	119.10	1022	9.45	70	229.15	316	2.92
17	76.00	214	1.98	44	120.10	310	2.87	71	230.05	2640	24.42
18	77.05	571	5.28	45	135.10	289	2.67	72	231.05	347	3.21
19	78.05	1366	12.64	46	139.00	482	4.46	73	232.05	594	5.49
20	79.10	466	4.31	47	140.00	614	5.68	74	320.00	290	2.68
21	80.10	388	3.59	48	141.05	1196	11.06	75	321.00	222	2.05
22	82.10	233	2.16	49	142.05	619	5.73	76	322.00	228	2.11
23	83.10	260	2.40	50	143.00	1894	17.52	77	334.00	398	3.68
24	84.10	220	2.03	51	144.00	850	7.86	78	339.00	318	2.94
25	85.10	278	2.57	52	144.95	409	3.78	79	349.00	230	2.13
26	88.05	377	3.49	53	166.05	493	4.56	80	376.00	215	1.99
27	89.05	1081	10.00	54	167.05	639	5.91	81	453.00	263	2.43
28	90.05	2287	21.15	55	168.05	1148	10.62	82	454.20	604	5.59
29	91.05	10811	100.00	56	169.15	688	6.36	83	455.20	425	3.93
30	92.05	1150	10.64	57	170.05	2031	18.79	84	456.25	1479	13.68
31	92.95	1429	13.22	58	171.10	802	7.42	85	457.20	1015	9.39
32	94.00	254	2.35	59	172.10	420	3.88	86	458.20	2169	20.06
33	95.00	354	3.27	60	194.05	863	7.98	87	459.15	562	5.20
34	104.00	249	2.30	61	195.05	858	7.94	88	460.15	2244	20.76
35	106.10	398	3.68	62	196.00	1993	18.43	89	461.15	521	4.82
36	107.00	444	4.11	63	197.05	575	5.32	90	462.10	618	5.72

Figure S10. Mass chart of compound **3**.

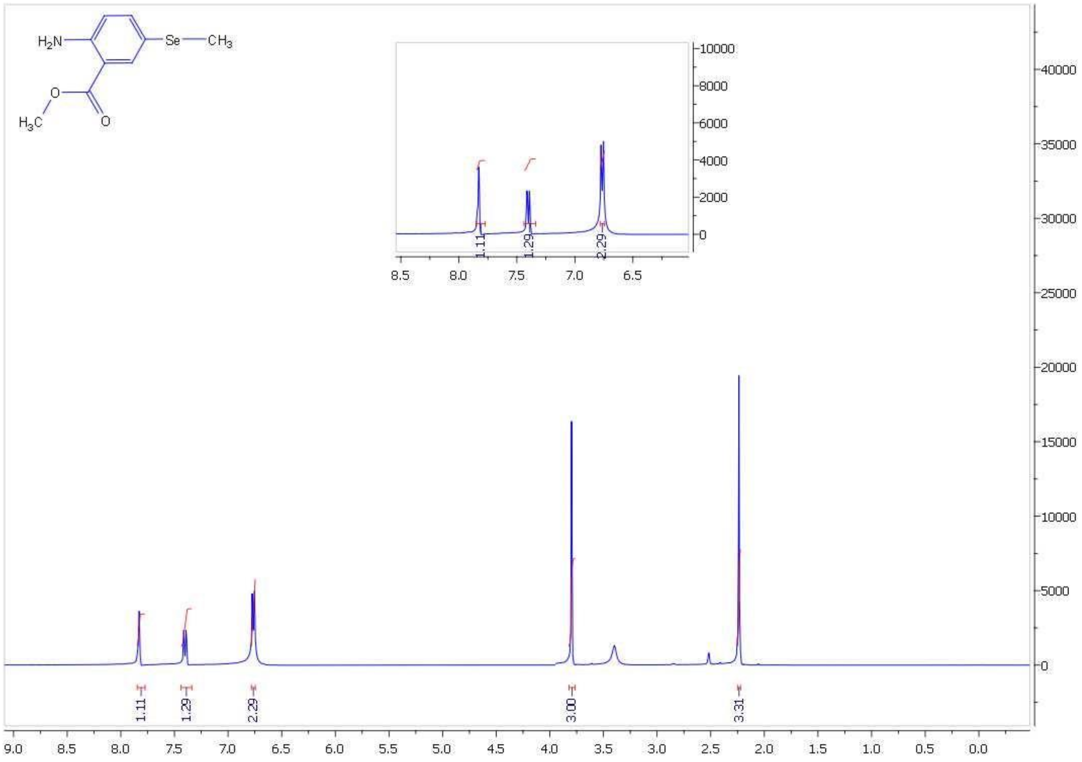

Figure S11. ^{1}H NMR chart of compound **4**.

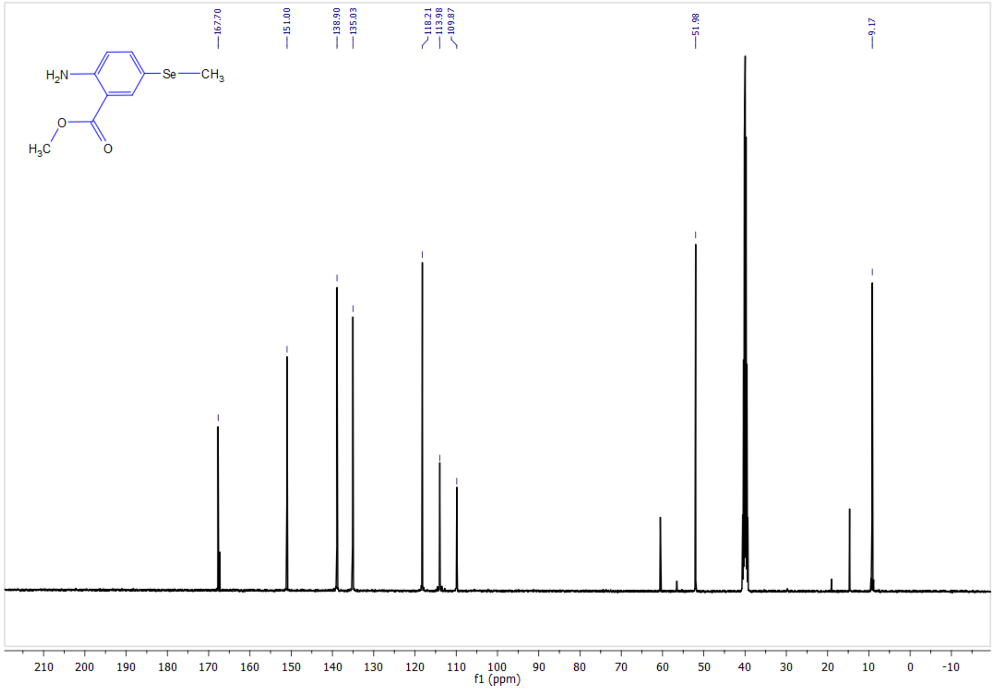

Figure S12. ^{13}C NMR chart of compound **4**.

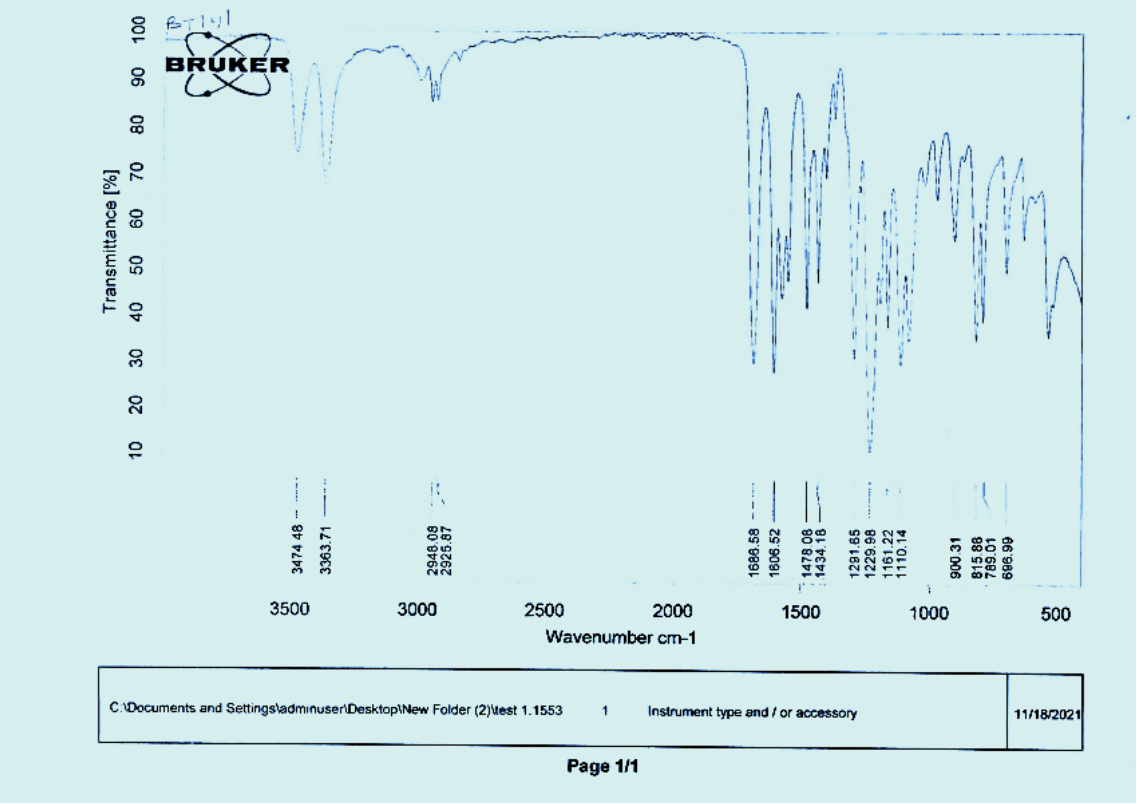

Figure S13. IR chart of compound **4**.

Cairo University
Micro Analytical Center

DI Analysis
Shimadzu Qp-2010 Plus

Sample Information

Analyzed by	: Dr. Mai Younis
Analyzed	: 16/01/2007 03:02:11 ص
Sample Name	: B35
Sample ID	:
Customer Name	: Dr Mohamed Soliman - Science - Cairo
Data File	: C:\GCMSsolution\Data\Project1\B35.QGD
Org Data File	: C:\GCMSsolution\Data\Project1\B35.QGD
Method File	: C:\GCMSsolution\Data\Project1\High Temperature Op
Org Method File	: C:\GCMSsolution\Data\Project1\High Temperature Op
Report File	:
Tuning File	: C:\GCMSsolution\System\Tune1_default.qgt
$EndIf$Modified by	: Dr. Mai Younis
Modified	: 16/01/2007 03:12:11 ص

Method

—— Analytical Line 1 ——

IonSourceTemp	:250.00 °C
[MS Table]	
--Group 1 - Event 1--	
Start Time	:0.00min
End Time	:10.00min
ACQ Mode	:Scan
Event Time	:0.50sec
Scan Speed	:1000
Start m/z	:50.00
End m/z	:310.00
Electron Voltage	: 70 eV
Ionization Mode	: EI

C:\GCMSsolution\Data\Project1\B35.QGD

Chromatogram B35 C:\GCMSsolution\Data\Project1\B35.QGD

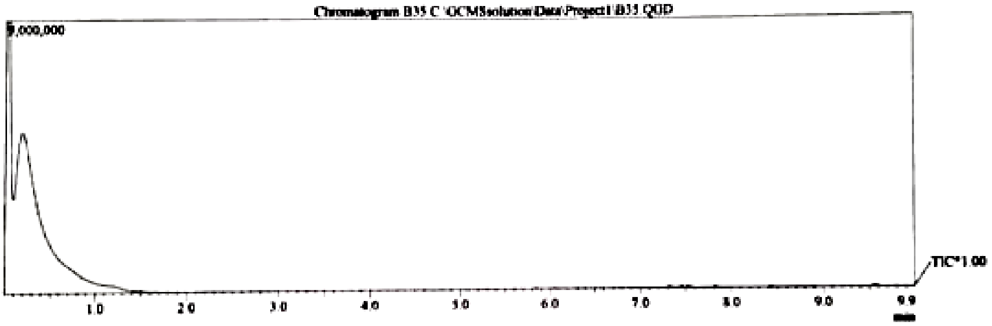

Spectrum

Line# 1 R.Time 0.2(Scan# 26)
MassPeaks 205
RawMode Single 0.2(26) BasePeak 245(391154)
BG Mode None Group 1 - Event 1

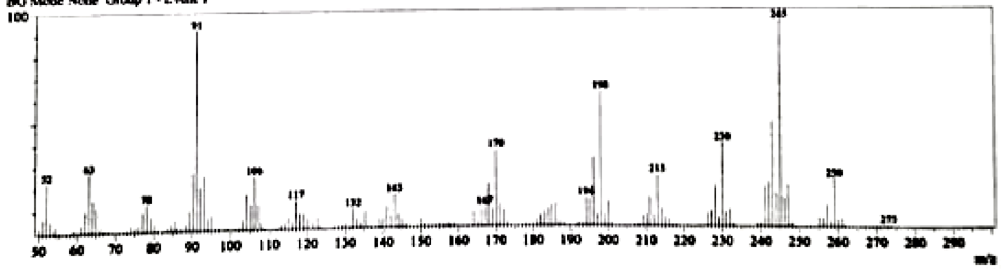

Mass Table
Line#:1 R.Time:0.2(Scan#:26)
MassPeaks:205
RawMode:Single 0.2(26) BasePeak:245(391154)
BG Mode:None Group 1 - Event 1

#	m/z	Abs. In	Rel. Int.	#	m/z	Abs. In	Rel. Int.	#	m/z	Abs. In	Rel. Int.
1	50.05	10411	2.66	4	53.00	21482	5.49	7	56.05	488	0.12
2	51.05	26260	6.71	5	54.05	11184	2.86	8	57.05	709	0.18
3	52.00	89298	22.83	6	55.00	2246	0.57	9	58.05	1164	0.30

1 / 2

Supporting Information

Figure S14. Mass chart of compound **4**.

#	m/z	Abs. In	Rel. Int.
10	59.00	6150	1.57
11	60.05	1575	0.40
12	61.05	14415	3.69
13	62.05	39733	10.16
14	63.05	103836	26.55
15	64.05	57318	14.65
16	65.05	43819	11.20
17	66.10	9838	2.52
18	67.05	4572	1.17
19	68.05	2933	0.75
20	69.10	1041	0.27
21	70.05	837	0.21
22	71.05	1646	0.42
23	72.05	1669	0.43
24	72.95	2952	0.75
25	74.05	12141	3.10
26	75.10	10198	2.61
27	76.10	12385	3.17
28	77.10	36498	9.33
29	78.05	47310	12.09
30	79.10	24441	6.25
31	80.00	15313	3.91
32	81.00	4794	1.23
33	82.55	1873	0.48
34	83.55	6981	1.78
35	84.55	13095	3.35
36	85.50	19868	5.08
37	86.10	5970	1.53
38	87.05	4792	1.23
39	88.10	12948	3.31
40	89.10	37161	9.50
41	90.15	101589	25.97
42	91.10	358905	91.76
43	92.05	75198	19.22
44	93.00	97377	24.89
45	94.00	22561	5.77
46	95.00	26670	6.82
47	96.00	4114	1.05
48	97.05	4225	1.08
49	98.35	2449	0.63
50	99.30	3386	0.87
51	100.25	1374	0.35
52	101.05	1757	0.45
53	102.10	5450	1.39
54	103.15	15671	4.01
55	104.15	62814	16.06
56	105.25	45718	11.69
57	106.20	94842	24.25
58	107.15	45188	11.55
59	108.15	10687	2.73
60	109.05	2089	0.53
61	110.05	710	0.18
62	111.05	1314	0.34
63	112.05	1199	0.31
64	113.00	7610	1.95
65	114.00	10354	2.65
66	115.00	21430	5.48
67	116.05	12818	3.28
68	117.05	50031	12.79
69	118.05	27671	7.07
70	119.10	26692	6.82
71	120.10	18391	4.70
72	121.55	12099	3.09
73	122.60	19750	5.05
74	123.55	4338	1.11
75	125.05	1061	0.27

#	m/z	Abs. In	Rel. Int.
76	126.05	2179	0.56
77	127.05	3204	0.82
78	128.00	4023	1.03
79	129.05	5617	1.44
80	130.00	7762	1.98
81	131.15	4478	1.14
82	132.10	33902	8.67
83	133.10	15628	4.00
84	134.15	8746	2.24
85	135.10	31116	7.95
86	136.15	5476	1.40
87	137.00	2783	0.71
88	138.05	3352	0.86
89	139.00	15473	3.96
90	140.00	17660	4.51
91	141.00	39022	9.98
92	142.05	21311	5.45
93	143.00	58711	15.01
94	144.00	25175	6.44
95	145.00	13801	3.53
96	146.00	5042	1.29
97	147.00	1039	0.27
98	148.10	804	0.21
99	149.15	1515	0.39
100	150.10	13154	3.36
101	151.10	4439	1.13
102	151.95	1797	0.46
103	153.00	3161	0.81
104	154.00	3514	0.90
105	155.00	5122	1.31
106	156.00	4514	1.15
107	157.05	6492	1.66
108	158.00	3975	1.02
109	159.00	5625	1.44
110	160.00	1470	0.38
111	161.00	1084	0.28
112	162.00	380	0.10
113	163.15	1307	0.33
114	164.10	28951	7.40
115	165.05	9689	2.48
116	166.00	30183	7.72
117	167.05	33926	8.67
118	168.00	75583	19.32
119	169.05	30276	7.74
120	170.00	137220	35.08
121	171.00	40180	10.27
122	172.00	30348	7.76
123	173.00	7290	1.86
124	174.00	1239	0.32
125	177.20	238	0.06
126	178.15	3162	0.81
127	179.05	1817	0.46
128	180.00	3514	0.90
129	181.05	9794	2.50
130	182.00	19970	5.11
131	183.00	25158	6.43
132	184.00	31201	7.98
133	185.00	38050	9.73
134	186.00	40924	10.46
135	187.00	10028	2.56
136	188.00	6714	1.72
137	189.00	868	0.22
138	191.05	296	0.08
139	192.00	4826	1.23
140	193.05	2875	0.74
141	194.00	49812	12.73

#	m/z	Abs. In	Rel. Int.
142	195.00	47017	12.02
143	196.00	122146	31.23
144	197.05	22307	5.70
145	198.00	241660	61.78
146	198.95	25629	6.55
147	200.00	43719	11.18
148	200.95	4204	1.07
149	201.95	533	0.14
150	207.00	2228	0.57
151	208.05	2526	0.65
152	209.05	20084	5.13
153	210.05	23473	6.00
154	211.00	50268	12.85
155	212.05	22070	5.64
156	213.00	91327	23.35
157	214.00	31220	7.98
158	215.00	18164	4.64
159	216.00	12508	3.20
160	216.95	1191	0.30
161	218.00	1618	0.41
162	224.00	2599	0.66
163	225.05	1510	0.39
164	226.05	26426	6.76
165	227.05	28172	7.20
166	228.05	72774	18.60
167	229.05	16399	4.19
168	230.05	148867	38.06
169	231.00	25292	6.47
170	232.05	29923	7.65
171	233.00	4730	1.21
172	233.95	605	0.15
173	238.15	734	0.19
174	239.10	6298	1.61
175	240.15	7887	2.02
176	241.10	72847	18.62
177	242.10	80234	20.51
178	243.10	187193	47.86
179	244.15	60066	15.36
180	245.10	391154	100.00
181	246.05	51306	13.12
182	247.05	74480	19.04
183	248.05	8926	2.28
184	249.05	953	0.24
185	253.10	1460	0.37
186	254.15	1011	0.26
187	255.10	15329	3.92
188	256.15	16105	4.12
189	257.10	40538	10.36
190	258.15	9549	2.44
191	259.10	84319	21.56
192	260.05	12047	3.08
193	261.10	15478	3.96
194	262.05	2350	0.60
195	263.05	335	0.09
196	264.10	220	0.06
197	269.15	730	0.19
198	270.15	629	0.16
199	271.15	1493	0.38
200	272.25	533	0.14
201	273.15	2823	0.72
202	274.20	558	0.14
203	275.20	654	0.17
204	276.20	290	0.07
205	277.10	369	0.09

Figure S15. Mass chart of compound **4**.

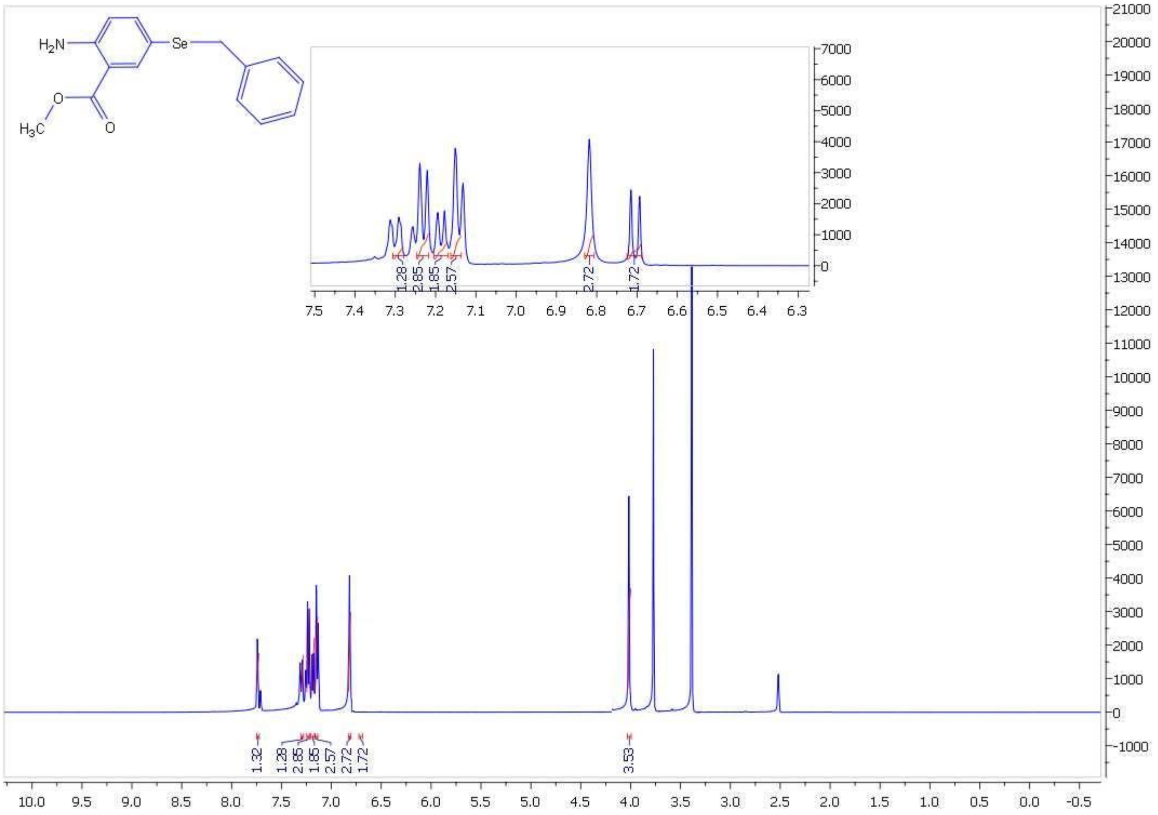

Figure S16. ^{1}H NMR chart of compound **5**.

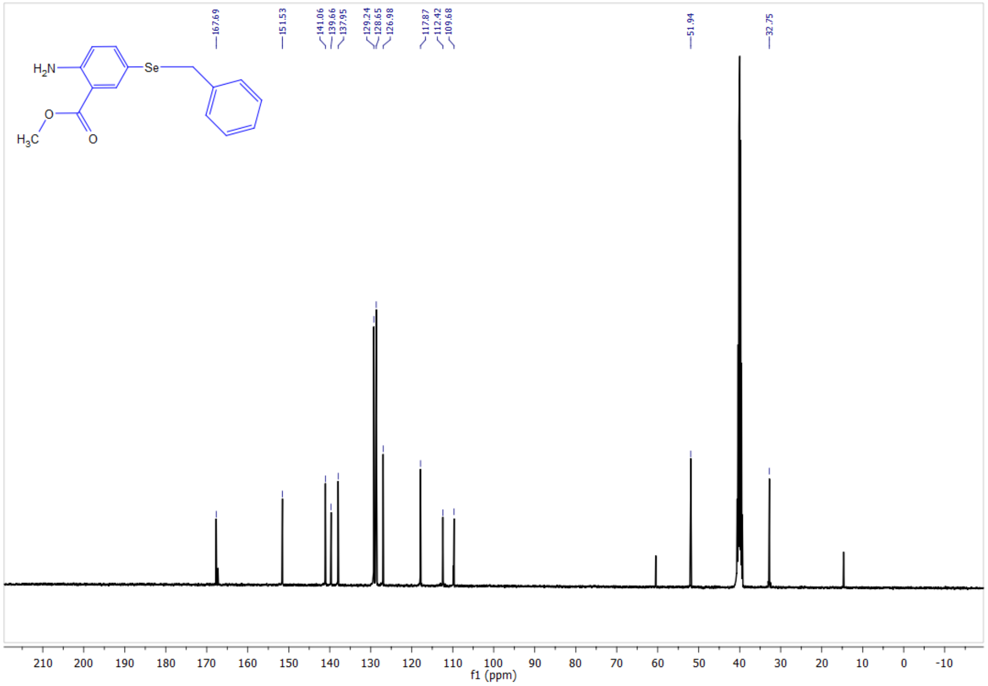

Figure S17. ^{13}C NMR chart of compound **5**.

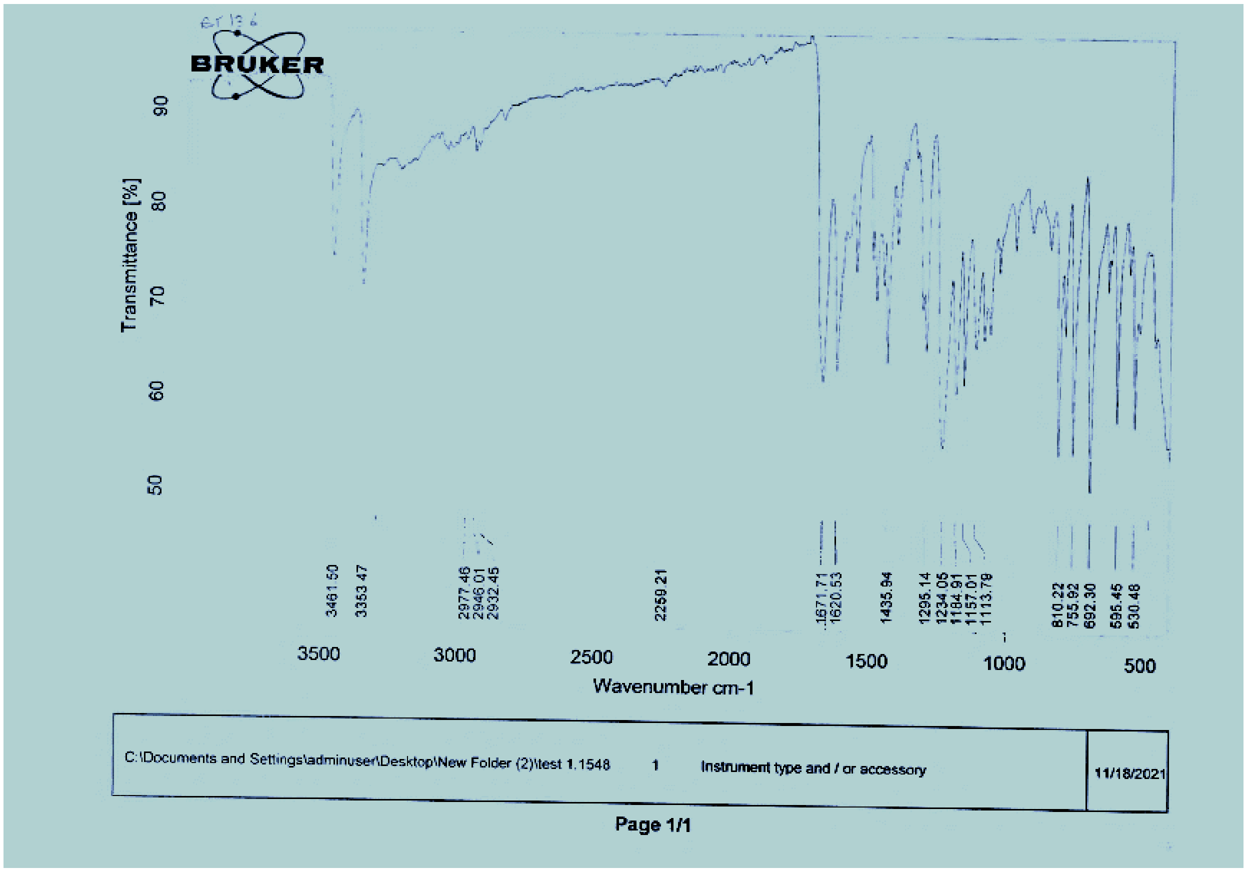

Figure S18. IR chart of compound **5**.

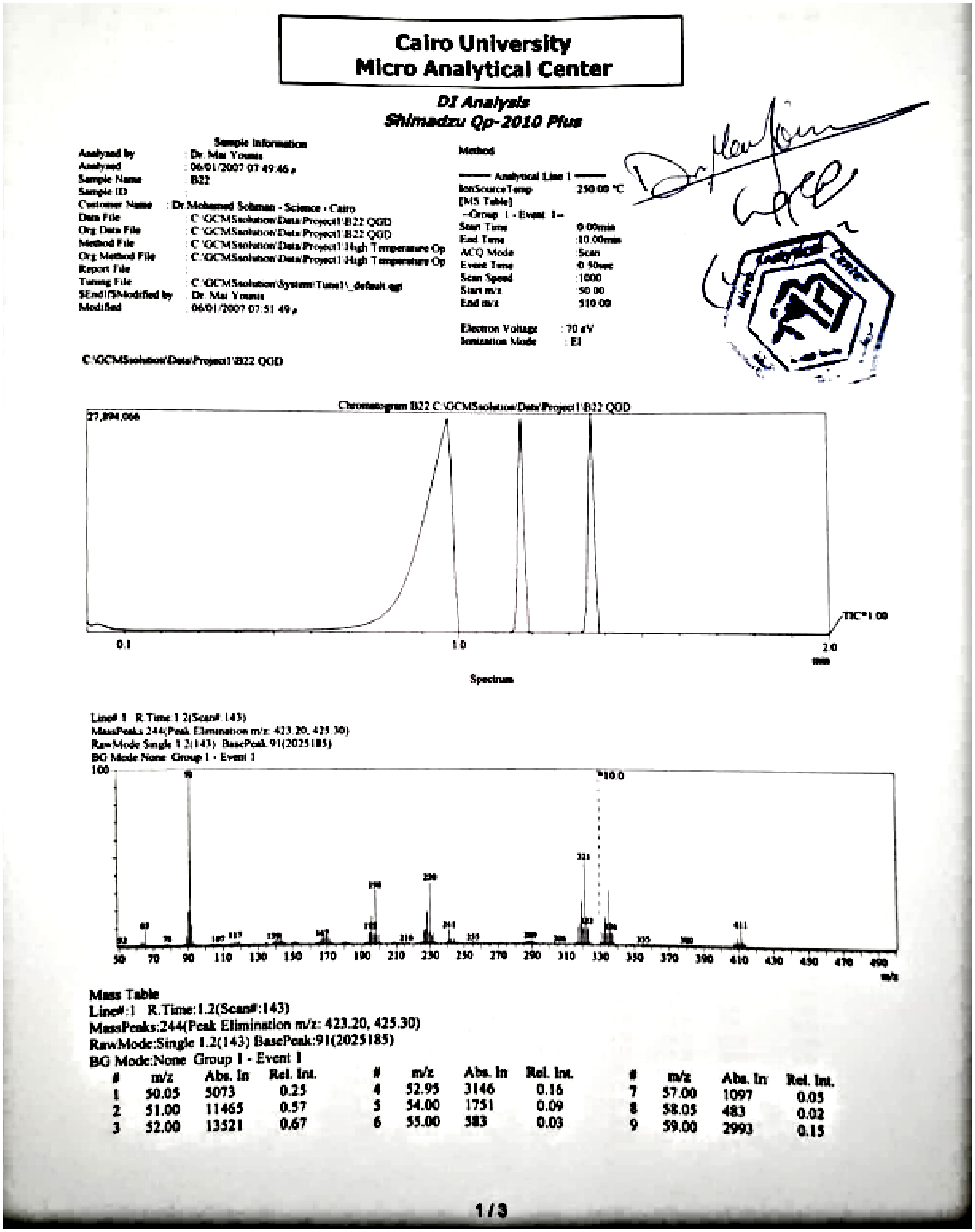

Figure S19. Mass chart of compound **5**.

#	m/z	Abs. In	Rel. Int.
10	60.05	391	0.02
11	61.05	3772	0.19
12	62.05	17107	0.84
13	63.00	56926	2.81
14	64.05	36812	1.82
15	65.00	176463	8.71
16	65.95	12878	0.64
17	67.00	1580	0.08
18	68.05	483	0.02
19	69.10	985	0.05
20	70.10	1497	0.07
21	71.10	1663	0.08
22	72.05	379	0.02
23	73.05	2080	0.10
24	74.05	4921	0.24
25	75.05	5212	0.26
26	76.05	6114	0.30
27	77.05	10886	0.54
28	78.05	20811	1.03
29	79.05	8101	0.40
30	80.00	5090	0.25
31	81.05	1605	0.08
32	82.15	1045	0.05
33	83.10	2590	0.13
34	84.15	2599	0.13
35	85.15	4470	0.22
36	86.05	4174	0.21
37	87.15	6312	0.31
38	88.15	20787	1.03
39	89.15	110480	5.46
40	90.15	391518	19.33
41	91.05	202518	100.00
42	92.05	228419	11.28
43	93.05	46032	2.27
44	94.00	4180	0.21
45	95.00	8522	0.42
46	96.15	1388	0.07
47	97.10	2596	0.13
48	98.10	2490	0.12
49	99.10	2721	0.13
50	100.15	911	0.04
51	101.10	2750	0.14
52	102.10	4402	0.22
53	103.10	7242	0.36
54	104.05	11796	0.58
55	105.05	7012	0.35
56	106.10	11636	0.57
57	107.05	13258	0.65
58	108.00	3873	0.19
59	109.15	1378	0.07
60	110.05	979	0.05
61	111.05	2440	0.12
62	112.05	2793	0.14
63	113.05	9979	0.49
64	114.05	12822	0.63
65	115.00	27724	1.37
66	116.05	18052	0.89
67	117.00	53988	2.67
68	118.05	26835	1.33
69	119.05	43451	2.15
70	120.05	15785	0.78
71	121.10	4878	0.24
72	122.05	2063	0.10
73	123.10	1527	0.08
74	124.05	682	0.03
75	125.05	4002	0.20
76	126.05	5459	0.27
77	127.05	10480	0.52
78	128.05	10844	0.54

#	m/z	Abs. In	Rel. Int.
79	129.05	9313	0.46
80	130.05	11086	0.55
81	131.10	5054	0.25
82	132.05	10987	0.54
83	133.10	3684	0.18
84	134.15	9264	0.46
85	135.05	30121	1.49
86	136.05	4109	0.20
87	137.05	5343	0.26
88	138.05	7879	0.39
89	139.00	35607	1.76
90	140.05	41787	2.06
91	141.00	77244	3.81
92	142.05	47101	2.33
93	142.95	126529	6.25
94	143.95	48735	2.41
95	144.95	28945	1.43
96	146.05	23129	1.14
97	146.95	4067	0.20
98	148.15	1843	0.09
99	149.15	8597	0.42
100	150.15	20578	1.02
101	151.05	33271	1.64
102	152.05	32505	1.61
103	153.05	20872	1.03
104	154.05	7855	0.39
105	155.00	4855	0.24
106	155.95	3585	0.18
107	157.00	3398	0.17
108	157.95	2693	0.13
109	159.05	2274	0.11
110	160.05	4165	0.21
111	161.00	602	0.03
112	162.05	482	0.02
113	163.05	3714	0.18
114	164.05	12889	0.64
115	165.05	22436	1.11
116	166.05	47284	2.33
117	167.05	66556	3.29
118	168.00	102111	5.04
119	169.05	77098	3.81
120	169.95	174770	8.63
121	170.95	74461	3.68
122	172.00	39018	1.93
123	172.95	11808	0.58
124	173.95	1305	0.06
125	176.15	889	0.04
126	177.15	2401	0.12
127	178.15	5969	0.29
128	179.15	10187	0.50
129	180.15	36701	1.81
130	181.10	18865	0.93
131	182.15	14965	0.74
132	183.05	3441	0.17
133	184.00	2350	0.12
134	185.10	1281	0.06
135	186.05	1167	0.06
136	190.05	602	0.03
137	191.05	1736	0.09
138	192.05	12467	0.62
139	193.05	17901	0.88
140	194.05	131489	6.49
141	195.05	146464	7.23
142	196.00	323593	15.98
143	197.05	113333	5.60
144	198.00	620129	30.62
145	198.95	99120	4.89
146	200.00	113441	5.60
147	200.95	16159	0.80

#	m/z	Abs. In	Rel. Int.
148	201.95	1028	0.05
149	204.25	5464	0.27
150	205.15	701	0.03
151	206.20	1020	0.05
152	207.25	2877	0.14
153	208.20	14284	0.71
154	209.15	17645	0.87
155	210.05	5896	0.29
156	211.15	1898	0.09
157	212.05	5471	0.27
158	213.10	2154	0.11
159	214.05	4336	0.21
160	215.15	994	0.05
161	216.10	6855	0.34
162	217.05	395	0.02
163	218.10	921	0.05
164	221.20	846	0.04
165	223.15	1595	0.08
166	224.10	14468	0.71
167	225.15	19403	0.96
168	226.10	153471	7.58
169	227.15	166163	8.20
170	228.10	368934	18.22
171	229.15	120571	5.95
172	230.10	706377	34.88
173	231.05	94541	4.67
174	232.10	132870	6.56
175	233.05	15116	0.75
176	234.05	960	0.05
177	238.15	969	0.05
178	239.25	4131	0.20
179	240.25	35113	1.73
180	241.25	155831	7.69
181	242.10	54386	2.69
182	243.15	12588	0.62
183	244.10	56178	2.77
184	245.05	8044	0.40
185	246.10	9770	0.48
186	247.10	1503	0.07
187	254.35	1823	0.09
188	255.25	10721	0.53
189	256.15	3888	0.19
190	257.15	1891	0.09
191	258.15	4289	0.21
192	259.15	3074	0.15
193	260.15	2910	0.14
194	261.15	2231	0.11
195	262.20	1594	0.08
196	283.25	376	0.02
197	284.25	1803	0.09
198	285.20	7185	0.35
199	286.20	14742	0.73
200	287.15	23239	1.15
201	288.20	24534	1.21
202	289.15	36611	1.81
203	290.15	36028	1.78
204	291.10	11612	0.57
205	292.15	6462	0.32
206	293.15	552	0.03
207	306.20	302	0.01
208	308.10	636	0.03
209	310.05	707	0.03
210	314.25	1728	0.09
211	315.20	18481	0.91
212	316.25	23945	1.18
213	317.20	196281	9.69
214	318.25	227987	11.26
215	319.20	494574	24.42
216	320.25	188395	9.30

Figure S20. Mass chart of compound **5**.

#	m/z	Abs. In	Rel. Int.
217	321.20	949119	46.87
218	322.15	206457	10.19
219	323.15	182324	9.00
220	324.05	37472	1.85
221	325.05	4024	0.20
222	329.25	824	0.04
223	330.25	1720	0.08
224	331.20	13207	0.65
225	332.25	14297	0.71
226	333.20	31734	1.57

#	m/z	Abs. In	Rel. Int.
227	334.25	12617	0.62
228	335.20	61590	3.04
229	336.15	14251	0.70
230	337.20	12284	0.61
231	338.15	2137	0.11
232	347.20	470	0.02
233	349.20	1524	0.08
234	355.20	474	0.02
235	378.20	226	0.01
236	380.20	604	0.03

#	m/z	Abs. In	Rel. Int.
237	407.30	3812	0.19
238	408.35	4545	0.22
239	409.25	10425	0.51
240	410.35	4385	0.22
241	411.25	19850	0.98
242	412.25	5834	0.29
243	413.25	4131	0.20
244	414.15	786	0.04

Figure S21. Mass chart of compound **5**.

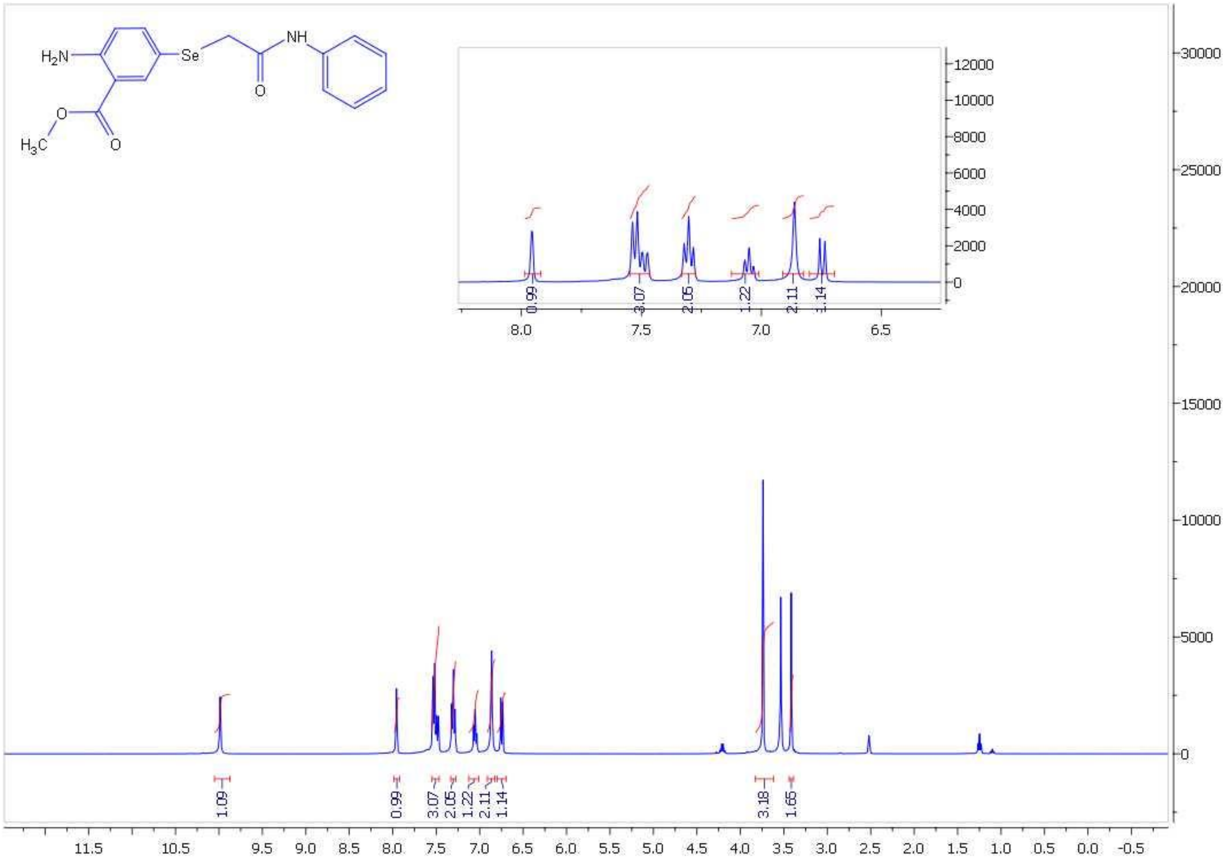

Figure S22. ^{1}H NMR chart of compound **6**.

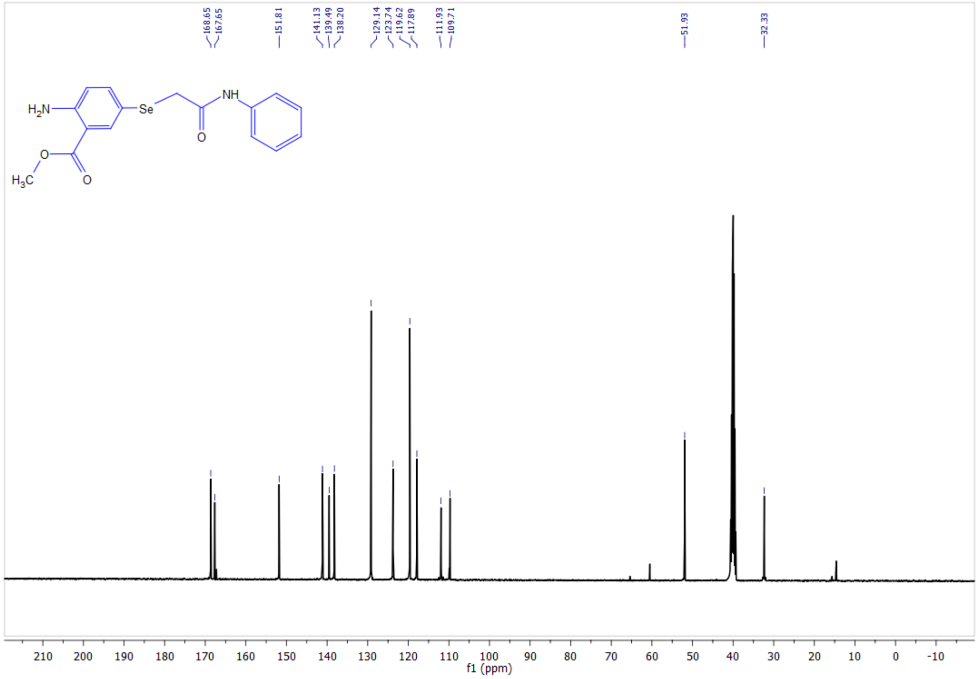

Figure S23. ^{13}C NMR chart of compound **6**.

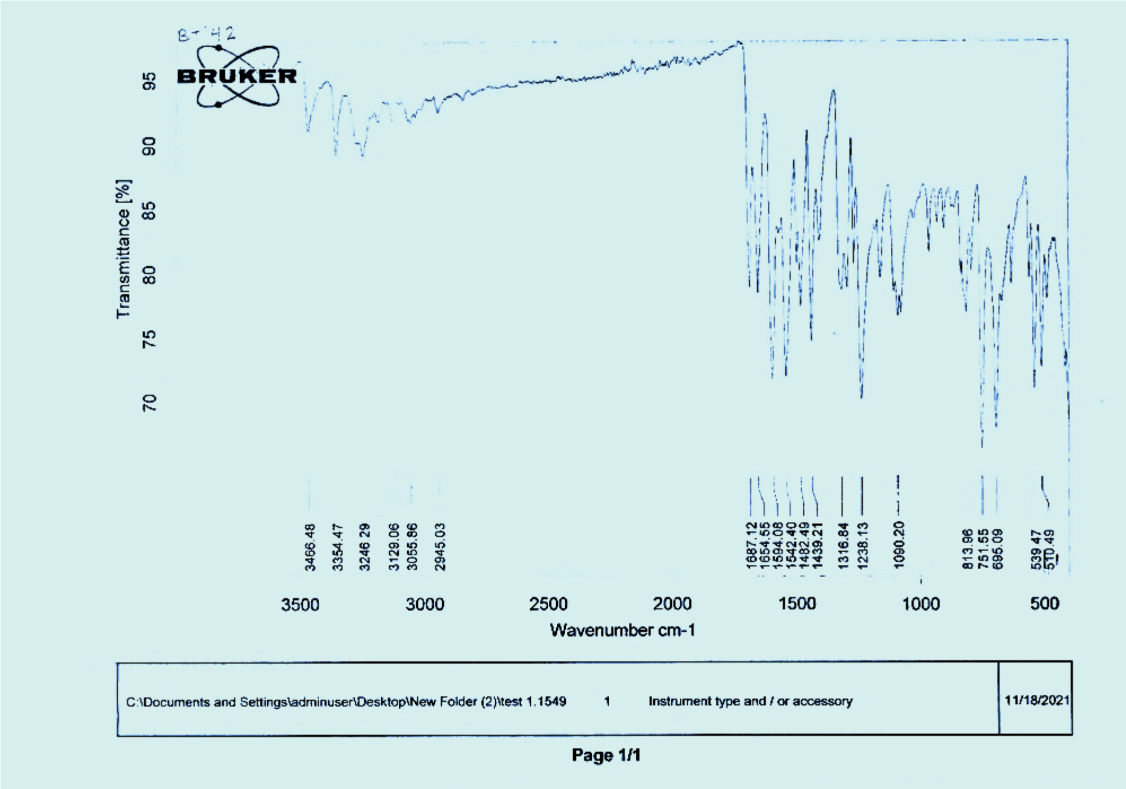

Figure S24. IR chart of compound **6**.

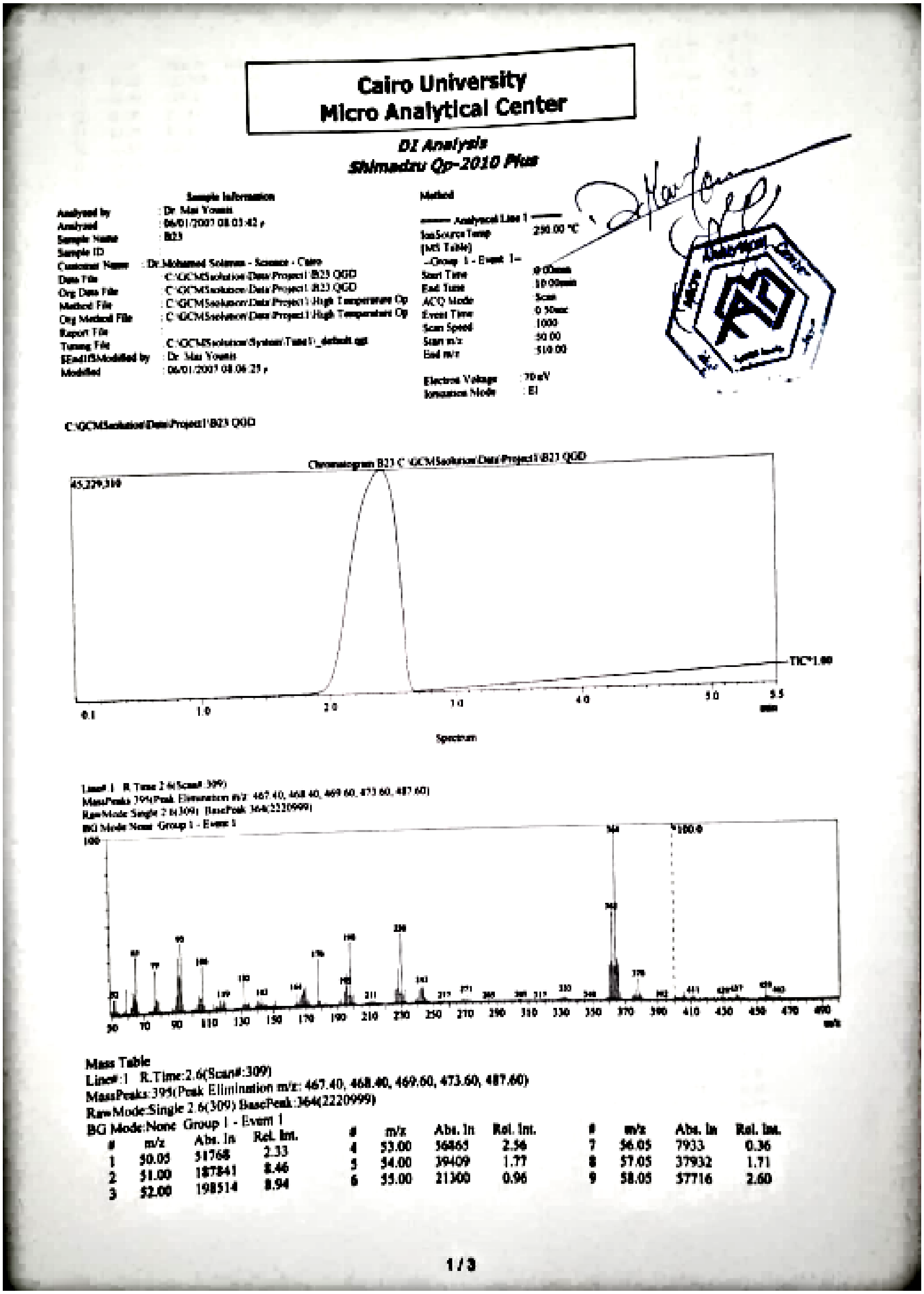

Figure S25. Mass chart of compound **6**.

#	m/z	Abs. In	Rel. Int.
10	59.00	323380	14.56
11	60.00	19518	0.88
12	61.05	25294	1.14
13	62.05	85663	3.86
14	63.05	284974	12.83
15	64.05	250676	11.29
16	65.00	718538	32.35
17	66.00	152620	6.87
18	66.95	28433	1.28
19	68.05	8877	0.40
20	69.05	15359	0.69
21	70.05	8547	0.38
22	71.05	14796	0.67
23	72.05	7256	0.33
24	73.05	29968	1.35
25	74.05	26922	1.21
26	75.05	38018	1.71
27	76.05	89648	4.04
28	77.05	535908	24.13
29	78.05	171575	7.73
30	79.05	144276	6.50
31	80.05	37639	1.69
32	81.05	11176	0.50
33	82.15	5419	0.24
34	83.10	15562	0.70
35	84.15	7935	0.36
36	85.10	16374	0.74
37	86.15	16002	0.72
38	87.10	69636	3.14
39	88.15	32733	1.47
40	89.15	88448	3.98
41	90.15	210874	9.49
42	91.10	688561	31.00
43	92.15	463765	20.88
44	93.10	862055	38.81
45	94.05	112159	5.05
46	95.00	27708	1.25
47	96.10	5274	0.24
48	97.10	9146	0.41
49	98.15	5237	0.24
50	99.10	12962	0.58
51	100.15	13487	0.61
52	101.10	60012	2.70
53	102.15	30642	1.38
54	103.15	88585	3.99
55	104.10	204116	9.19
56	105.15	159966	7.20
57	106.10	568420	25.59
58	107.10	76871	3.46
59	108.05	12580	0.57
60	109.10	3719	0.17
61	110.15	3039	0.14
62	111.15	9856	0.44
63	112.15	15347	0.69
64	113.10	93678	4.22
65	114.15	34219	1.54
66	115.10	49390	2.22
67	116.15	38664	1.74
68	117.10	130407	5.87
69	118.15	63644	2.87
70	119.10	142945	6.44
71	120.10	98100	4.42
72	121.05	14291	0.64
73	122.10	5969	0.27
74	123.05	3081	0.14
75	124.15	1538	0.07
76	125.15	3955	0.18
77	126.15	7068	0.32
78	127.15	19469	0.88
79	128.15	17920	0.81
80	129.15	16582	0.75
81	130.15	23991	1.08
82	131.15	62358	2.81
83	132.10	333977	15.04
84	133.15	66067	2.97
85	134.15	78340	3.53
86	135.15	69517	3.13
87	136.15	11769	0.53
88	137.10	7681	0.35
89	138.15	13090	0.59
90	139.15	52322	2.36
91	140.10	117575	5.29
92	141.05	90195	4.06
93	142.05	52676	2.37
94	143.00	141599	6.38
95	144.00	54009	2.43
96	145.05	43425	1.96
97	146.05	54032	2.43
98	147.10	12569	0.57
99	148.10	17756	0.80
100	149.15	8208	0.37
101	150.15	38743	1.74
102	151.10	102964	4.64
103	152.05	16710	0.75
104	153.10	11177	0.50
105	154.10	14711	0.66
106	155.05	15641	0.70
107	156.05	11100	0.50
108	157.05	15214	0.69
109	158.05	8233	0.37
110	159.10	16986	0.76
111	160.05	4503	0.20
112	161.15	4537	0.20
113	162.15	5173	0.23
114	163.15	35647	1.60
115	164.10	195450	8.80
116	165.05	50811	2.29
117	166.05	92270	4.15
118	167.05	149172	6.72
119	168.05	172761	7.78
120	169.05	226041	10.18
121	170.00	257615	11.60
122	171.00	96798	4.36
123	172.00	69998	3.15
124	173.00	21033	0.95
125	174.10	7897	0.36
126	175.15	17735	0.80
127	176.15	7737	0.35
128	177.15	62536	2.82
129	178.10	605762	27.27
130	179.05	72024	3.24
131	180.05	20815	0.94
132	181.10	26969	1.21
133	182.05	36544	1.65
134	183.10	28086	1.26
135	184.05	26423	1.19
136	185.00	19938	0.90
137	186.00	10751	0.48
138	187.05	6516	0.29
139	188.25	4750	0.21
140	189.15	25047	1.13
141	190.15	9994	0.45
142	191.15	57570	2.59
143	192.10	82754	3.73
144	193.05	33585	1.51
145	194.05	172249	7.76
146	195.05	249002	11.21
147	196.00	413670	18.63
148	197.05	127657	5.75
149	198.00	802486	36.13
150	198.95	111117	5.00
151	200.00	147049	6.62
152	201.00	27327	1.23
153	202.05	6591	0.30
154	203.15	3651	0.16
155	204.15	2634	0.12
156	205.10	8610	0.39
157	206.05	3512	0.16
158	207.05	11680	0.53
159	208.05	15928	0.72
160	209.05	33769	1.52
161	210.05	30291	1.36
162	211.05	55042	2.48
163	212.05	30408	1.37
164	213.05	31320	1.41
165	214.05	18869	0.85
166	215.05	9367	0.42
167	216.05	19840	0.89
168	217.05	4333	0.20
169	218.05	4479	0.20
170	219.10	2612	0.12
171	220.10	778	0.04
172	221.15	1397	0.06
173	222.15	1142	0.05
174	223.15	4881	0.22
175	224.05	17976	0.81
176	225.15	20916	0.94
177	226.10	174902	7.87
178	227.15	189200	8.52
179	228.05	447454	20.15
180	229.15	133404	6.01
181	230.05	899020	40.48
182	231.05	114924	5.17
183	232.05	173318	7.80
184	233.05	21637	0.97
185	234.05	3484	0.16
186	235.15	3179	0.14
187	236.15	3807	0.17
188	237.05	8985	0.40
189	238.15	10887	0.49
190	239.10	48174	2.17
191	240.10	75681	3.41
192	241.10	157647	7.10
193	242.15	186228	8.38
194	243.10	230793	10.39
195	244.05	179124	8.07
196	245.05	65413	2.95
197	246.05	36727	1.65
198	247.20	22822	1.03
199	248.15	7827	0.35
200	249.15	2124	0.10
201	250.25	1855	0.08
202	251.20	7351	0.33
203	252.15	3123	0.14
204	253.15	6626	0.30
205	254.15	7024	0.32
206	255.10	19212	0.87
207	256.15	19273	0.87
208	257.10	29007	1.31
209	258.15	10175	0.46
210	259.20	21718	0.98
211	260.15	6061	0.27
212	261.15	4356	0.20
213	262.15	3391	0.15
214	263.15	3353	0.15
215	264.10	1687	0.08
216	265.15	3515	0.16

Figure S26. Mass chart of compound **6**.

#	m/z	Abs. In	Rel. Int.	#	m/z	Abs. In	Rel. Int.	#	m/z	Abs. In	Rel. Int.
217	266.15	2637	0.12	277	326.15	836	0.04	337	387.30	279	0.01
218	267.10	18146	0.82	278	327.15	2834	0.13	338	388.25	396	0.02
219	268.15	20158	0.91	279	328.15	6030	0.27	339	389.35	651	0.03
220	269.10	46786	2.11	280	329.15	23275	1.05	340	390.25	1290	0.06
221	270.15	15694	0.71	281	330.15	29151	1.31	341	391.35	1022	0.05
222	271.10	96137	4.33	282	331.15	50733	2.28	342	392.30	1966	0.09
223	272.05	16070	0.72	283	332.15	33893	1.53	343	393.35	1297	0.06
224	273.10	21305	0.96	284	333.15	88344	3.98	344	394.30	940	0.04
225	274.05	3890	0.18	285	334.05	24078	1.08	345	395.30	473	0.02
226	275.15	1899	0.09	286	335.15	19856	0.89	346	396.45	384	0.02
227	276.25	1345	0.06	287	336.15	4538	0.20	347	397.30	1070	0.05
228	277.20	2796	0.13	288	337.15	1006	0.05	348	398.45	585	0.03
229	278.15	1435	0.06	289	338.25	662	0.03	349	399.45	317	0.01
230	279.15	898	0.04	290	339.30	1508	0.07	350	400.20	370	0.02
231	280.15	727	0.03	291	340.25	578	0.03	351	401.20	289	0.01
232	281.15	2478	0.11	292	341.25	684	0.03	352	402.20	230	0.01
233	282.15	2409	0.11	293	342.25	584	0.03	353	403.20	257	0.01
234	283.10	5790	0.26	294	343.25	805	0.04	354	404.45	336	0.02
235	284.15	3531	0.16	295	344.20	1958	0.09	355	405.40	1146	0.05
236	285.10	12303	0.55	296	345.25	2706	0.12	356	406.35	447	0.02
237	286.15	5148	0.23	297	346.20	3890	0.18	357	407.40	271	0.01
238	287.10	5833	0.26	298	347.25	4640	0.21	358	409.45	410	0.02
239	288.25	2633	0.12	299	348.20	7434	0.33	359	410.50	282	0.01
240	289.15	7201	0.32	300	349.15	2578	0.12	360	411.50	383	0.03
241	290.15	2596	0.12	301	350.15	2611	0.12	361	412.50	284	0.01
242	291.20	2186	0.10	302	351.20	977	0.04	362	415.50	254	0.01
243	292.15	1131	0.05	303	352.15	764	0.03	363	417.50	207	0.01
244	293.25	1594	0.07	304	353.25	847	0.04	364	418.50	257	0.01
245	294.25	1118	0.05	305	354.15	553	0.02	365	419.50	354	0.02
246	295.20	5345	0.24	306	355.25	657	0.03	366	421.50	326	0.01
247	296.15	1761	0.08	307	356.25	704	0.03	367	423.50	396	0.02
248	297.25	811	0.04	308	357.25	3443	0.16	368	425.50	250	0.01
249	298.35	617	0.03	309	358.20	42783	1.93	369	426.50	212	0.01
250	299.30	823	0.04	310	359.25	41697	1.88	370	428.35	430	0.02
251	300.25	1273	0.06	311	360.20	448631	20.20	371	429.30	458	0.02
252	301.25	1429	0.06	312	361.25	490508	22.09	372	430.30	231	0.01
253	302.25	2061	0.09	313	362.20	113024	50.89	373	432.30	222	0.01
254	303.30	3346	0.15	314	363.25	433043	19.50	374	433.30	212	0.01
255	304.35	3895	0.18	315	364.20	222099	100.00	375	434.30	201	0.01
256	305.30	14001	0.63	316	365.15	516129	23.24	376	436.40	250	0.01
257	306.20	5601	0.25	317	366.15	453240	20.41	377	437.45	688	0.03
258	307.15	2298	0.10	318	367.15	94607	4.26	378	438.35	497	0.02
259	308.15	902	0.04	319	368.15	13453	0.61	379	439.50	431	0.02
260	309.30	892	0.04	320	369.15	2332	0.10	380	441.50	303	0.01
261	310.25	583	0.03	321	370.30	4468	0.20	381	442.50	233	0.01
262	311.35	721	0.03	322	371.25	1831	0.08	382	444.50	210	0.01
263	312.35	712	0.03	323	372.20	5146	0.23	383	451.50	289	0.01
264	313.35	1160	0.05	324	373.25	5093	0.23	384	452.50	233	0.01
265	314.35	803	0.04	325	374.25	54806	2.47	385	454.45	304	0.01
266	315.35	918	0.04	326	375.25	61887	2.79	386	455.40	1105	0.05
267	316.35	904	0.04	327	376.20	144442	6.50	387	456.35	499	0.02
268	317.30	4386	0.20	328	377.25	48025	2.16	388	457.10	265	0.01
269	318.25	2391	0.11	329	378.20	271025	12.20	389	458.15	443	0.02
270	319.25	2801	0.13	330	379.15	67760	3.05	390	459.10	218	0.01
271	320.25	1770	0.08	331	380.20	56982	2.57	391	460.20	433	0.02
272	321.20	4260	0.19	332	381.15	13270	0.60	392	462.20	287	0.01
273	322.25	2058	0.09	333	382.15	2182	0.10	393	463.40	473	0.02
274	323.25	2501	0.11	334	383.20	484	0.02	394	464.40	252	0.01
275	324.25	848	0.04	335	384.20	236	0.01	395	466.40	212	0.01
276	325.15	508	0.02	336	386.20	215	0.01				

Figure S27. Mass chart of compound **6**.

*4-((2-(methoxycarbonyl)-4-(methylselanyl)phenyl)amino)-4-oxobut-2-enoic acid (**7**)*

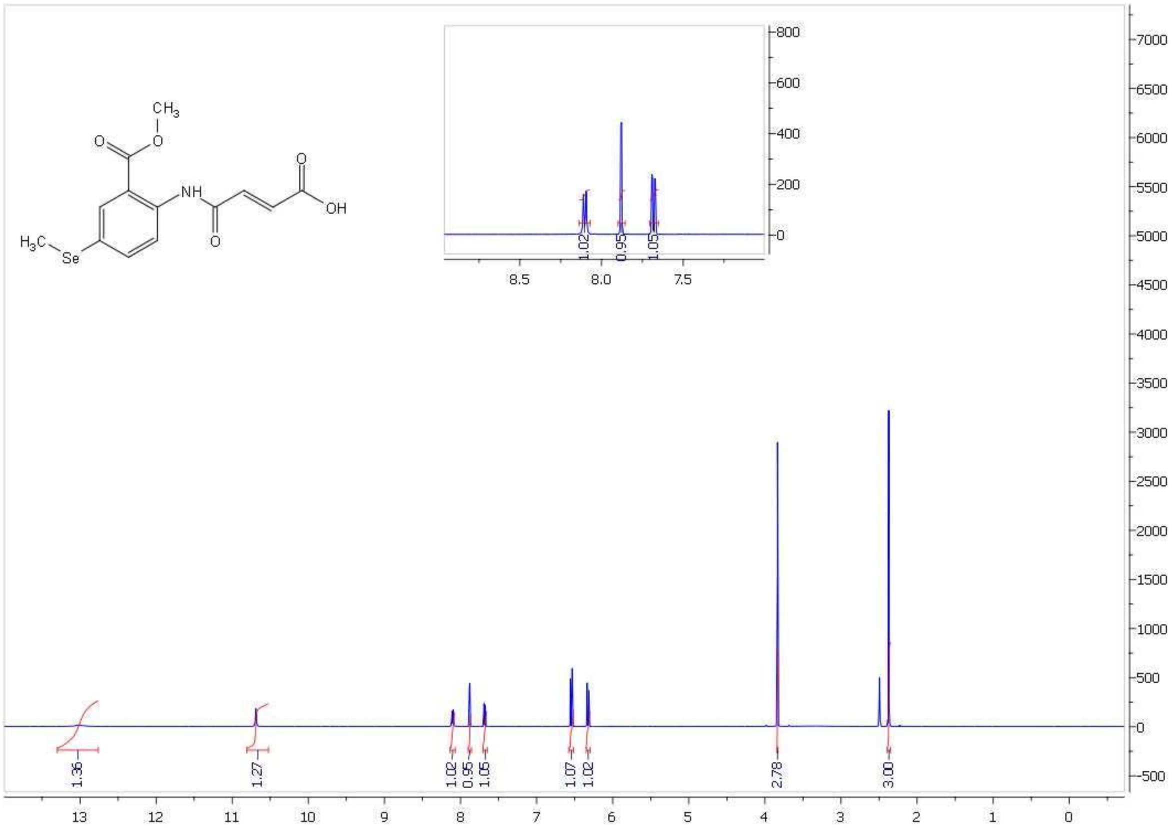

Figure S28. ^{1}H NMR chart of compound **7**.

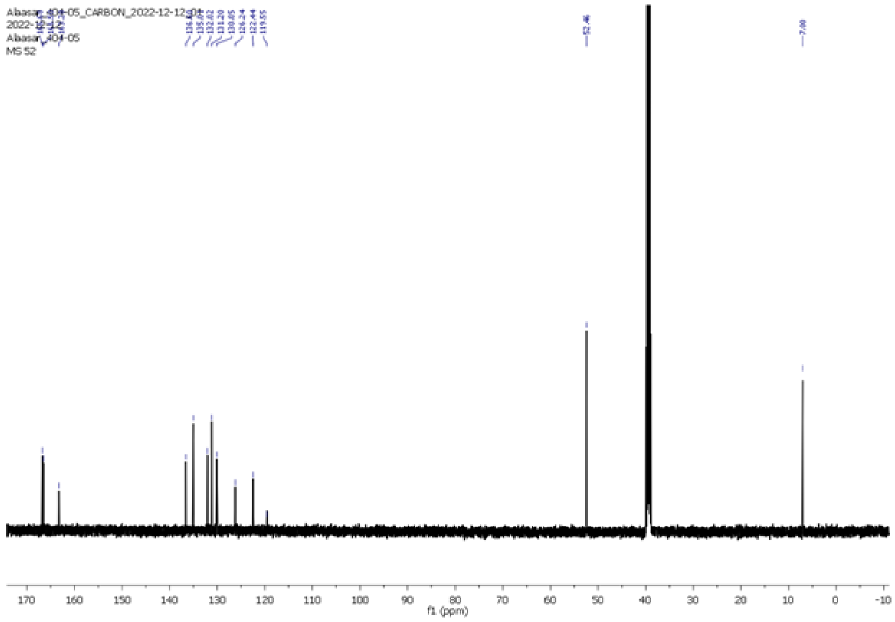

Figure S29. ^{13}C NMR chart of compound **7**.

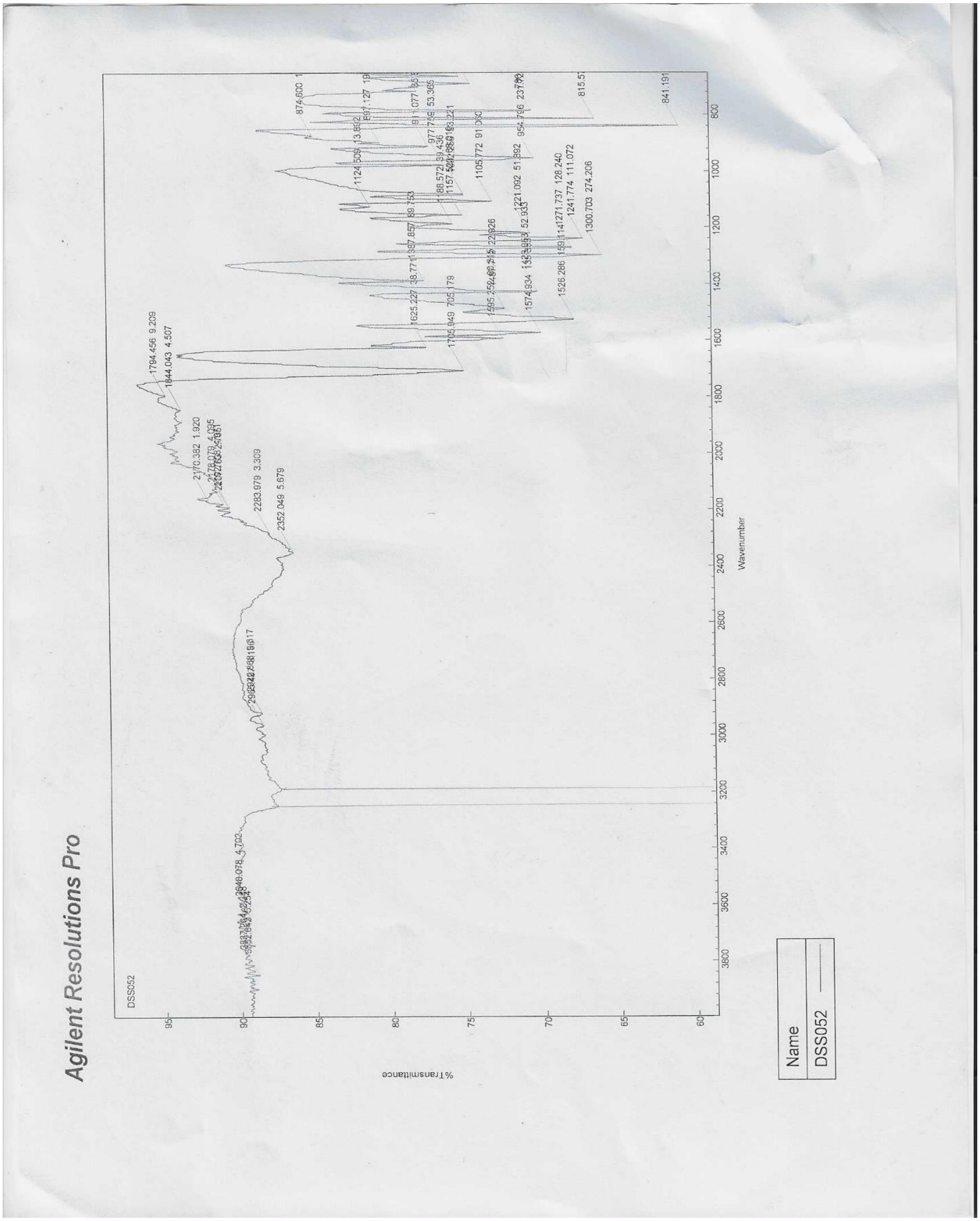

Figure S30. IR chart of compound **7**.

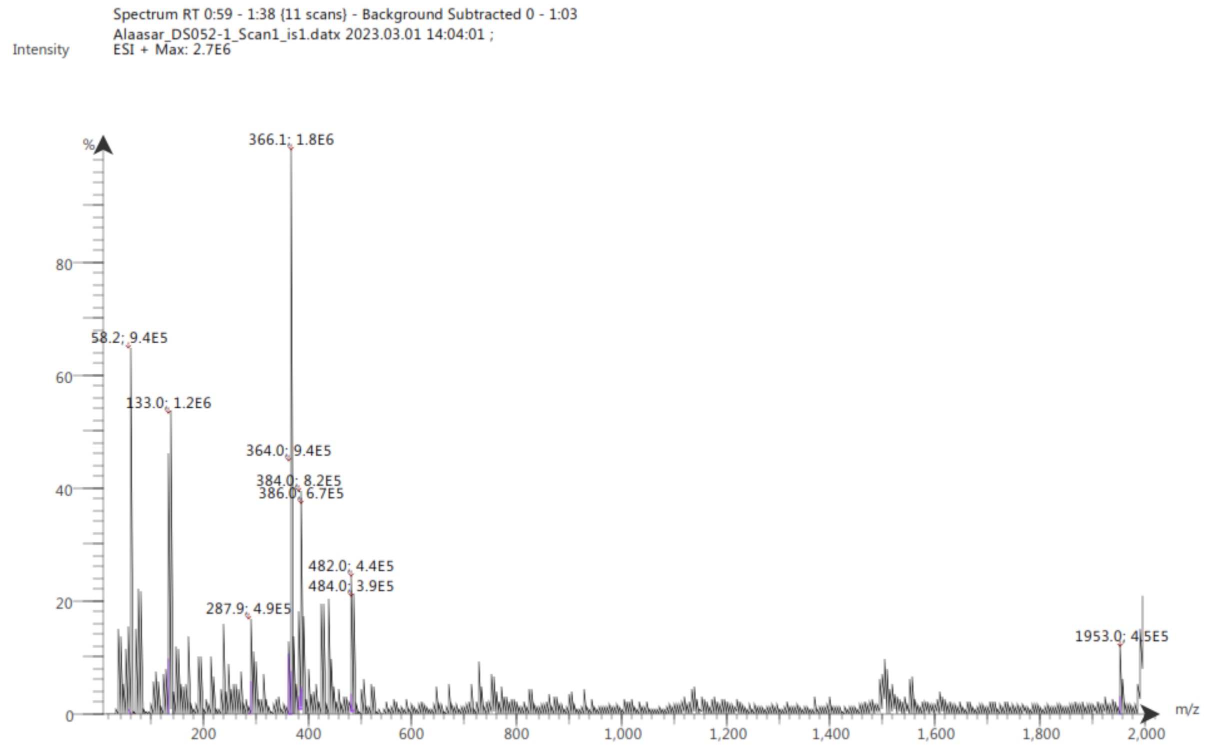

Figure S31. MS chart of compound **7**.

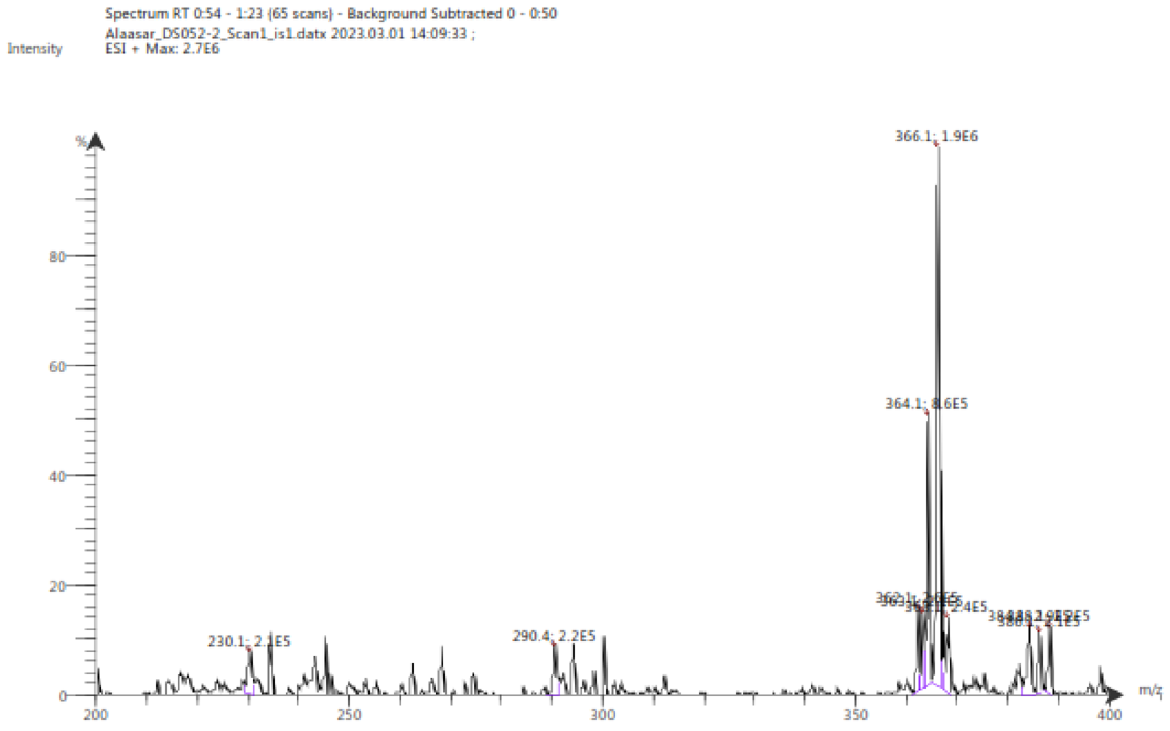

Figure S32. MS chart of compound **7**.

*4-((2-(methoxycarbonyl)-4-(methylselanyl)phenyl)amino)-4-oxobut-2-enoic acid (**8**)*

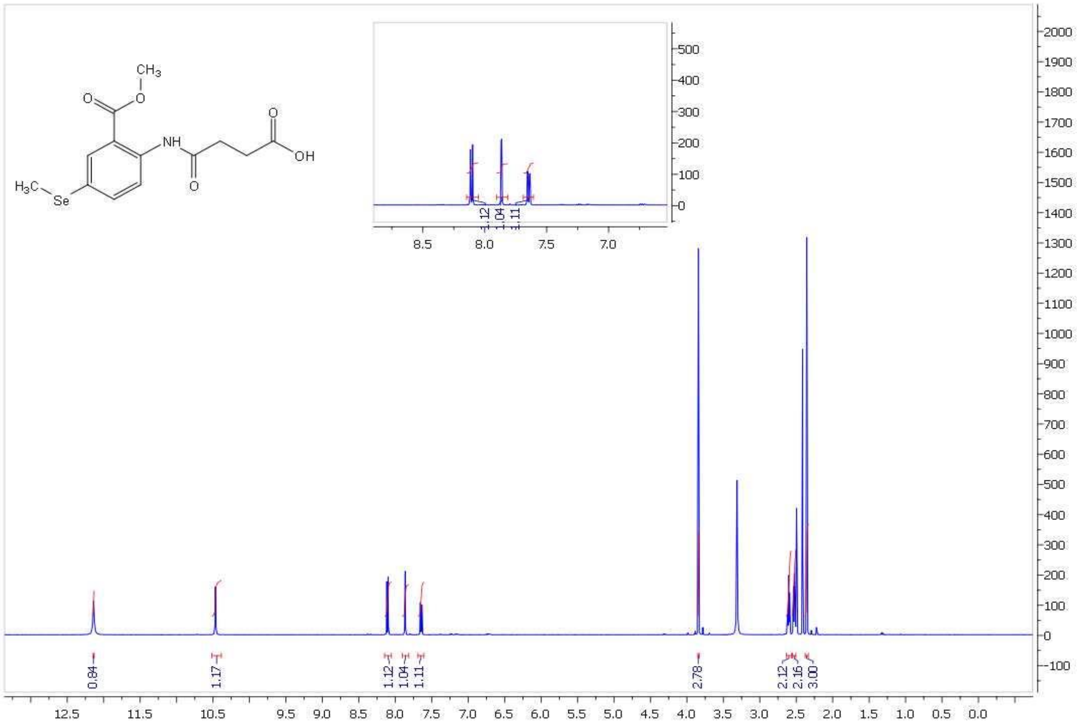

Figure S33. ^{1}H NMR chart of compound **8**.

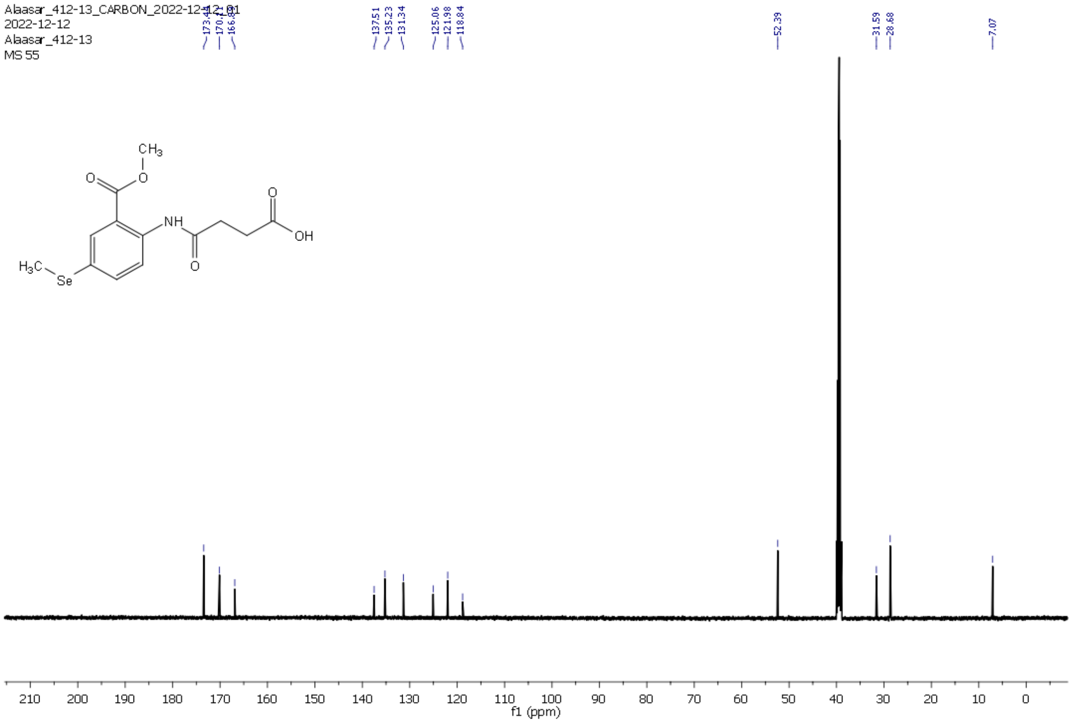

Figure S34. ^{13}C NMR chart of compound **8**.

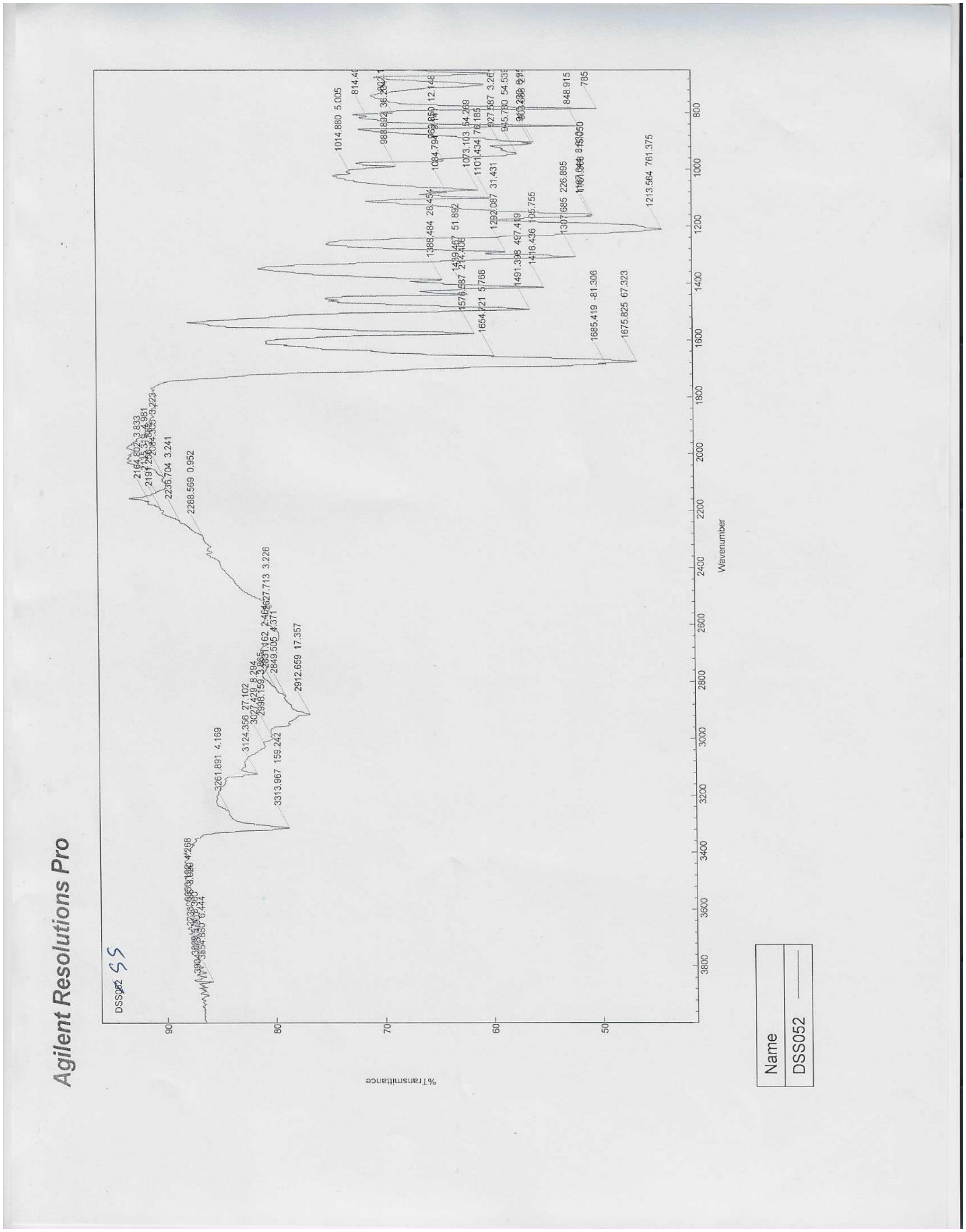

Figure S35. IR chart of compound **8**.

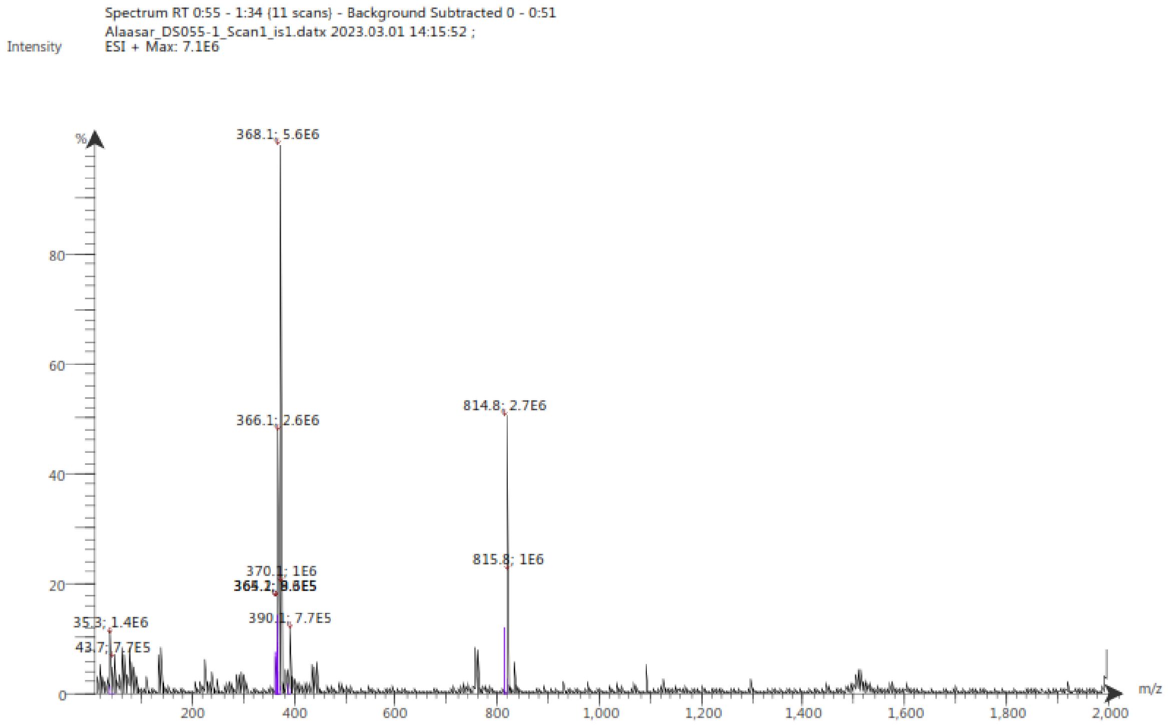

Figure S36. MS chart of compound **8**.

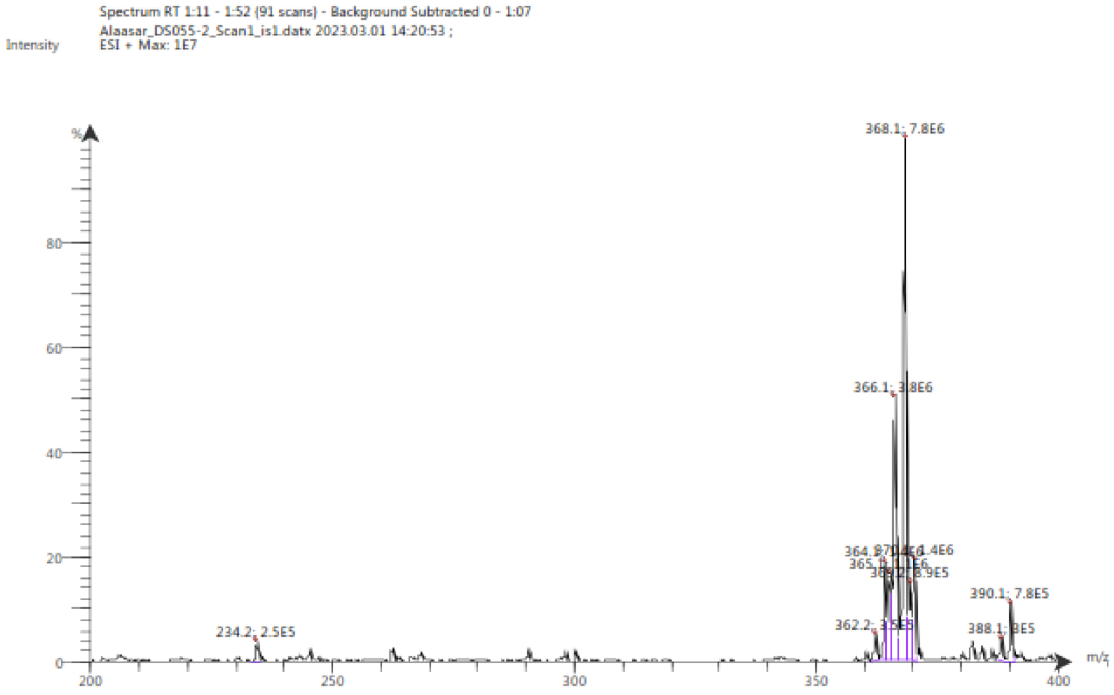

Figure S37. MS chart of compound **8**.

Supporting Information

*4-((2-(methoxycarbonyl)-4-(methylselanyl)phenyl)amino)-4-oxobut-2-enoic acid (**9**)*

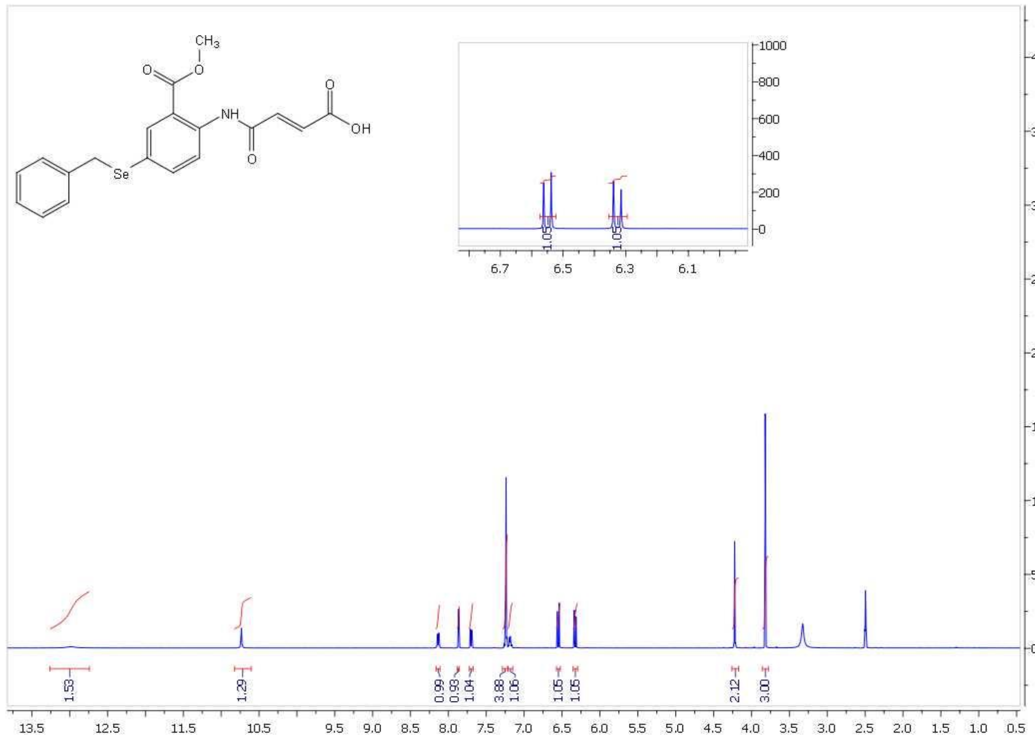

Figure S38. ^{1}H NMR chart of compound **9**.

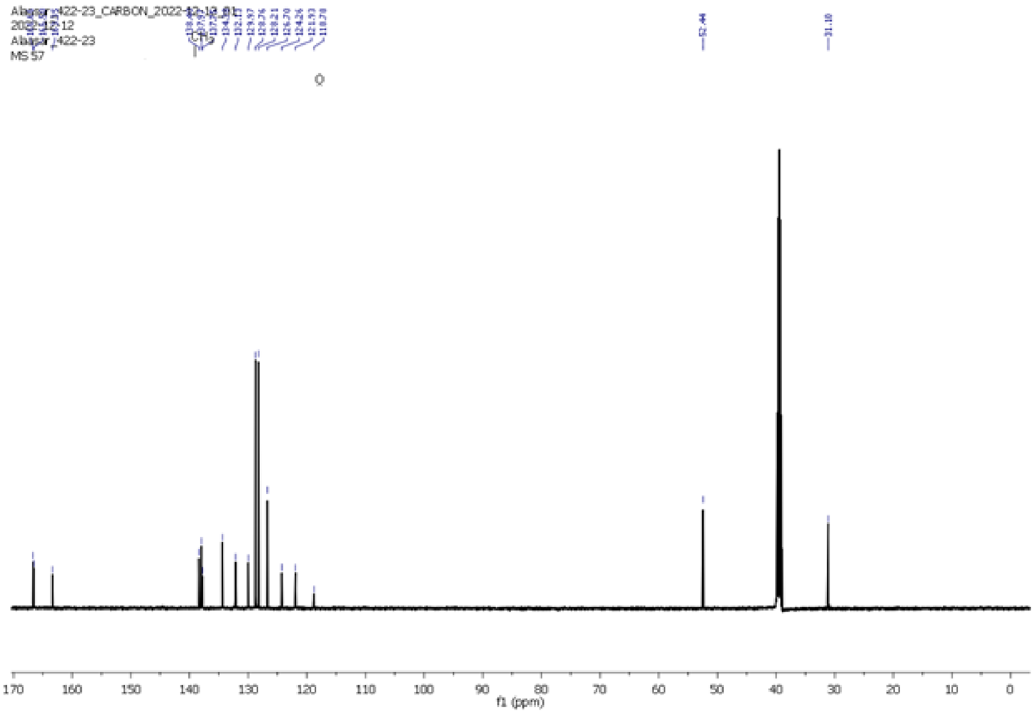

Figure S39. ^{13}C NMR chart of compound **9**.

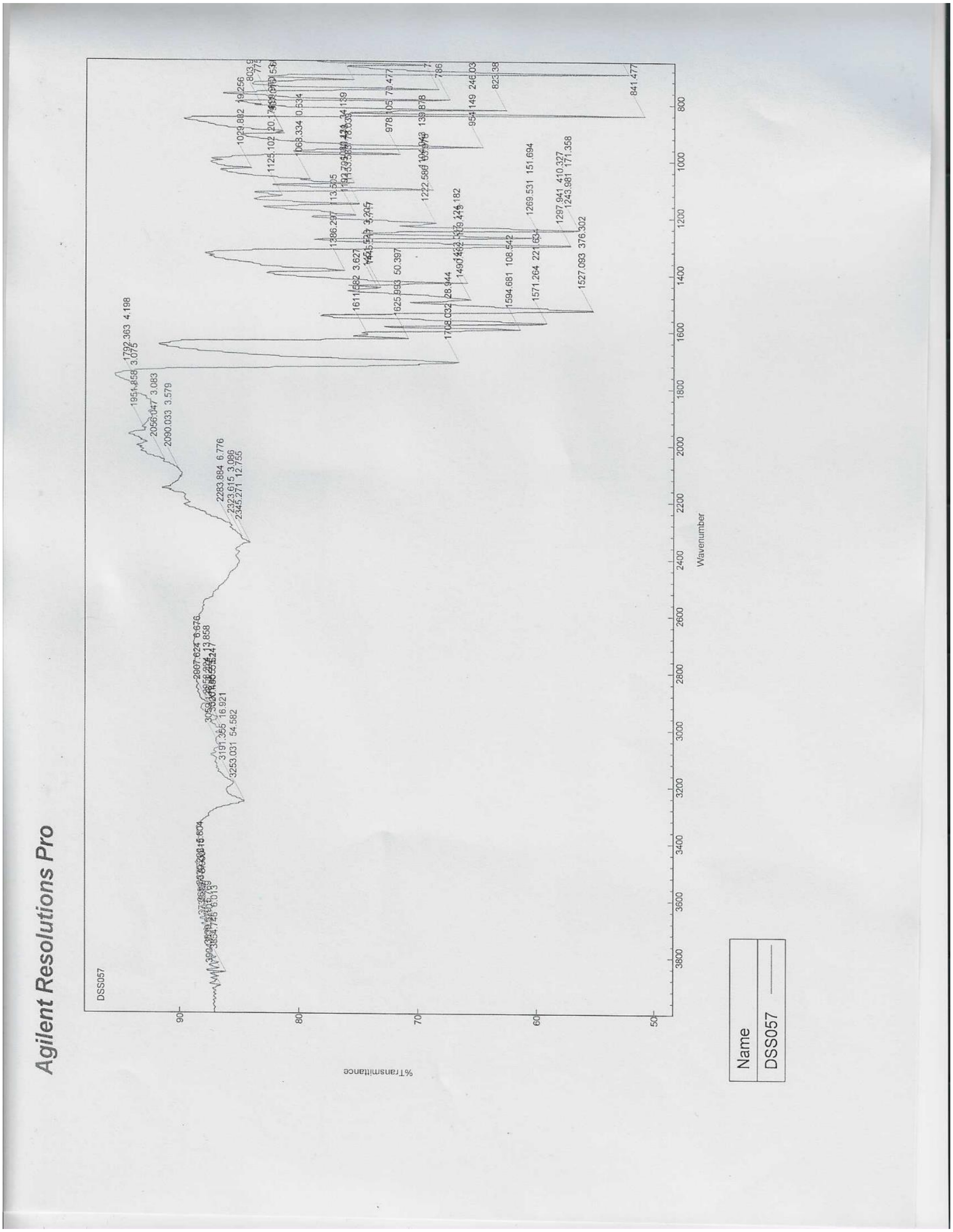

Figure S40. IR chart of compound **9**.

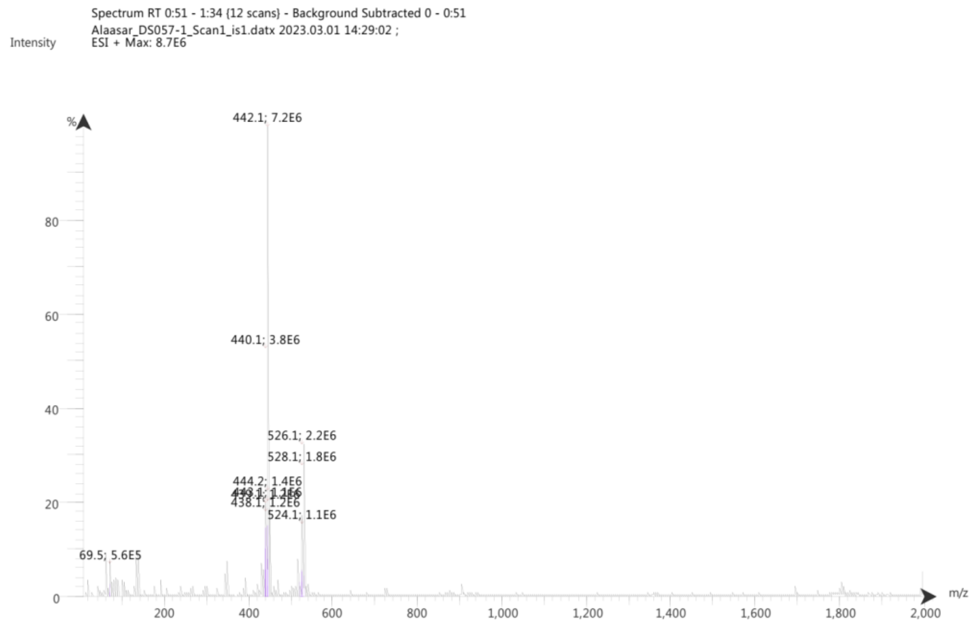

Figure S41. MS chart of compound **9**.

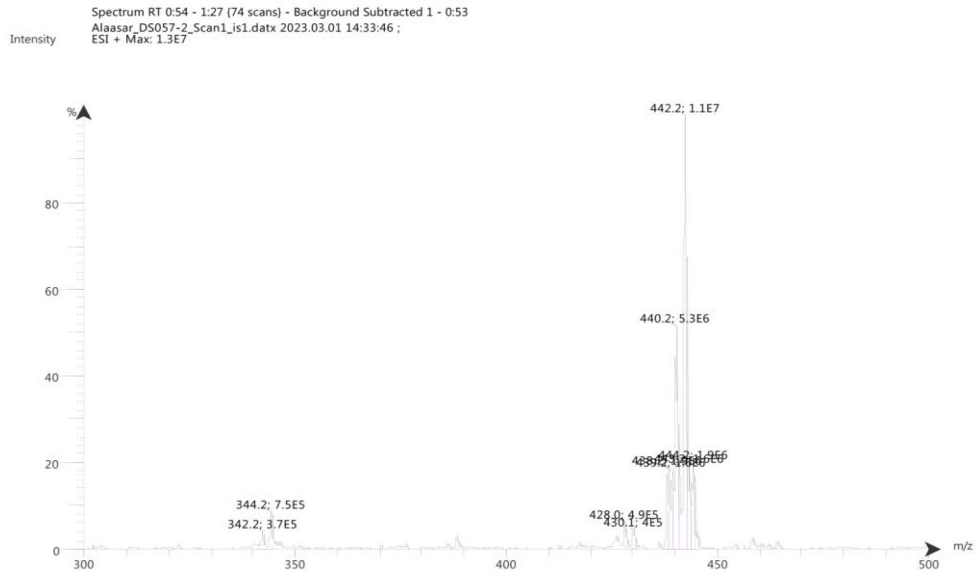

Figure S42. MS chart of compound **9**.

*4-((2-(methoxycarbonyl)-4-(methylselanyl)phenyl)amino)-4-oxobut-2-enoic acid (**10**)*

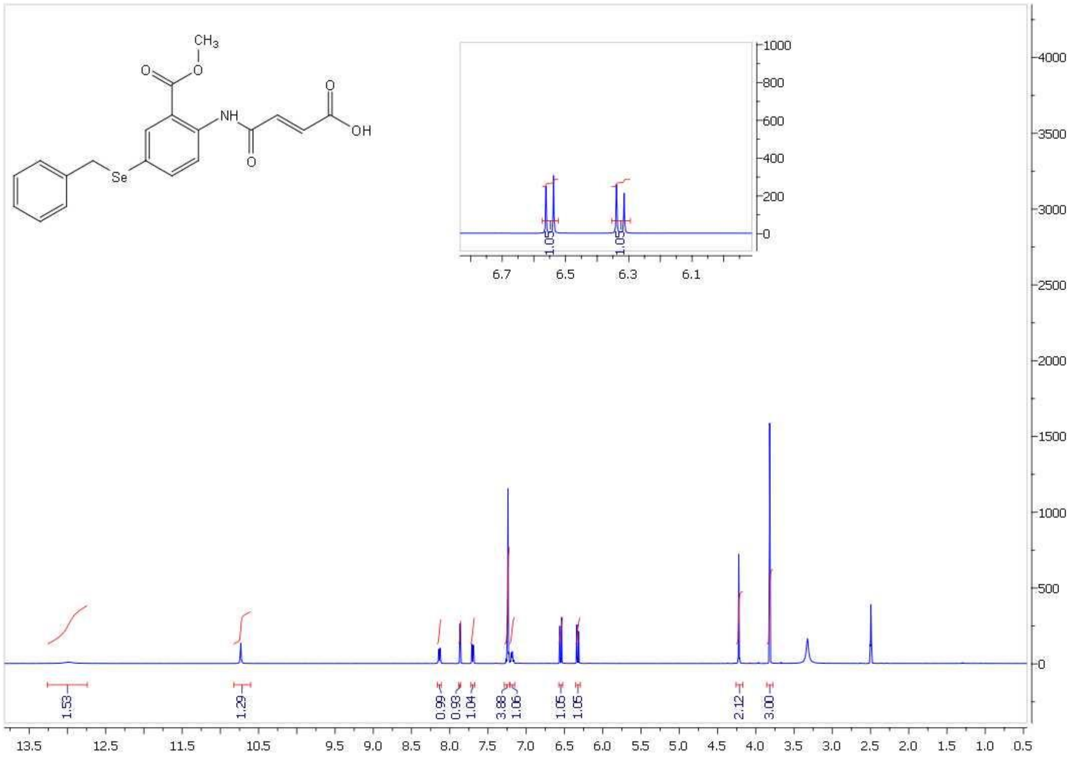

Figure S43. ^{1}H NMR chart of compound **10**.

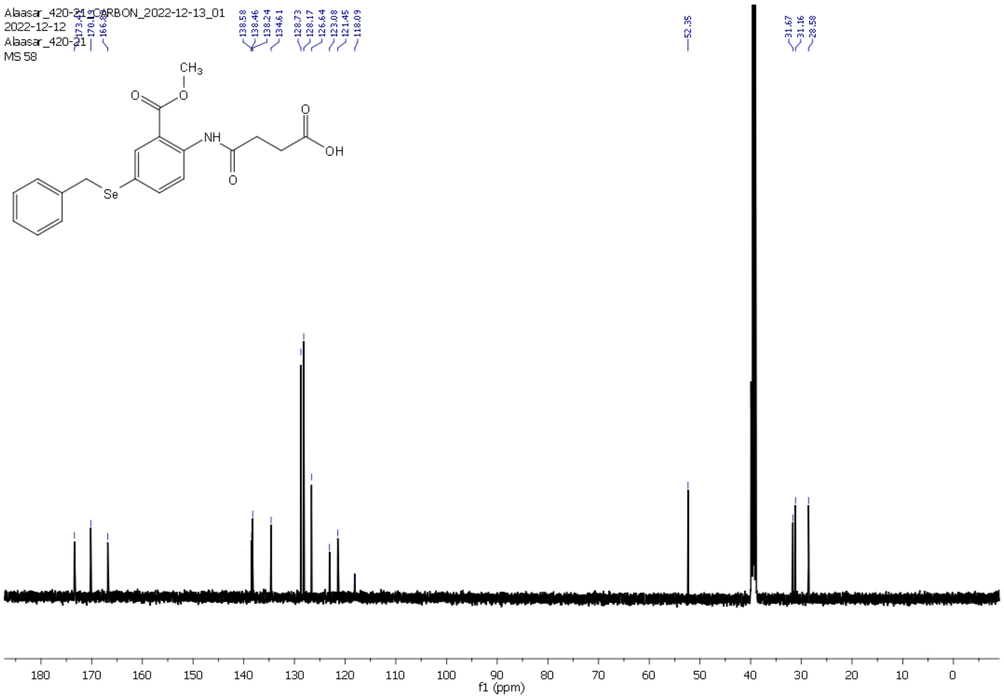

Figure S44. ^{13}C NMR chart of compound **10**.

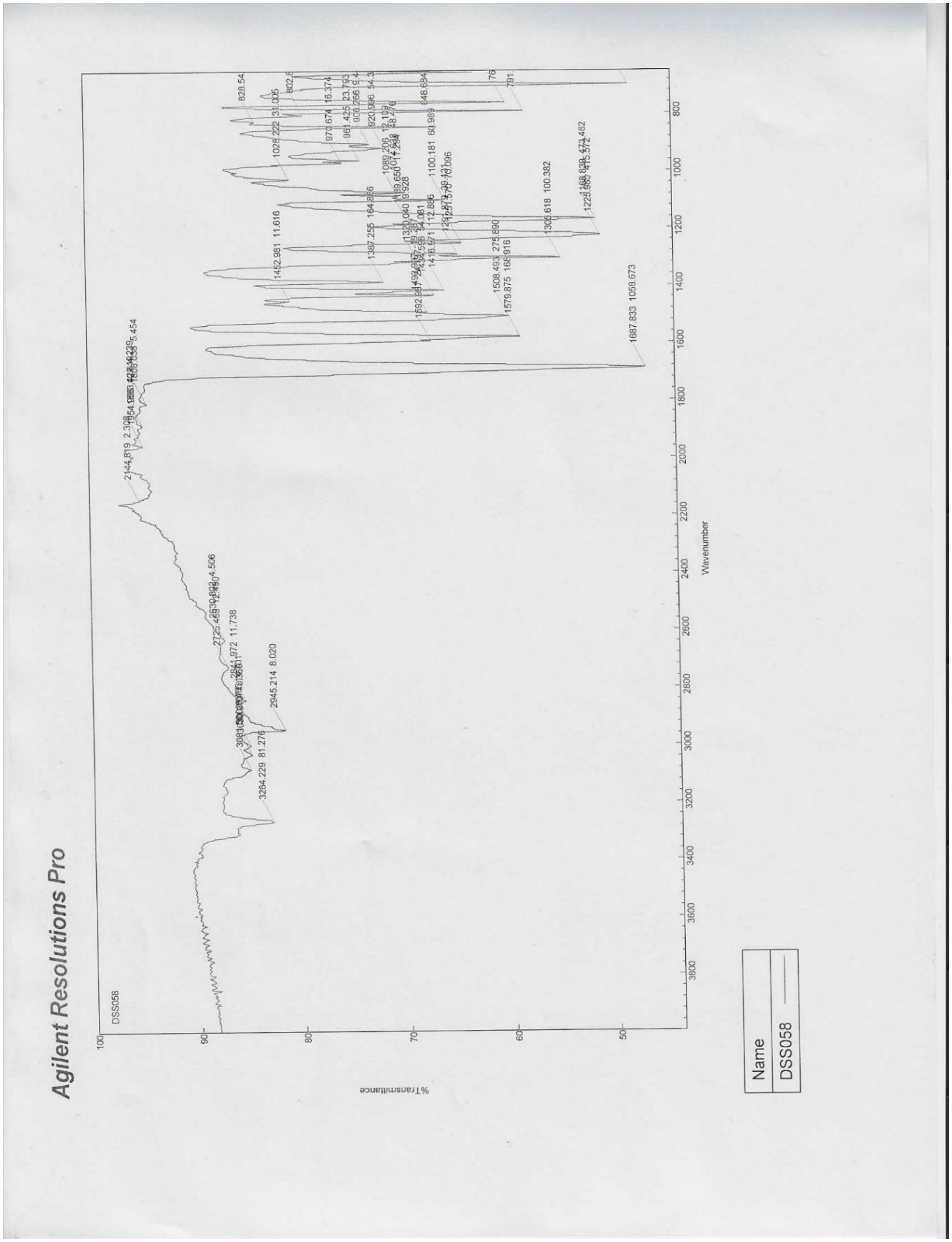

Figure S45. IR chart of compound **10**.

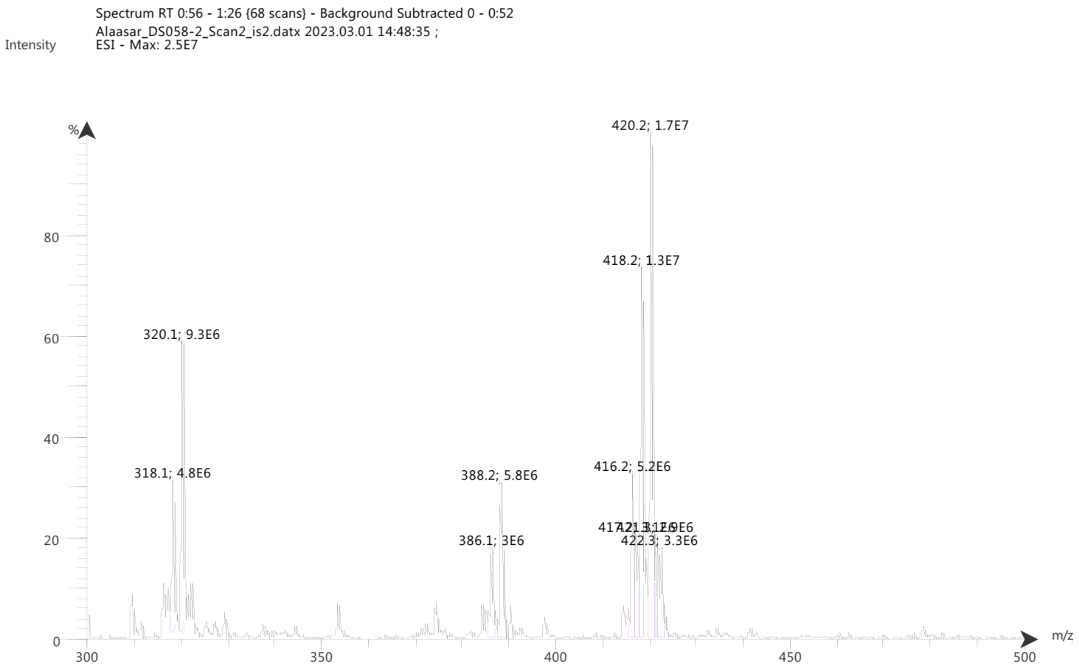

Figure S46. MS chart of compound **10**.

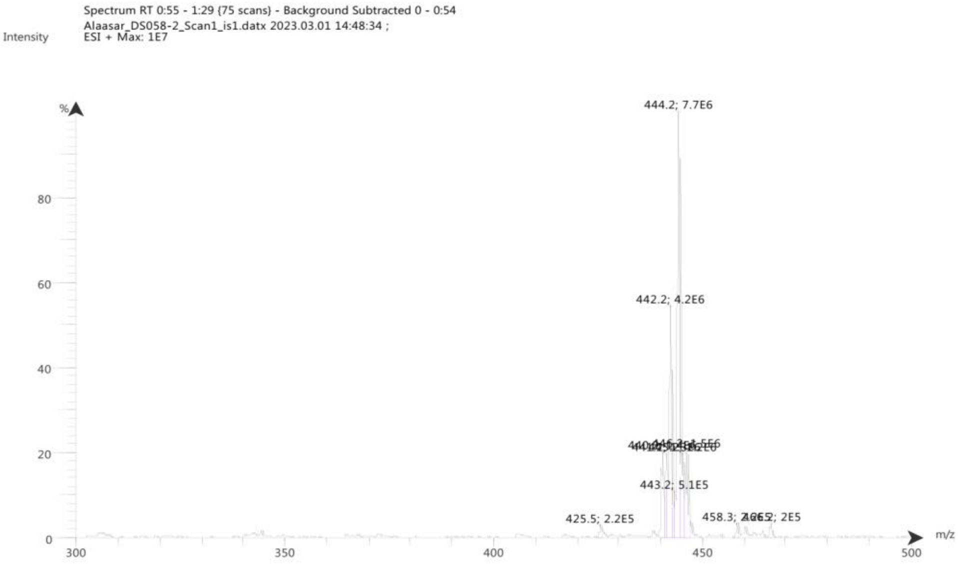

Figure S47. MS chart of compound **10**.

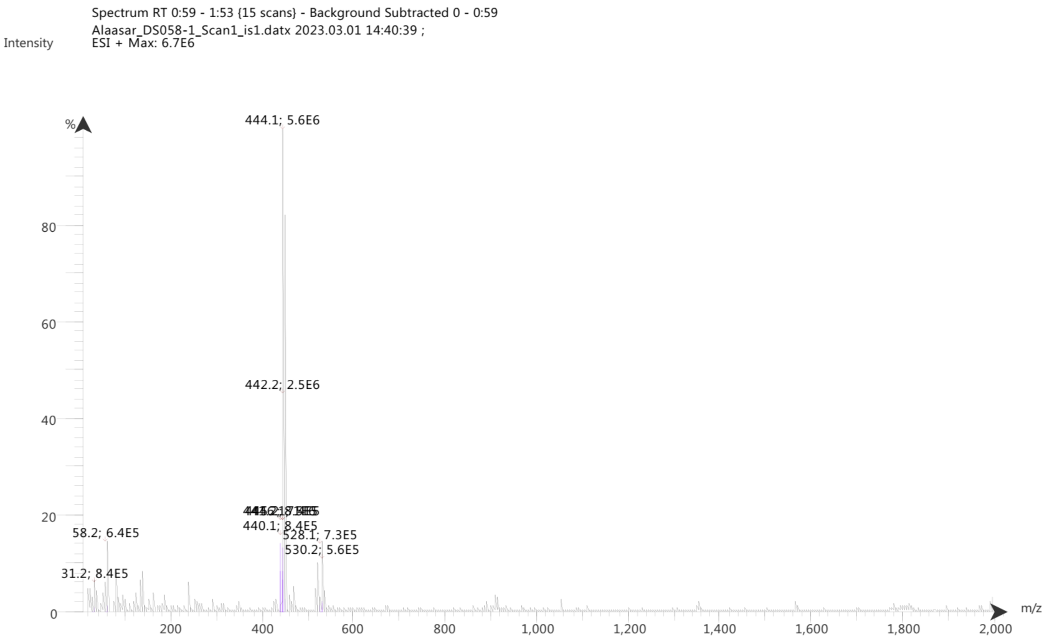

Figure S48. MS chart of compound **10**.

*4-((2-(methoxycarbonyl)-4-(methylselanyl)phenyl)amino)-4-oxobut-2-enoic acid (**11**)*

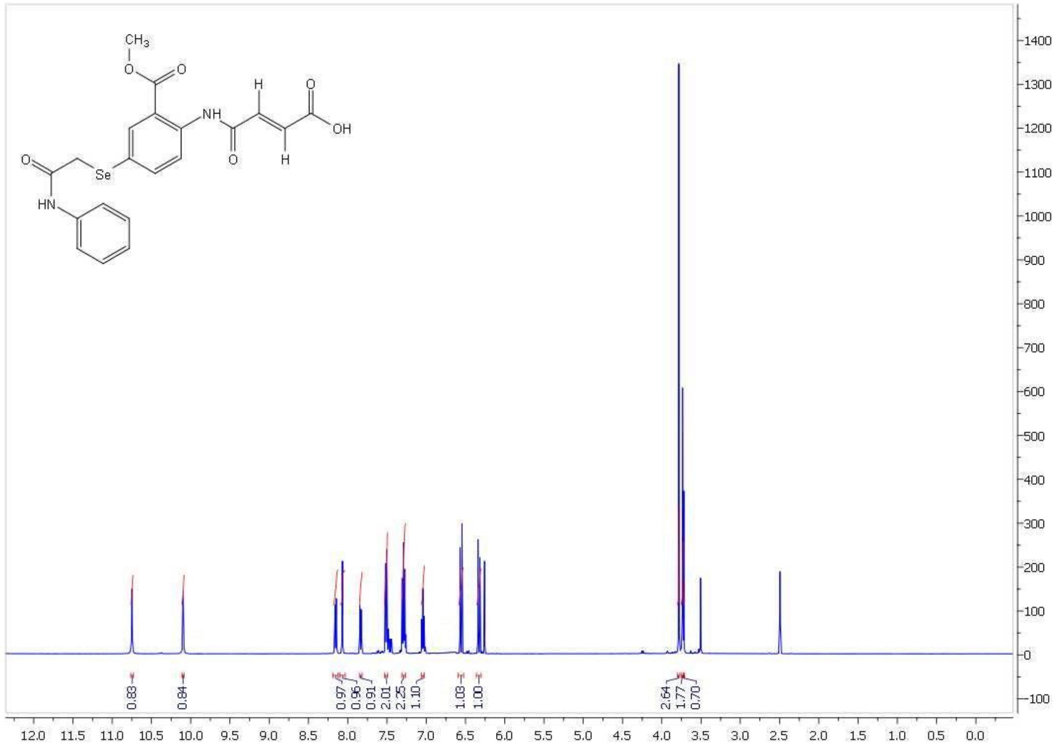

Figure S49. ^{1}H NMR chart of compound **11**.

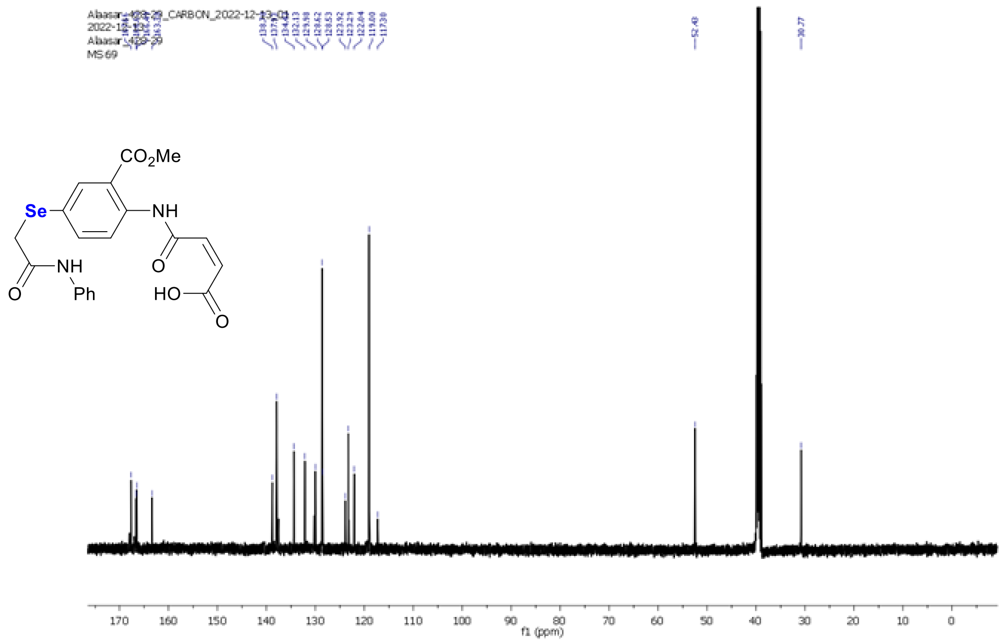

Figure S50. ^{13}C NMR chart of compound **11**.

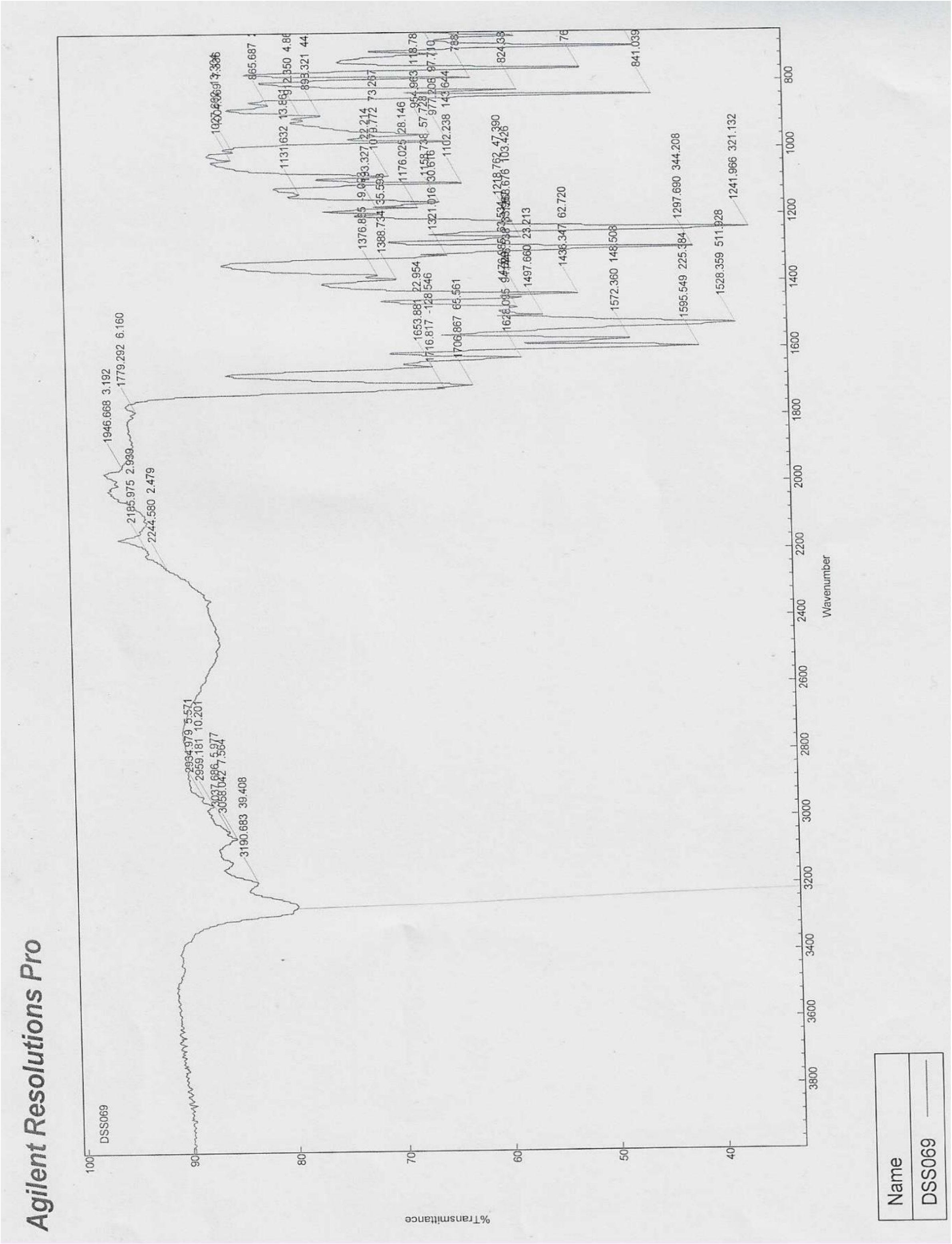

Figure S51. IR chart of compound **11**.

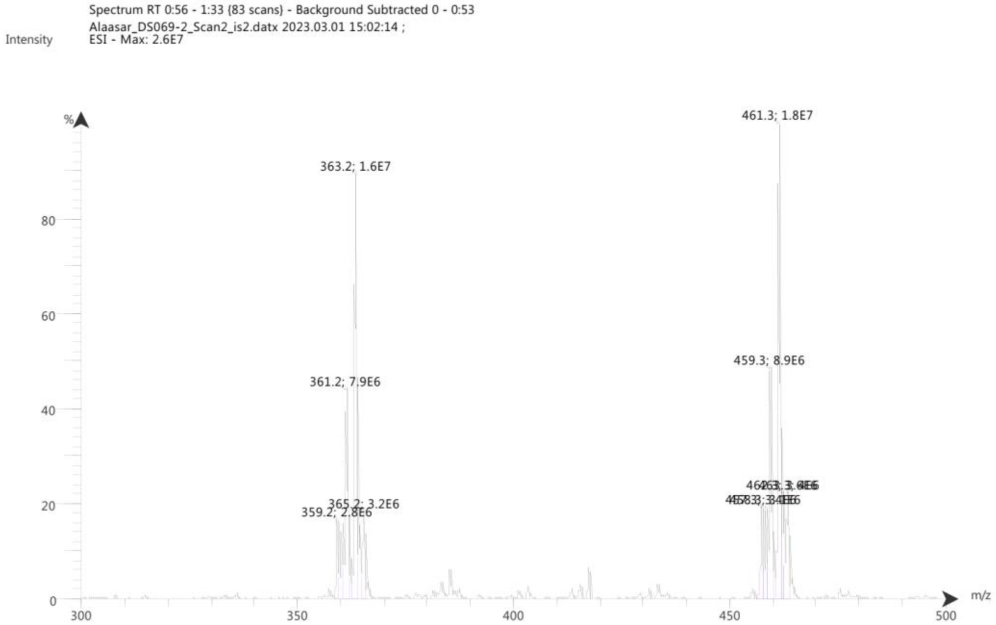

Figure S52. MS chart of compound **11**.

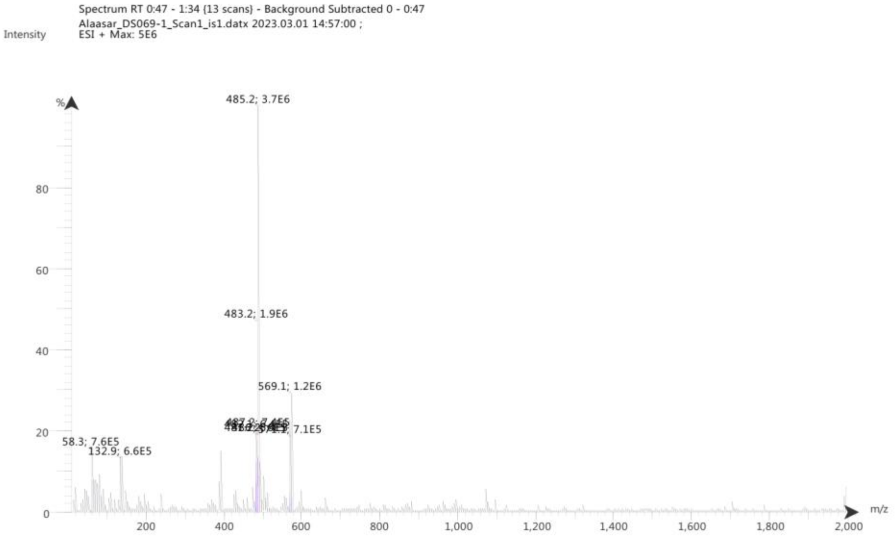

Figure S53. MS chart of compound **11**.

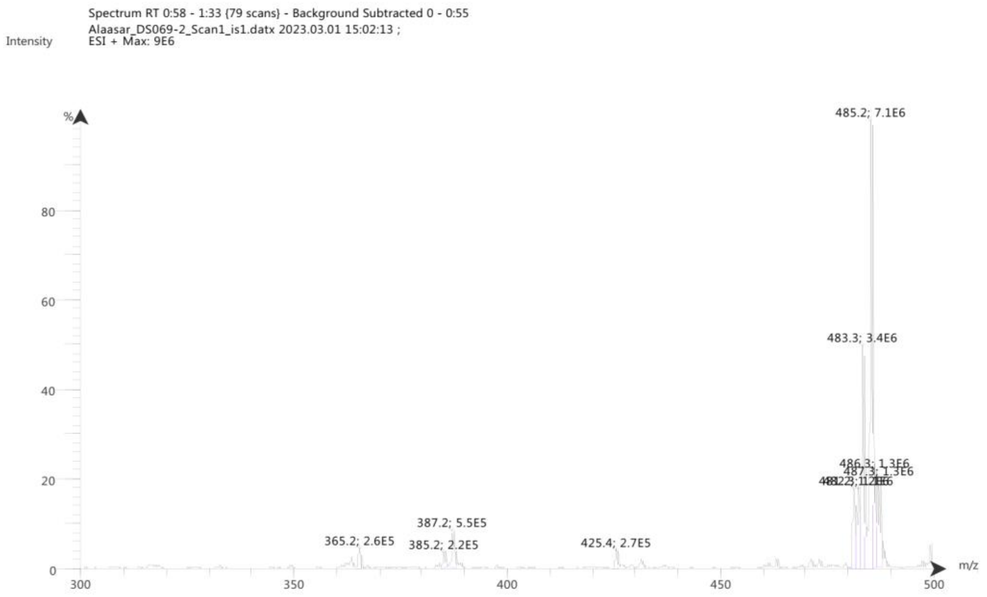

Figure S54. MS chart of compound **11**.

4-((2-(methoxycarbonyl)-4-(methylselanyl)phenyl)amino)-4-oxobut-2-enoic acid ***(12)***

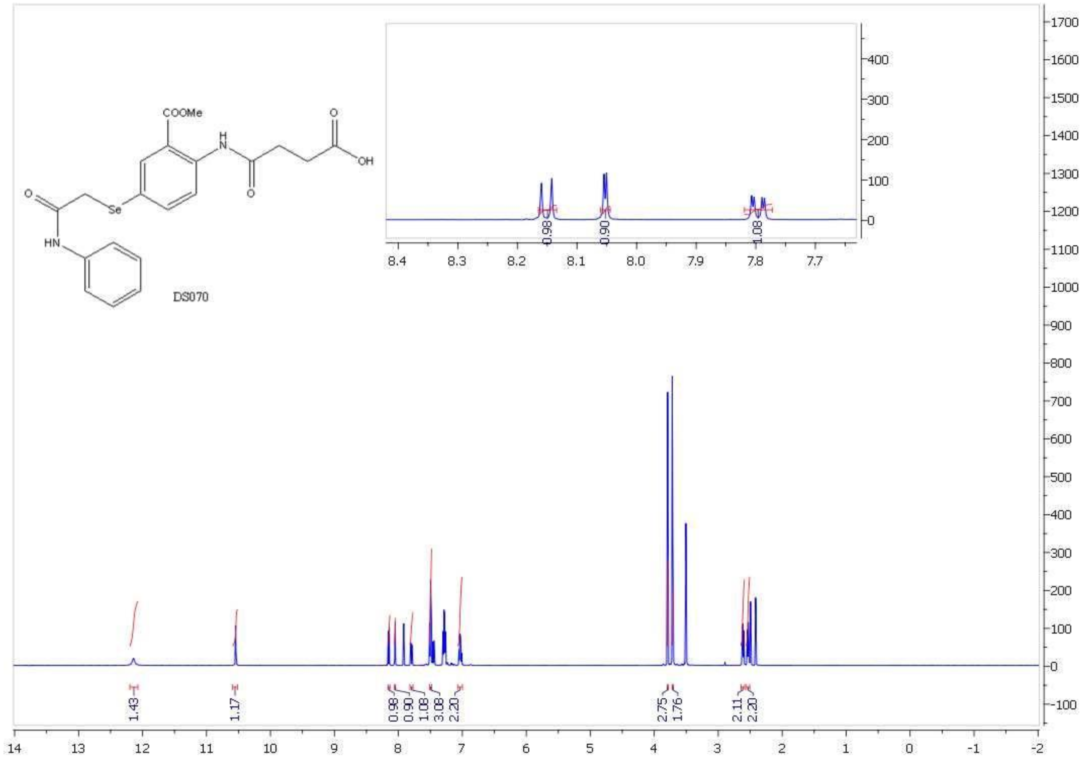

Figure S55. ^{1}H NMR chart of compound **12**.

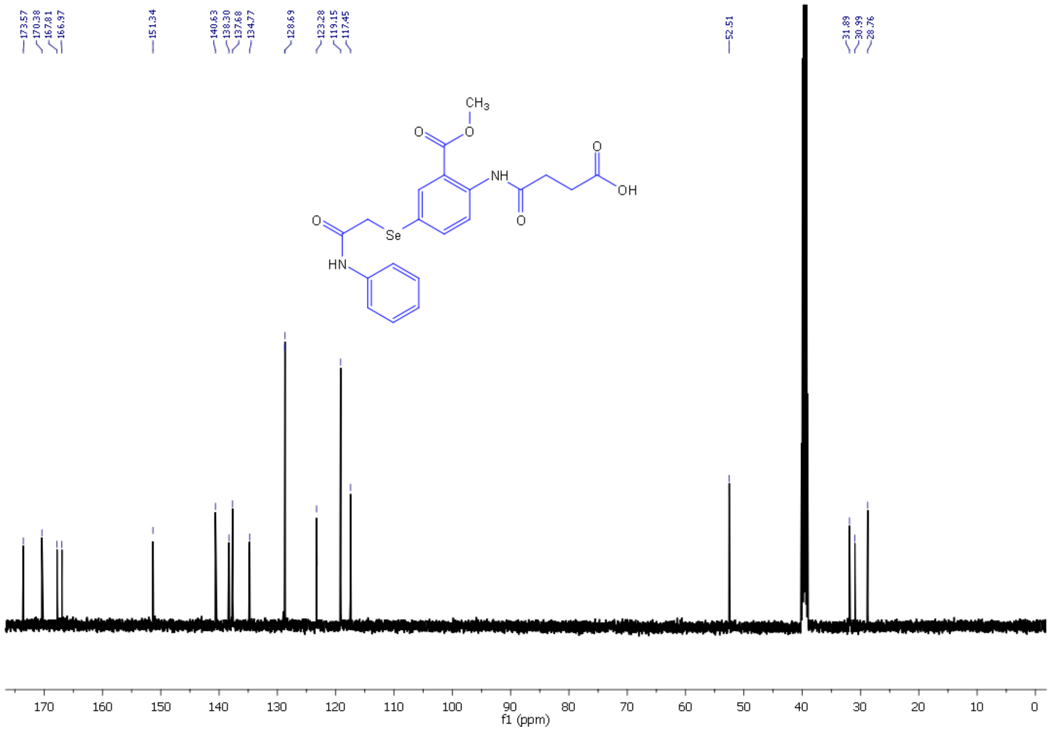

Figure S56. ^{13}C NMR chart of compound **12**.

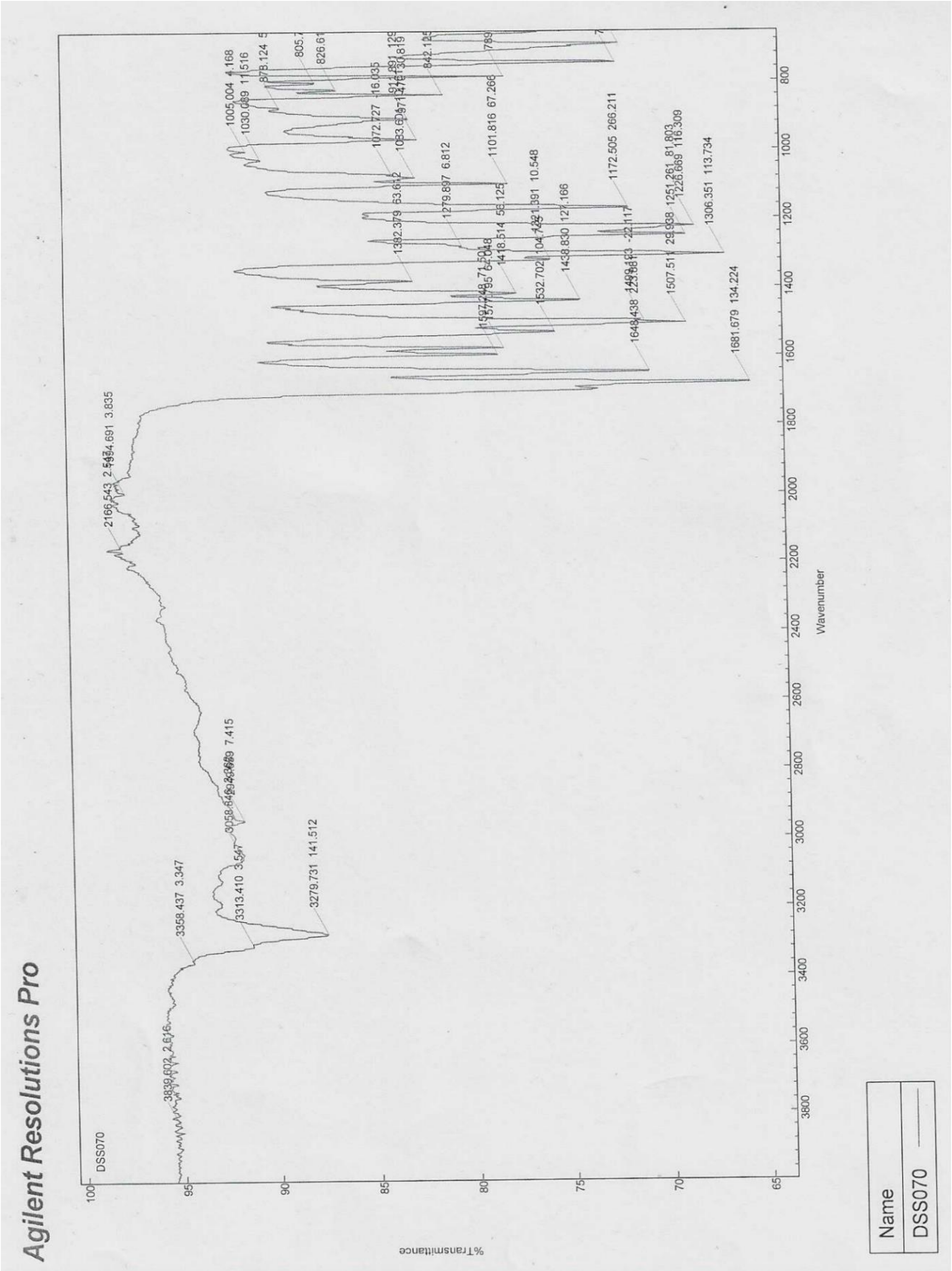

Figure S57. IR chart of compound **12**.

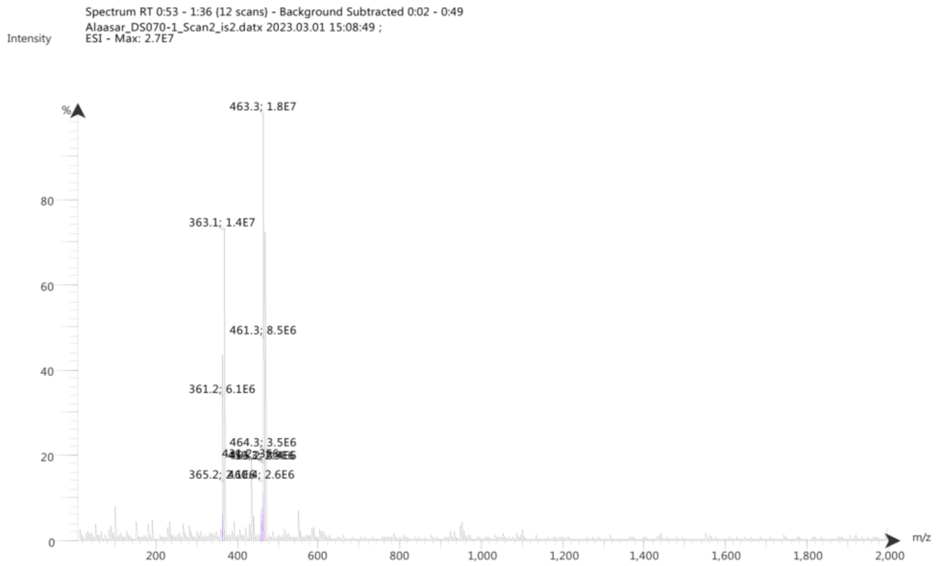

Figure S58. MS chart of compound **12**.

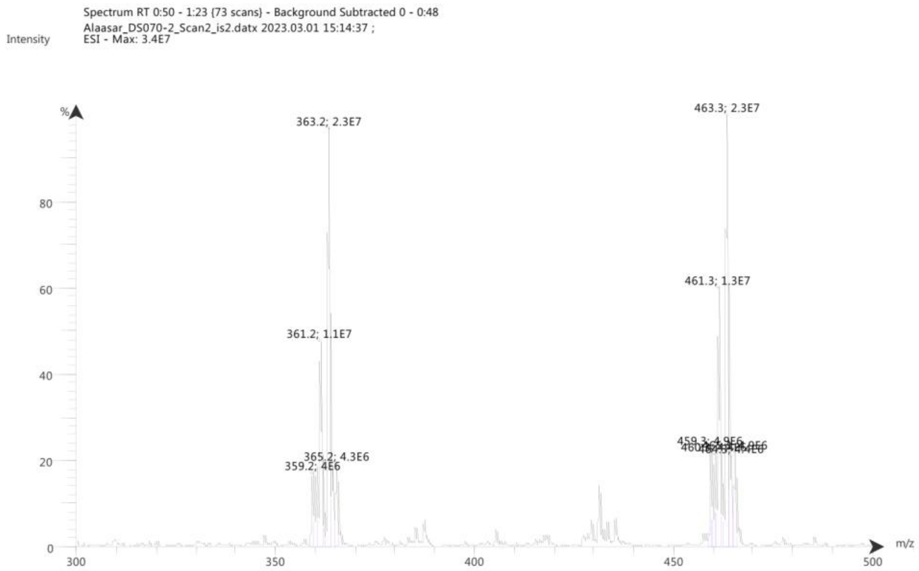

Figure S59. MS chart of compound **12**.

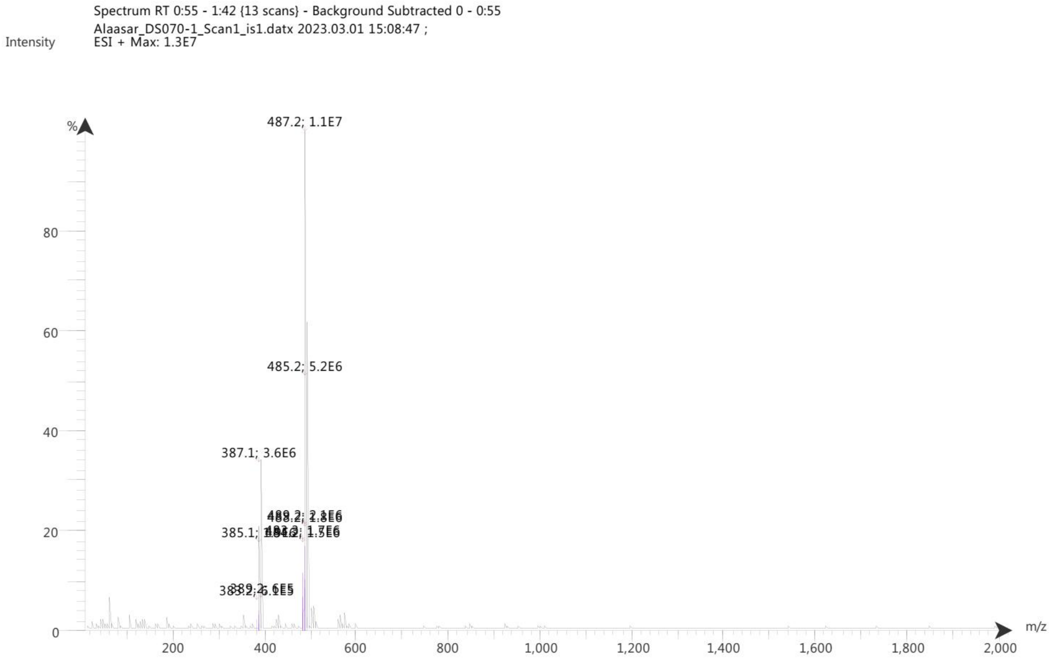

Figure S60. MS chart of compound **12**.

7

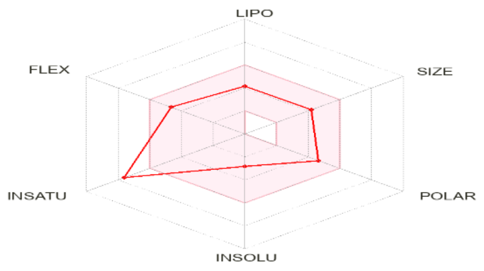

8

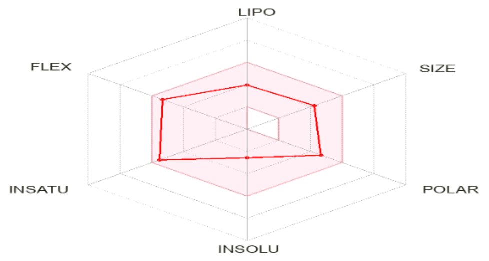

9

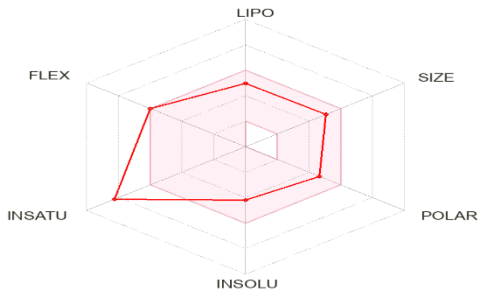

10

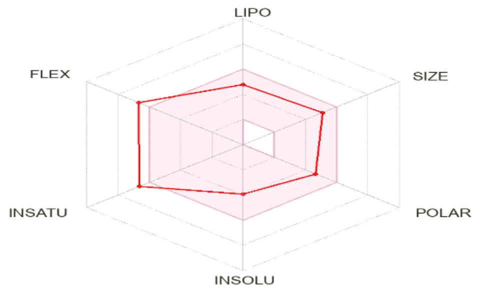

11

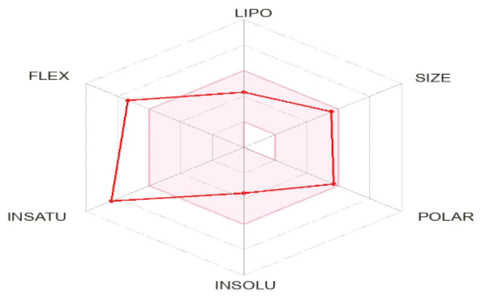

12

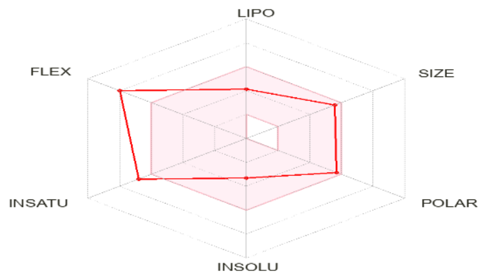

Doxorubicin

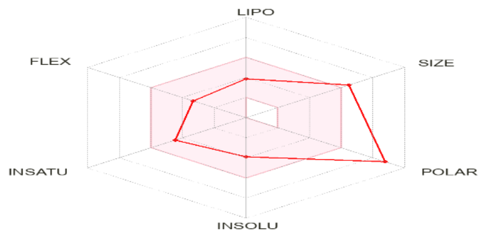

Figure S61. Radar bioavailability for studied compounds in which the area in pink displays specific property optimal range. (LIPO = lipophilicity expressed as XLOGP3 (range from -0.7 to 5.0). SIZE= size expressed as molecular weight (range from 150 g/mol to 500 g/mol). POLAR = polarity expressed as TPSA (topological polar surface area) (range from 20 $Å^2$ to 130 $Å^2$). INSOLU = water insolubility by log S (ESOL) (range from -6 to 0). INSATU = insaturation expressed as for each carbons fraction in sp3 hybridization (range from 0.25 to 1). FLEX = flexibility, expressed as number of rotatable bonds (range from 0 to 9).

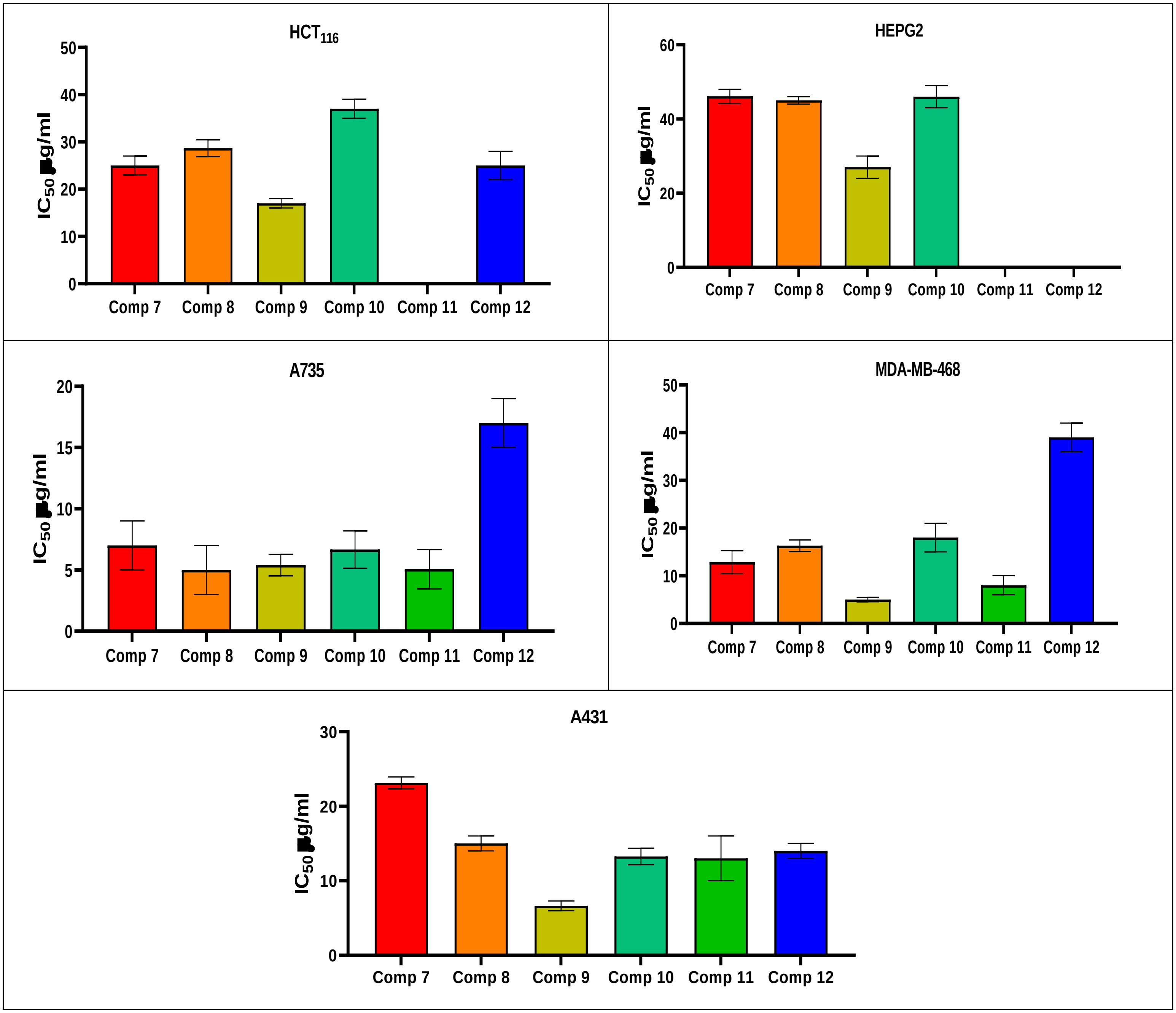

Figure S62. Cytotoxic inhibitory concentration 50 (IC_{50}) evaluation of the investigated compounds against HCT_{116}, HEPG2, A375, MDA-MB-468, and A431 cancer cell lines.

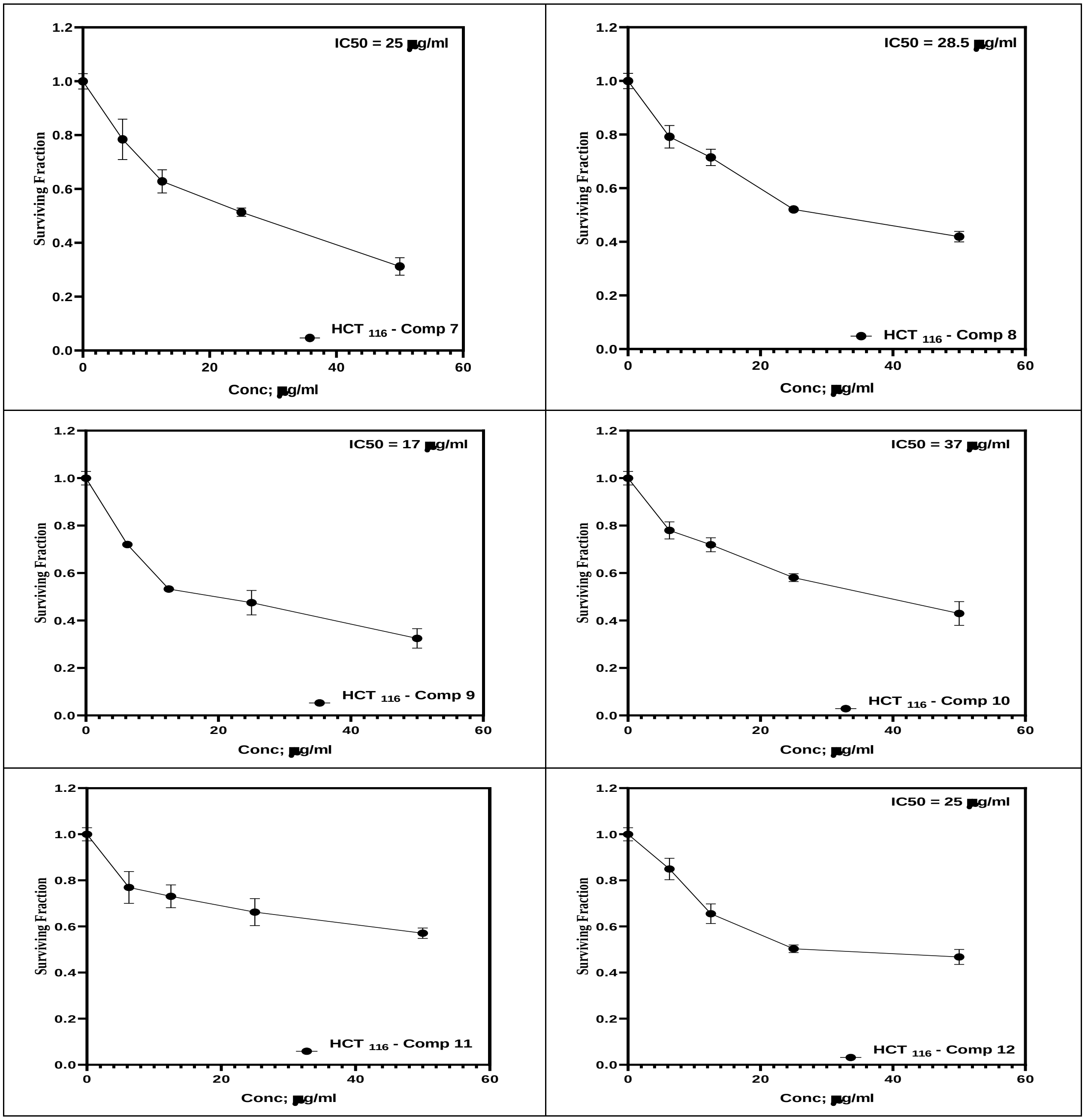

Figure S63. Cytotoxic inhibitory concentration 50 (IC_{50}) evaluation of the investigated compounds against HCT_{116} cancer cell line.

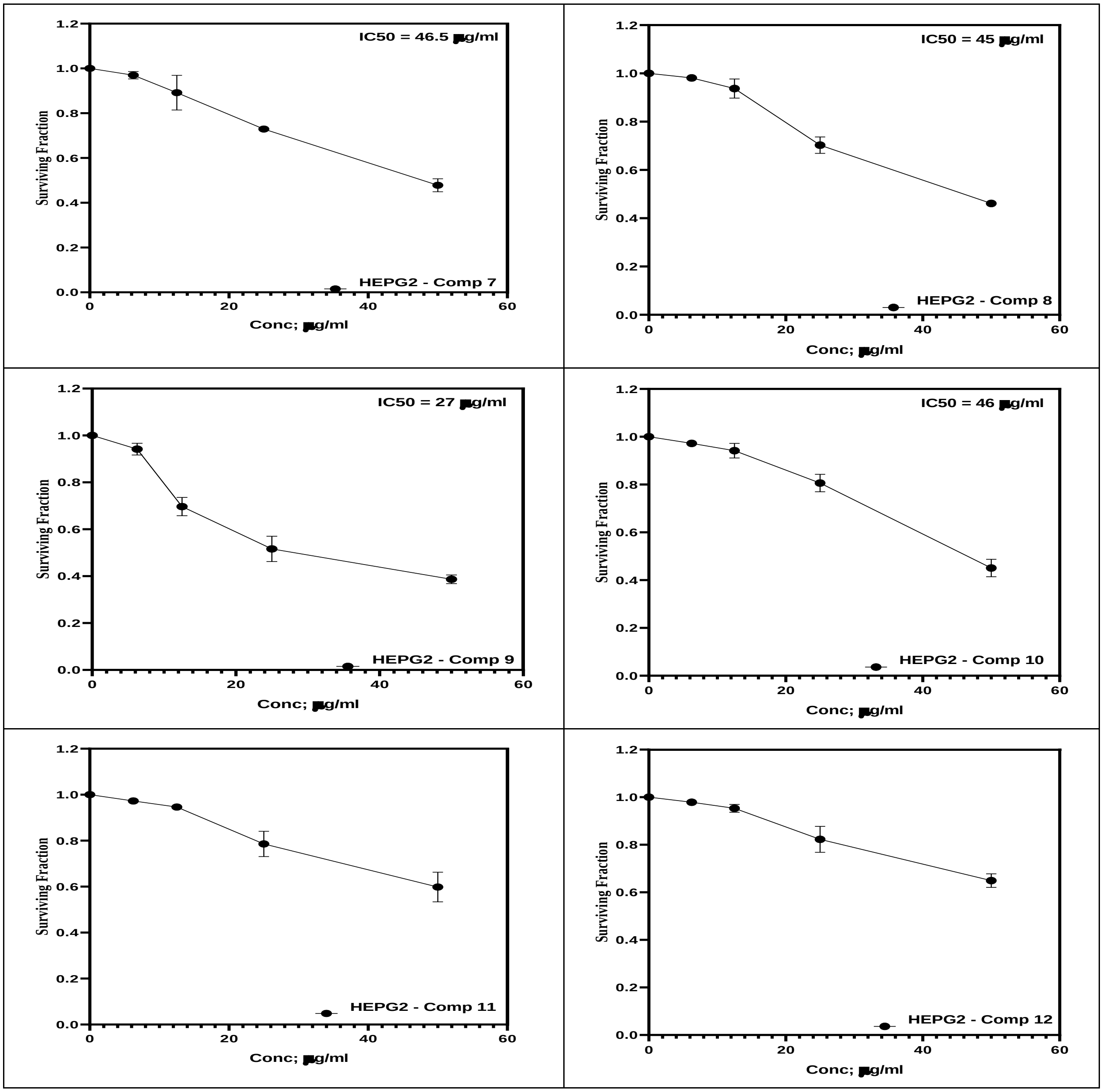

Figure S64. Cytotoxic inhibitory concentration 50 (IC_{50}) evaluation of the investigated compounds against HEPG2 cancer cell line.

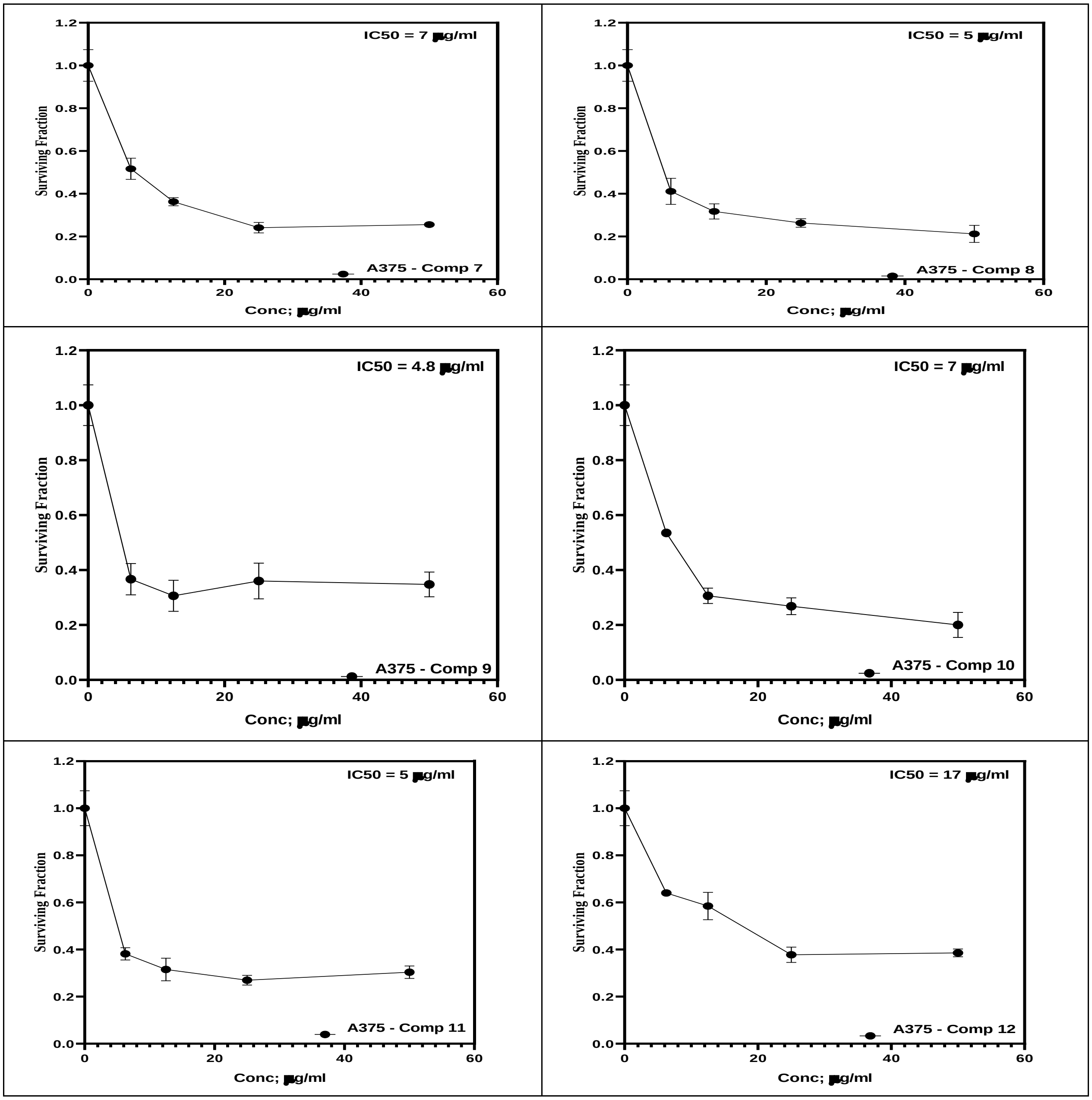

Figure S65. Cytotoxic inhibitory concentration 50 (IC_{50}) evaluation of the investigated compounds against A375 cancer cell line.

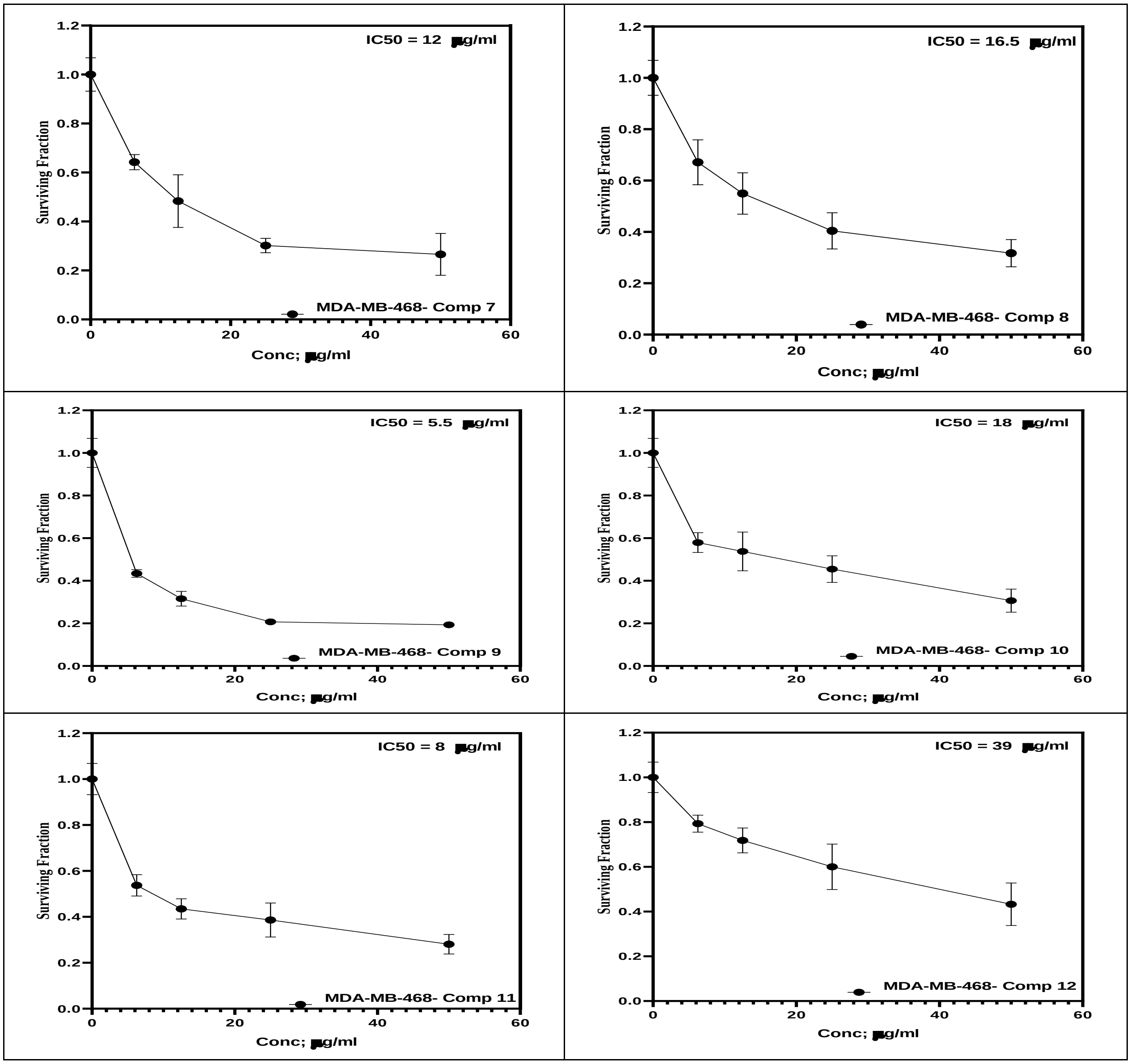

Figure S66. Cytotoxic inhibitory concentration 50 (IC_{50}) evaluation of the investigated compounds against MDA-MB-468 cancer cell line.

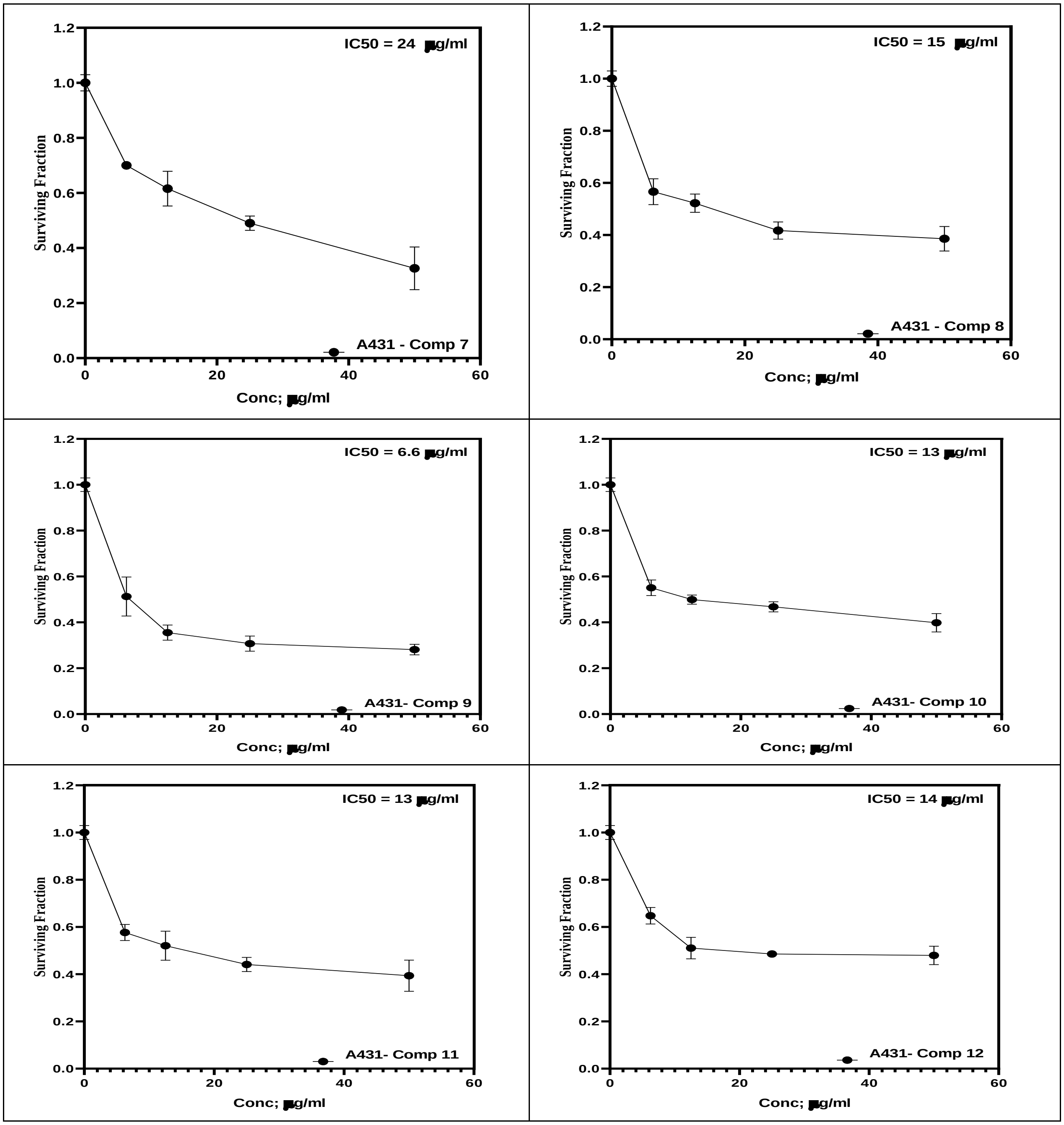

Figure S67. Cytotoxic inhibitory concentration 50 (IC_{50}) evaluation of the investigated compounds against A431 cancer cell line.

Materials and Methods

SI 1. Chemistry

SI 1.1. Material and methods

Melting points were recorded in degree centigrade on a Gallenkamp instrument using the standard open capillary method. The IR spectra were recorded on FTIR 5000 Mattson spectrophotometer. The ^{1}H- and ^{13}C-NMR spectra were recorded in DMSO–d_6 on a Varian 400 and 500 Spectrophotometer (^{1}H: 400 and 500 MHz, ^{13}C: 101 and 125 MHz) at 295 K. The chemical shifts (δ) are given in parts per million (ppm) downfield relative to tetramethyl silane (TMS). Mass Spectra were recorded on Bruker micrOTOFQ II APPI mass spectrometer. Compounds **2–6** were obtained according to our literature reports [1, 2], and the new compounds **7–12** obtained according to our literature reports[3] .

SI 1.2. Synthesis and characterization

The synthesis of OSe maleanilic 7, 9, and 11 and succinanilic 8, 10, and 12 derivatives

Maleic or succinic anhydride (1.3 mmol) was added to OSe amine (1.0 mmol) in methylbenzene (3.0 mL). The solution was stirred for 8 hrs. Then, the formed precipitate was filtered and washed with warm methylbenzene and water. The acids were obtained in enough purity, and no further purifications were needed.

Synthesis of methyl 2-amino-5-selenocyanatobnzoate (2) [1, 4]

Methyl 2-amino-5-selenocyanatobenzoate (**2**) was synthesized from methyl 2-aminobenzoate (12.5 mmol, 1.80 g) with triselenium dicyanide prepared *in situ* from malononitrile (15 mmol, 1.00 g) and selenium dioxide (30 mmol, 3.30 g). It was isolated as reddish solid; yield:3.07 g (96%); m.p. = 118–119 °C; Rf = 0.4 (petroleum ether / ethyl acetate 4:2, *v*/*v*). IR (KBr): ν 3475 (N-H), 3366 (N-H), 2946 (C_{aliph}-H), 2842 (C_{aliph}-H), 2148 (CN), 1691 (C=O), 1551 (C=C), 1253 (C_{Ar}–N), 1085 (C–O), 910, 815 (C-H bending), 556 (C-Se), 536 (C-H rocking), 460; ^{1}H NMR (400 MHz, DMSO-d_6) δ 8.02 (s, 1H, Ar-H), 7.57 (d, J = 8.8 Hz, 1H, Ar-H), 7.08 (s, br, 2H, NH_2), 6.84 (d, *J* = 8.8 Hz, 1H, Ar-H), 3.82 (s, 3H, OCH_3). ^{13}C NMR (101 MHz, DMSO- d_6) δ 166.73, 152.22, 140.09, 137.94, 118.28, 109.59, 105.54, 105.47, 51.73. MS (EI, 70 ev) m/z (%) = 259.35 (M+3H, 2.39), 117 (29.02), 87 (26.6), 75 (2.70), 59 (100.0, base peak).

Supporting Information

Synthesis of dimethyl 5,5'-diselanediylbis(2-aminobenzoate) (**3**) [1, 4]

Compound dimethyl 5,5-diselanediylbis(2-aminobenzoate) (**3**) was synthesized from methyl 2-amino-5-selenocyanatobenzoate (4 mmol, 1.00 g) and sodium hydroxide (4 mmol, 1.60 g) in anhydrous ethanol (20 mL). Dimethyl 5,5'-diselanediylbis(2-aminobenzoate) (**3**) appeared as a single compound on TLC and was isolated as a yellow solid; yield: 1.69 g (92%); m.p. = 138–139 °C; Rf = 0.5 (petroleum ether / ethyl acetate 4:3, *v*/*v*). IR (KBr): ν 3455 (N-H), 3344 (N-H), 2931 (C_{aliph}-H), 2890 (C_{aliph}-H), 2168 (CN), 1684 (C=O), 1560 (C=C), 1603, 1238 (C_{Ar}–N), 1079 (C–O), 811, 784 (C-H bending), 699 (C-H rocking), 533 (C-Se); ^{1}H NMR (400 MHz, DMSO- d_6) δ 7.70 (s, 2H, Ar-H), 7.44 (d, J = 8.6 Hz, 2H, Ar-H), 7.00 (s, 4H, $2NH_2$), 6.77 (d, J = 8.6 Hz, 2H, Ar-H), 3.74 (s, 6H, $2OCH_3$). ^{13}C NMR (101 MHz, DMSO- d_6) δ 167.00, 151.91, 140.57, 138.13, 117.63, 113.57, 108.98, 51.52. MS (EI, 70 ev) m/z (%) = 460.15 (M+H, 20.76), 459.15 (M, 5.20) or 230 (24.42), 119 (9.45), 91 (100.0, base peak), 65 (8.88).

Synthesis of methyl 2-amino-5-(methylselanyl) benzoate (**4**)[1, 4]

Compound **4** was synthesized from dimethyl 5,5'-diselanediylbis (2-aminobenzoate) (**3**) (2 mmol, 916 mg) and methyl iodide (4.4 mmol, 0.27 mL). It was isolated as brown oil; yield: 402 mg (82%); Rf = 0.6 (petroleum ether / ethyl acetate 4:2, *v*/*v*). IR (KBr): ν 3474 (N-H), 3364 (N-H), 2948 (C_{aliph}-H), 2926 (C_{aliph}-H), 1686 (C=O), 1607, 1292 (C_{Ar}–N), 1110 (C–O), 815, 789, 696 (C-H bending), 559 (C-Se); ^{1}H NMR (400 MHz, DMSO- d_6) δ 7.82 (s, 1H, Ar-H), 7.40 (d, J = 8.6 Hz, 1H, Ar-H), 6.77 (d, J = 8.6 Hz, 1H, Ar-H), 6.75 (s, 2H, NH_2), 3.62 (s, 3H, OCH_3), 2.22 (s, 3H, CH_3). ^{13}C NMR (101 MHz, DMSO- d_6) δ 167.70, 151.00, 138.90, 135.03, 118.21, 113.98, 109.87, 51.98, 9.17. MS (EI, 70 ev) m/z (%) = 245.10 (M, 100.0, base peak), 230 (38.06),186 (10.46), 170 (35.08), 91 (91.10).

Synthesis of methyl 2-amino-5-(benzylselanyl) benzoate (5)[1, 4]

Compound **5** was synthesized from dimethyl 5,5'-diselanediylbis(2-aminobenzoate) (**3**) (2 mmol, 916 mg) and benzyl chloride (4.4 mmol, 0.50 mL). It was isolated as light brown solid; yield: 597 mg (93%); m.p. = 78 °C; Rf = 0.6 (petroleum ether / ethyl acetate 4:3, *v*/*v*). IR (KBr): ν 3462 (N-H), 3353 (N-H), 2977 (C_{aliph}-H), 2946 (C_{aliph}-H), 1672 (C=O), 1621, 1436, 1234 (C_{Ar}-N), 1114 (C-O), 810 (C-H bending), 692 (C-H rocking), 595 (C-Se); ^{1}H NMR (400 MHz, DMSO- d_6) δ 7.70 (s, 1H, Ar-H), 7.30 (d, J = 8.6 Hz, 1H, Ar-H), 7.23 (dd, J = 8.6 Hz, J = 2.1 Hz, 2H, Ar-H),

7.19 (d, J = 8.7 Hz, 2H, Ar-H), 7.14 (d, J = 8.6 Hz, 2H, Ar-H), 6.82 (s, 2H, NH_2), 4.00 (s, 2H, $SeCH_2$), 3.77 (s, 3H, OCH_3); ^{13}C NMR (101 MHz, DMSO- d_6) δ 167.69, 151.53, 141.06, 139.66, 137.95, 129.24, 128.65, 126.98, 117.87, 112.42, 109.68, 51.94, 32.75. MS (EI, 70 ev) m/z (%) = 321.20 (M, 46.87), 244.10 (2.77), 230 (34.88), 150.15 (1.02), 91.05 (100.0, base peak).

Synthesis of methyl 2-amino-5-((2-oxo-2-(phenylamino) ethyl) selanyl) benzoate (6)[1, 4]

Compound **6** was synthesized from dimethyl 5,5'-diselanediylbis (2-aminobenzoate) (**3**) (2.0 mmol, 916 mg) and 2-chloro-N-phenylacetamide (4.4 mmol, 744 mg). It was isolated as violet solid; yield: 699 mg (96%); m.p. = 126–128 °C; Rf = 0.50 (petroleum ether / ethyl acetate 4:2, *v*/*v*). IR (KBr): ν 3466 (N-H), 3354 (N-H), 3246 (N-H), 2945 (C_{aliph}-H), 1678, 1594 (C=O), 1542 (C=C), 1439, 1238 (C_{Ar}-N), 1090 (C-O), 752 (C-H bending), 695, 552 (C-Se), 539 (C-H rocking); ^{1}H NMR (400 MHz, DMSO- d_6) δ 9.99 (s, 1H, NH), 7.96 (s, 1H, Ar-H), 7.53 (d, J = 8.6 Hz, 2H, ArH), 7.48 (d, J = 8.6 Hz, 1H, ArH), 7.30 (dd, J = 8.6 Hz, J = 2.1 Hz, 2H, Ar-H), 7.04 (t, 1H, J = 8.6 Hz, Ar-H), 6.86 (s, 2H, NH_2), 6.75 (d, J = 8.6 Hz, 1H, Ar-H), 3.74 (s, 3H, OCH_3), 3.42 (s, 2H, $SeCH_2$); ^{13}C NMR (101 MHz, DMSO- d_6) δ 168.65, 167.65, 151.81, 141.13, 139.49, 138.20, 129.14, 123.74, 119.62, 117.89, 111, 109.71, 51.93, 32.33. MS (EI, 70 ev) m/z (%) = 364.20 (M, 100.0, base peak), 230 (40.48), 91.10 (31), 77.05 (24.13), 59 (14.56).

SI 2. GI% of the investigated organoselenium compounds (7-12) against a series of cancer and normal cell lines

The antitumor activities of the investigated organoselenium compounds (**7-12**) against all tested cell lines were evaluated by sulphorhodamine-B (SRB) assay [5]. Briefly, cells were seeded at a density of 3×10^3 cells/well in 96-well microtiter plates. They were left to attach for 24 h before incubation with the aforementioned compounds. Next, cells were treated with 100 µg/mL for **7-12** candidates.

For each concentration, three wells were used and incubation was continued for 48 h. DMSO was used as a control vehicle (1 % v/v). At the end of incubation, cells were fixed with 20% trichloroacetic acid, and stained with 0.4% SRB dye. The optical density (O.D.) of each well was measured spectrophotometrically at 570 nm using an ELISA microplate reader (TECAN sunrise™, Germany). The mean survival fraction at each drug concentration was calculated as follows: O.D. of the treated cells/O.D. of the control cells. The IC_{50} (concentration that produces

50% of cell growth inhibition) value of each drug was calculated using sigmoidal dose-response curve-fitting models (Graph Pad Prizm software, version 8).

SI 3. Cytotoxic inhibitory concentration 50 (IC_{50}) evaluation against HCT_{116}, HEPG2, A375, MDA-MB-468, and A431 cancer cell lines

The antitumor activities of the investigated organoselenium compounds (**7-12**) against HCT116, HEPG2, A375, MDA-MB-468, and A431 cells, were evaluated by sulphorhodamine-B (SRB) assay[5]. Briefly, cells were seeded at a density of 3×10^3 cells/well in 96-well microtiter plates. They were left to attach for 24 h before incubation with the aforementioned compounds. Next, cells were treated with different concentrations of 62.5, 12.5, 25, and 50 µg/mL for **3-17** candidates.

For each concentration, three wells were used and incubation was continued for 48 h. DMSO was used as a control vehicle (1 % *v/v*). At the end of incubation, cells were fixed with 20% trichloroacetic acid, and stained with 0.4% SRB dye. The optical density (O.D.) of each well was measured spectrophotometrically at 570 nm using an ELISA microplate reader (TECAN sunrise™, Germany). The mean survival fraction at each drug concentration was calculated as follows: O.D. of the treated cells/O.D. of the control cells. The IC_{50} (concentration that produces 50% of cell growth inhibition) value of each drug was calculated using sigmoidal dose-response curve-fitting models (Graph Pad Prizm software, version 8).

SI 4. Protein expression of the apoptosis-related genes

The microplate provided in this kit has been pre-coated with an antibody specific to P53, BAX, Caspases 3, 6, 8, and 9, BCL-2, MMP2, and MMP9. Standards or samples are then added to the appropriate microplate wells with a biotin-conjugated antibody specific to P53, BAX, Caspases 3, 6, 8, and 9, BCL-2, MMP2, and MMP9. Next, Avidin conjugated to Horseradish Peroxidase (HRP) is added to each microplate well and incubated. After the TMB substrate solution is added, only those wells that contain P53, BAX, Caspases 3, 6, 8, and 9, BCL-2, MMP2, and MMP9, biotin-conjugated antibody, and enzyme-conjugated Avidin will exhibit a color change. The enzyme-substrate reaction is terminated by the addition of sulphuric acid solution and the color change is measured spectrophotometrically at a wavelength of 450 nm ± 10 nm. The concentration of P53, BAX, Caspases 3, 6, 8, and 9, BCL-2, MMP2, and MMP9 in the samples is

then determined by comparing the O.D. of the samples to the standard curve. Average the duplicate readings for each standard, control, and sample, and subtract the average zero standard optical density. Construct a standard curve by plotting the mean O.D. and concentration for each standard and draw a best-fit curve through the points on the graph or create a standard curve on log-log graph paper with P53, BAX, Caspases 3, 6, 8, and 9, BCL-2, MMP2, and MMP9 concentration on the y-axis and absorbance on the x-axis. Using some plot software, for instance, Curve Expert 1.30, is also recommended. If samples have been diluted, the concentration read from the standard curve must be multiplied by the dilution factor.

Procedure

1. Determine wells for diluted standard, blank, and samples (**8**, **9**, **10**, and **11**). Prepare 7 wells for standard, 1 well for blank. Add 100 µL each of dilutions of standard, blank, and samples into the appropriate wells. Cover with the Plate sealer. Incubate for 1 h at 37 ^{o}C.

2. Remove the liquid from each well, don’t wash.

3. Add 100 µL of Detection Reagent A working solution to each well, cover the wells with the plate sealer, and incubate for 1 h at 37 ^{o}C.

4. Aspirate the solution and wash with 350 µL of 1× Wash Solution to each well using a squirt bottle, multi-channel pipette, manifold dispenser, or autowasher, and let it sit for 1~2 min. Remove the remaining liquid from all wells completely by snapping the plate onto absorbent paper. Totally wash 3 times. After the last wash, remove any remaining Wash Buffer by aspirating or decanting. Invert the plate and blot it against absorbent paper.

5. Add 100 µL of Detection Reagent B working solution to each well, cover the wells with the plate sealer, and incubate for 30 min at 37 ^{o}C.

6. Repeat the aspiration/wash process for a total of 5 times as conducted in step 4.

7. Add 90 µL of Substrate Solution to each well. Cover with a new Plate sealer. Incubate for 10-20 min at 37 ^{o}C (Don't exceed 30 min). Protect from light. The liquid will turn blue with the addition of a Substrate Solution.

8. Add 50 µL of Stop Solution to each well. The liquid will turn yellow with the addition of the stop solution. Mix the liquid by tapping the side of the plate. If the color change does not appear uniform, gently tap the plate to ensure thorough mixing.

9. Remove any drop of water and fingerprint on the bottom of the plate and confirm there is no bubble on the surface of the liquid. Then, run the microplate reader and conduct measurement at 450 nm immediately.

References

1. B. Al-Abdallah, Y. S. Al-Faiyz and S. Shaaban, *Biomolecules*, 2022, **12**, 1765.
2. M. Sak, Y. S. Al-Faiyz, H. Elsawy and S. Shaaban, *Antioxidants*, 2022, **11**, 1231.
3. S. Shaaban, Y. S. Al-Faiyz, G. M. Alsulaim, M. Alaasar, N. Amri, H. Ba-Ghazal, A. A. Al-Karmalawy and A. Abdou, *Inorganics*, 2023, **11**, 321.
4. B. Al-Abdallah, Y. S. Al-Faiyz and S. Shaaban, *Inorganics*, 2022, **10**, 246.
5. P. Skehan, R. Storeng, D. Scudiero, A. Monks, J. McMahon, D. Vistica, J. T. Warren, H. Bokesch, S. Kenney and M. R. Boyd, *JNCI: Journal of the National Cancer Institute*, 1990, **82**, 1107-1112.
